# Global, regional, and national burden of headache disorders, 1990–2023: a systematic analysis for the Global Burden of Disease Study 2023

**DOI:** 10.1016/S1474-4422(25)00402-8

**Published:** 2025-12

**Authors:** GBD 2023 Headache Collaborators, Andreas Kattem Hus⊘y, Yvonne Yiru Xu, Jaimie D Steinmetz, Mohammad Amin Aalipour, Hasan Aalruz, Deldar Morad Abdulah, Richard Gyan Aboagye, Dariush Abtahi, Samir Abu Rumeileh, Salahdein Aburuz, Qorinah Estiningtyas Sakilah Adnani, Obed Adonteng-Kissi, Giuseppina Affinito, Danish Ahmad, Negar Sadat Ahmadi, Ali Ahmed, Asma Ahmed, Shahzaib Ahmed, Mohammad Ahmmad Mahmoud Al Zoubi, Sawsan Alabbad, Yazan Al-Ajlouni, Mohammed Albashtawy, Fadwa Naji Alhalaiqa, Ashraf Alhumaidi, Mohammed Usman Ali, Syed Shujait Ali, Montaha Al-Iede, Joseph Uy Almazan, Najim Z Alshahrani, Awais Altaf, Mohammad Al-Wardat, Yaser Mohammed Al-Worafi, Karem H Alzoubi, Sohrab Amiri, Ganiyu Adeniyi Amusa, David B Anderson, Abhishek Anil, Jalal Arabloo, Aleksandr Y Aravkin, Demelash Areda, Mahsa Asadi Anar, Mohammad Asghari-Jafarabadi, Sait Ashina, Khursheed Aurangzeb, Arian Azadnia, Ahmed Y Azzam, Youngoh Bae, Razieh Bahreini, Soham Bandyopadhyay, Hiba Jawdat Barqawi, Azadeh Bashiri, Rehana Basri, Mohammad-Mahdi Bastan, Jina Behjati, Maryam Bemanalizadeh, Jeetendra Bhandari, Sonu Bhaskar, Gurjit Kaur Bhatti, Jasvinder Singh Bhatti, Rajbir Bhatti, Bijit Biswas, Bruno Bizzozero-Peroni, Archith Boloor, Meriem Boukhiam, Yasser Bustanji, Sanjay C J, Luis Alberto Cámera, Edoardo Caronna, Ana Paula Carvalho-e-Silva, Sandip Chakraborty, Vijay Kumar Chattu, Anis Ahmad Chaudhary, Patrick R Ching, Hongyuan Chu, Josielli Comachio, Daniela Contreras, Natalia Cruz-Martins, Omid Dadras, Xiaochen Dai, Emanuele D'Amico, Anh Kim Dang, Lucio D'Anna, Sindhura Deekonda, Pouria Delbari, Andreas K Demetriades, Emina Derviševic, Vinoth Gnana Chellaiyan Devanbu, Amol S Dhane, Bibha Dhungel, Xueting Ding, Huyen Phuc Do, Ojas Prakashbhai Doshi, Siddhartha Dutta, Lamiaa Labieb Mahmoud Ebraheim, Ebrahim Eini, Michael Ekholuenetale, Sharareh Eskandarieh, Andre Faro, Valery L Feigin, Gelana Fekadu, Ginenus Fekadu, Seyed-Mohammad Fereshtehnejad, Abdullah H Feroze, Pietro Ferrara, Nuno Ferreira, Claudio Fiorilla, Florian Fischer, Arianna Fornari, Celia Fortuna Rodrigues, Matteo Foschi, Abdelrahman Gamil Gad, Márió Gajdács, David Garcia-Azorin, Zisis Gatzioufas, Rupesh K Gautam, Miglas Welay Gebregergis, Elena V Gnedovskaya, Massimiliano Gobbo, Mahaveer Golechha, Pouya Goleij, Alessandra C Goulart, Ishita Gupta, Sapna Gupta, Roberth Steven Gutiérrez-Murillo, Najah R Hadi, Faraidoon Haghdoost, Hailey Hagins, Mohamed Hamed, Victoria Funmilayo Hanson, Amr Hassan, Simon I Hay, Khezar Hayat, Jeffrey J Hebert, Golnaz Heidari, Bartosz Helfer, Mehdi Hoseinzadeh, Md Jubayer Hossain, Yongsong Huang, Luigi Francesco Iannone, Segun Emmanuel Ibitoye, Olayinka Stephen Ilesanmi, Irena M Ilic, Muhana Fawwazy Ilyas, Salim Ilyasu, Md Rabiul Islam, Md Sahidul Islam, Nahlah Elkudssiah Ismail, Louis Jacob, Haitham Jahrami, Ammar Abdulrahman Jairoun, Navid Jamali, Manthan Dilipkumar Janodia, Ruwan Duminda Jayasinghe, Shuai Jin, Jost B Jonas, Nitin Joseph, Charity Ehimwenma Joshua, Saltanat Kamenova, Arun Kamireddy, Rami S Kantar, Sujita Kumar Kar, Mohmed Isaqali Karobari, Himanshu Khajuria, Sameer Uttamaro Khasbage, Jagdish Khubchandani, Yun Jin Kim, Omid Kohandel Gargari, Farzad Kompani, Aida Kondybayeva, Kewal Krishan, Barthelemy Kuate Defo, Mukhtar Kulimbet, Chandan Kumar, Rakesh Kumar, Vijay Kumar, Ville Kytö, Caterina Ledda, Seung Won Lee, Jacopo Lenzi, Jianan Li, Linyan Li, Yanxue Lian, Giancarlo Lucchetti, Jay B Lusk, Ricardo Lutzky Saute, Sasikumar Mahalingam, Rituparna Maiti, Ahmad Azam Malik, Birhanemaskal Malkamu, Vahid Mansouri, Konstantinos Margetis, Roy Rillera Marzo, Yasith Mathangasinghe, Georgios Mavrovounis, Hadush Negash Meles, Atte Meretoja, Tomislav Mestrovic, Sachith Mettananda, Bartosz Miazgowski, Giuseppe Minervini, Archana Mishra, Arup Kumar Misra, Khabab Abbasher Hussien Mohamed Ahmed, Omer Mohammed, Shafiu Mohammed, Ali H Mokdad, Shaher Momani, Maziar Moradi-Lakeh, Mahdis Morovvati, Reza Mosaddeghi Heris, Kavita Munjal, Yanjinlkham Munkhsaikhan, Efren Murillo-Zamora, Christopher J L Murray, Ghulam Mustafa, Fatemehzahra Naddafi, Zuhair S Natto, Gaurav Nepal, Charles Richard James Newton, Cao Duy Nguyen, Cuong Tat Nguyen, Hien Thu Nguyen, Long Nguyen, Robina Khan Niazi, Luciano Nieddu, Fred Nugen, Chijindu N Nwakama, Ogochukwu Janet Nzoputam, Bogdan Oancea, Michael Safo Oduro, Hassan Okati-Aliabad, Andrew T Olagunju, Arão Belitardo Oliveira, Jia Ouyang, Mark Overton, Mayowa O Owolabi, Mahesh P A, Giuseppina Palena, Leonidas D Panos, Ioannis Pantazopoulos, Shahina Pardhan, Romil R Parikh, Arpit Parmar, Maja Pasovic, Shankargouda Patil, Apurba Patra, Paolo Pedersini, Mario F P Peres, Simone Perna, Wajida Perveen, Hai Quang Pham, Sanjay Prakash, Akila Prashant, Jagadeesh Puvvula, Maja R Radojcic, Alberto Raggi, Mohammad Meshbahur Rahman, Amir Masoud Rahmani, Mahmoud Mohammed Ramadan, Devarajan Rathish, Salman Rawaf, Mohsen Rezaeian, Taeho Gregory Rhee, Debby Syahru Romadlon, Michele Romoli, Marina Romozzi, Umar Saeed, Amene Saghazadeh, Amirhossein Sahebkar, Mohamed A Saleh, Sohrab Salimi, Abdallah M Samy, Lucas H C C Santos, Aswini Saravanan, Hemen Sarma, Yigit Can Senol, Yashendra Sethi, Yara Khaled Fouad Sayed Shaalan, Wajeehah Shahid, Anas Shamsi, Dan Shan, Amin Sharifan, Rekha Raghuveer Shenoy, Premalatha K Shetty, Zahra Shokati Eshkiki, Sunil Shrestha, Harmanjit Singh, Jasvinder A Singh, Satwinder Singh, Valentin Yurievich Skryabin, Farrukh Sobia, Reed J D Sorensen, Sebastian Straube, Chandan Kumar Swain, Sree Sudha T Y, Payam Tabaee Damavandi, Celine Tabche, Mohsan Tanveer, Minale Tareke, Claudia Baptista Tavares, Mohamad-Hani Temsah, Masayuki Teramoto, Arun James Thirunavukarasu, Ashutosh Tiwari, Thang Huu Tran, Nguyen Tran Minh Duc, Vasilis-Spyridon Tseriotis, Santhosh Kumar Tumkur Narayanappa, Aniefiok John Udoakang, Himayat Ullah, Jibrin Sammani Usman, Abdulkadir Usman Sambo, Jef Van den Eynde, Tommi Juhani Vasankari, Narayanaswamy Venketasubramanian, Jorge Hugo Villafañe, Arvinder Wander, Wei Wang, Xingxin Wang, Yuan-Pang Wang, Taweewat Wiangkham, Mieszko Wieckiewicz, Wanqing Xu, Saba Yahoo (Syed), Yazachew Engida Yismaw, Dong Keon Yon, Naohiro Yonemoto, Abdilahi Yousuf, Aurora Zanghì, Michael Zastrozhin, Anthony Lin Zhang, Zhongyi Zhao, Magdalena Zielinska, Kanyin Liane Ong, Timothy J Steiner, Theo Vos

**Affiliations:** A Department of Neuromedicine and Movement Science, Norwegian University of Science and Technology, Trondheim, Norway; B Institute for Health Metrics and Evaluation, University of Washington, Seattle , WA, USA; C Shahid Beheshti University of Medical Sciences, Tehran, Iran; D Department of Nursing, Al Zaytoonah University of Jordan, Amman, Jordan; E Community and Maternity Nursing Unit, University of Duhok, Duhok, Iraq; F Department of Family and Community Health, University of Health and Allied Sciences, Ho, Ghana; G School of Population Health, University of New South Wales, Sydney, NSW, Australia; H Department of Anesthesiology, Shahid Beheshti University of Medical Sciences, Tehran, Iran; I Department of Neurology, Martin Luther University Halle-Wittenberg, Halle (Saale), Germany; J Department of Pharmacology and Therapeutics, United Arab Emirates University, Al Ain, United Arab Emirates; K College of Pharmacy, University of Jordan, Amman, Jordan; L Department of Public Health, Universitas Padjadjaran (Padjadjaran University), Bandung, Indonesia; M School of Arts and Social Sciences, Edith Cowan University, Bunbury, WA, Australia; N Department of Public Health and Preventive Medicine, University of Naples “Federico II”, Naples, Italy; O School of Medicine and Psychology, Australian National University, Canberra, ACT, Australia; P Health Research Institute, University of Canberra, Canberra, NSW, Australia; Q School of Medicine, Tehran University of Medical Sciences, Tehran, Iran; R Department of Pharmacy Practice, Riphah Institute of Pharmaceutical Sciences, Islamabad, Pakistan; S Division of Infectious Diseases and Global Public Health (IDGPH), University of California San Diego, San Diego, CA, USA; T Institute of Molecular Biology and Biotechnology, The University of Lahore, Lahore, Pakistan; U Department of Medicine, Fatima Memorial Hospital College of Medicine and Dentistry, Lahore, Pakistan; V School of Public Health, University of Texas, Houston, TX, USA; W School of Medicine, King Abdulaziz University, Jeddah, Saudi Arabia; X Department of Neurology and Rehabilitation Medicine, George Washington University, Arlington, VA, USA; Y Department of Rehabilitation, Montefiore Medical Center, Bronx, NY, USA; Z Department of Epidemiology, Columbia University, New York, NY, USA; AA Department of Community and Mental Health, Al al-Bayt University, Mafraq, Jordan; AB College of Nursing, Qatar University, Doha, Qatar; AC Department of Oral and Maxillofacial Surgery, Xi'an Jiaotong University, Xi'an, China; AD Department of Medical Rehabilitation (Physiotherapy), University of Maiduguri, Maiduguri, Nigeria; AE Nethersole School of Nursing, The Chinese University of Hong Kong, Hong Kong, China; AF Center for Biotechnology and Microbiology, University of Swat, Swat, Pakistan; AG The School of Medicine, The University of Jordan, Amman, Jordan; AH Department of Medicine, Nazarbayev University, Astana, Kazakhstan; AI Department of Family and Community Medicine, University of Jeddah, Jeddah, Saudi Arabia; AJ Faculty of Health Sciences, Equator University of Science and Technology, Uganda, Masaka, Uganda; AK Department of Rehabilitation Sciences, Jordan University of Science and Technology, Irbid, Jordan; AL College Of Medical Sciences, Fakeeh College for Medical Sciences, Jeddah, Saudi Arabia; AM College of Medical Sciences, Azal University for Human Development, Sana'a, Yemen; AN Department of Pharmaceutical Sciences, Qatar University, Doha, Qatar; AO Spiritual Health Research Center, Baqiyatallah University of Medical Sciences, Tehran, Iran; AP Department of Medicine, University of Jos, Jos, Nigeria; AQ Department of Internal Medicine, Jos University Teaching Hospital, Jos, Nigeria; AR Faculty of Medicine and Health, University of Sydney, Sydney, NSW, Australia; AS Sydney Musculoskeletal Health, University of Sydney, Sydney, NSW, Australia; AT Department of Pharmacology, All India Institute of Medical Sciences, Bhubaneswar, India; AU Health Management and Economics Research Center, Iran University of Medical Sciences, Tehran, Iran; AV Department of Applied Mathematics, University of Washington, Seattle, WA, USA; AW Department of Health Metrics Sciences, School of Medicine, University of Washington, Seattle, WA, United States of America; AX College of Art and Science, Ottawa University, Surprise, AZ, USA; AY School of Life Sciences, Arizona State University, Tempe, AZ, USA; AZ School of Medicine, Shahid Beheshti University of Medical Sciences, Tehran, Iran; BA College of Medicine, University of Arizona, Tucson, AZ, USA; BB Cabrini Research, Cabrini Health, Malvern, VIC, Australia; BC School of Public Health and Preventive Medicine, Monash University, Melbourne, VIC, Australia; BD Department of Anesthesia, Critical Care and Pain Medicine, Harvard University, Boston, MA, USA; BE Department of Clinical Medicine, University of Copenhagen, Copenhagen, Denmark; BF Department of Computer Engineering, King Saud University, Riyadh, Saudi Arabia; BG Research and Technology Deputy, Kurdistan University of Medical Sciences, Sanandaj, Iran; BH ASIDE Healthcare, Lewes, DE, USA; BI Faculty of Medicine, October 6 University, 6th of October City, Egypt; BJ Department of Precision Medicine, Sungkyunkwan University, Seongnam, South Korea; BK College of Optometry, Pacific University, Forest Grove, OR, USA; BL Nuffield Department of Surgical Sciences, University of Oxford, Oxford, UK; BM Department of Neurosurgery, University of Southampton, Southampton, UK; BN Clinical Sciences Department, University of Sharjah, Sharjah, United Arab Emirates; BO Health Information Management, Shiraz University of Medical Sciences, Shiraz, Iran; BP College of Medicine, Jouf University, Sakaka, Saudi Arabia; BQ Non-communicable Diseases Research Center, Tehran University of Medical Sciences, Tehran, Iran; BR School of Medicine, Iran University of Medical Sciences, Tehran, Iran; BS Department of Pediatrics, Isfahan University of Medical Sciences, Isfahan, Iran; BT Department of Pediatric Neurology, Tehran University of Medical Sciences, Tehran, Iran; BU Department of General Practice and Emergency Medicine, Karnali Academy of Health Sciences, Jumla, Nepal; BV Global Health Neurology Lab, NSW Brain Clot Bank, Sydney, NSW, Australia; BW Division of Cerebrovascular Medicine and Neurology, National Cerebral and Cardiovascular Center, Suita, Japan; BX Department of Medical Lab Technology, Chandigarh University, Mohali, India; BY Laboratory of Translational Medicine and Nanotherapeutics, Central University of Punjab, Bathinda, India; BZ Department of Pharmaceutical Sciences, Guru Nanak Dev University, Amritsar, India; CA Department of Community and Family Medicine, All India Institute of Medical Sciences, Deoghar, India; CB Department of Neurobiology, Care Sciences and Society, Karolinska Institute, Stockholm, Sweden; CC Department of Physical Education and Health, Universidad de la República, Rivera, Uruguay; CD Department of Internal Medicine, Manipal Academy of Higher Education, Mangalore, India; CE Faculty of Medicine, Mohammed VI University of Health Science, Casablanca, WA, Morocco; CF School of Pharmacy, The University of Jordan, Amman, Jordan; CG Department of Basic Biomedical Sciences, University of Sharjah, Sharjah, United Arab Emirates; CH JSS Dental College & Hospital, Jagadguru Sri Shivarathreeswara University, Mysore, India; CI Department of Internal and Geriatric Medicine, Hospital Italiano de Buenos Aires (Italian Hospital of Buenos Aires), Buenos Aires, Argentina; CJ Board of Directors, Argentine Society of Medicine, Buenos Aires, Argentina; CK Headache and Neurological Pain Research Group, Vall d'Hebron Research Institute (VHIR), Barcelona, Spain; CL School of Health Science, University of Sydney, Sydney, NSW, Australia; CM Education Center of Australia, Health Science College, Sydney, NSW, Australia; CN State Disease Investigation Laboratory, Animal Resources Development Department, Agartala, India; CO Department of Epidemiology and Biostatistics, Semey Medical University (SMU), Semey, Kazakhstan; CP Department of Community Medicine, Datta Meghe Institute of Medical Sciences, Sawangi, India; CQ Department of Biology, Imam Mohammad Ibn Saud Islamic University, Riyadh, Saudi Arabia; CR Division of Infectious Diseases, Virginia Commonwealth University, Richmond, VA, USA; CS Department of Pediatrics, Peking University, Beijing, China; CT Department of Clinical Medicine, University of Bergen, Bergen, Norway; CU NorHEAD Norwegian Center for Headache Research, Norwegian University of Science and Technology, Trondheim, Norway; CV Life and Health Sciences Research Institute (ICVS), University of Minho, Braga, Portugal; CW Institute for Research and Innovation in Health (i3S), University of Porto, Porto, Portugal; CX Department of Health, Northern Territory Government, Darwin, WA, Australia; CY Department of Health Metrics Sciences, School of Medicine, University of Washington, Seattle, WA, USA; CZ Department of Medical and Surgical Sciences and Advanced Technologies “GF Ingrassia”, University of Catania, Catania, Italy; DA Institute for Global Health Innovations, Duy Tan University, Hanoi, Vietnam; DB Department of Brain Sciences, Imperial College London, London, UK; DC Department of Pediatrics, Brookdale University Hospital Medical Center, Brooklyn, NY, USA; DD Department of Neurosurgery, Tehran University of Medical Sciences, Tehran, Iran; DE Department of Neurosurgery, University of Edinburgh, Edinburgh, UK; DF Department of Neurosurgery, National Health Service (NHS) Scotland, Edinburgh, UK; DG Department of Forensic Medicine, University of Sarajevo, Sarajevo, Bosnia and Herzegovina; DH Chettinad Hospital & Research Institute, Chettinad Academy of Research and Education, Chennai, India; DI Research and Development Cell, Dr. D. Y. Patil Vidyapeeth, Pune (Deemed to be University), Pune, India; DJ Population Interventions Unit, University of Melbourne, Melbourne, VIC, Australia; DK Joe C. Wen School of Population & Public Health, University of California Irvine, Irvine, CA, USA; DL College of Health Sciences, VinUniversity, Hanoi, Vietnam; DM Institute of Health Economics and Technology (iHEAT), Hanoi, Vietnam; DN Independent Consultant, Bridgewater, NJ, USA; DO Department of Pharmacology, All India Institute of Medical Sciences, Rajkot, India; DP Histology Department, Zagazig University, Zagazig, Egypt; DQ Independent Consultant, Ahvaz, Iran; DR Faculty of Science and Health, University of Portsmouth, Hampshire, UK; DS Multiple Sclerosis Research Center, Tehran University of Medical Sciences, Tehran, Iran; DT Department of Psychology, Federal University of Sergipe, São Cristóvão, Brazil; DU National Institute for Stroke and Applied Neurosciences, Auckland University of Technology, Auckland, New Zealand; DV Research Center of Neurology, Moscow, Russia; DW School of Nursing, Haramaya University, Harar, Ethiopia; DX Department of Infectious Diseases and Public Health, City University of Hong Kong, Hong Kong, China; DY Department of Pharmacy, Wollega University, Nekemte, Ethiopia; DZ Division of Neurology, University of Toronto, Toronto, ON, Canada; EA Department of Neurobiology, Care Sciences, and Society, Karolinska Institute, Stockholm, Sweden; EB Department of Neurosurgery, Children's National Medical Center, Washington, DC, USA; EC Department of Neurosurgery, George Washington University, Washington, DC, USA; ED Center for Public Health Research, University of Milan Bicocca, Monza, Italy; EE Laboratory of Public Health, IRCCS Istituto Auxologico Italiano, Milan, Italy; EF Department of Social Sciences, University of Nicosia, Nicosia, Cyprus; EG Department of Public Health, University of Naples “Federico II”, Naples, Italy; EH Institute of Public Health, Charité Universitätsmedizin Berlin (Charité Medical University Berlin), Berlin, Germany; EI UO Neurologia, Salute Pubblica e Disabilità (The Neurology, Public Health and Disability Unit), Fondazione IRCCS Istituto Neurologico Carlo Besta (IRCCS Foundation Carlo Besta Neurological Institute), Milan, Italy; EJ Applied Molecular Biosciences Unit, University of Porto, Porto, Portugal; EK Faculty of Engineering, University of Porto, Porto, Portugal; EL Department of Neuroscience, Multiple Sclerosis Research Center, Ravenna, Italy; EM Department of Biotechnological and Applied Clinical Sciences, University of L'Aquila, L'Aquila, Italy; EN Russell H. Morgan Department of Radiology and Radiological Science, Johns Hopkins University, Baltimore, MD, USA; EO Department of Public Health, University of Szeged, Szeged, Hungary; EP Department of Medicine, University of Valladolid, Valladolid, Spain; EQ Department of Neurology, Hospital Universitario Rio Hortega, Valladolid, Spain; ER Department of Ophthalmology, University of Basel, Basel, Switzerland; ES Centre for Pharmacology, Amity Institute of Pharmacy, Noida, India; ET Department of Midwifery, Adigrat University, Adigrat, Ethiopia; EU Third Department of Neurology, Research Center of Neurology, Moscow, Russia; EV Department of Clinical and Experimental Sciences, University of Brescia, Brescia, Italy; EW IRCSS Fondazione Don Gnocchi, IRCCS Fondazione Don Gnocchi, Rovato (Brescia), Italy; EX Department of Health Systems and Policy Research, Indian Institute of Public Health, Gandhinagar, India; EY Department of Genetics, Sana Institute of Higher Education, Sari, Iran; EZ Universal Scientific Education and Research Network (USERN), Kermanshah University of Medical Sciences, Kermanshah, Iran; FA Department of Epidemiology, Universidade de São Paulo (University of São Paulo), São Paulo, Brazil; FB Department of Internal Medicine, Independent Consultant, Bharatpur, India; FC Independent Consultant, Delhi, India; FD Department of Toxicology, Shriram Institute for Industrial Research, Delhi, India; FE Doctoral Program in Biomedical Gerontology, Pontifical Catholic University of Rio Grande do Sul, Porto Alegre, Brazil; FF Department of Clinical Pharmacology and Medicine, University of Kufa, Najaf, Iraq; FG The George Institute for Global health, University of New South Wales, Sydney, NSW, Australia; FH Oral & Maxillofacial Rehabilitation Department, King Abdulaziz University, Jeddah, Saudi Arabia; FI Department of Fixed Prosthodontics, Cairo University, Cairo, Egypt; FJ Rak Medical and Health Sciences University, Rak Medical and Health Sciences University, Ras Al-Khaimah, United Arab Emirates; FK Department of Neurology, Cairo University, Cairo, Egypt; FL Institute of Pharmaceutical Sciences, University of Veterinary and Animal Sciences, Lahore, Pakistan; FM Department of Pharmacy Administration and Clinical Pharmacy, Xian Jiaotong University, Xian, China; FN Faculty of Kinesiology, University of New Brunswick, Fredericton, NB, Canada; FO School of Allied Health, Murdoch University, Murdoch, WA, Australia; FP Independent Consultant, Santa Clara, CA, USA; FQ Institute of Psychology, University of Wroclaw, Wroclaw, Poland; FR Meta Research Centre, University of Wroclaw, Wroclaw, Poland; FS School of Computer Science, Duy Tan University, Da Nang, Vietnam; FT Department of AI, Galgotias University, Greater Noida, India; FU Center for Health Innovation, Research, Action and Learning - Bangladesh (CHIRAL Bangladesh), Dhaka, Bangladesh; FV Department of Public Health, Daffodil International University, Dhaka, Bangladesh; FW Advanced Institute of Convergence Knowledge Informatics, Tohoku University, Sendai, Japan; FX Graduate School of Engineering, Tohoku University, Sendai, Japan; FY Department of Biomedical, Metabolic, and Neural Science, University of Modena and Reggio Emilia, Modena, Italy; FZ Department of Health Promotion and Education, University of Ibadan, Ibadan, Nigeria; GA West Africa RCC, Africa Centre for Disease Control and Prevention, Abuja, Nigeria; GB Department of Community Medicine, University College Hospital, Ibadan, Ibadan, Nigeria; GC Faculty of Medicine, University of Belgrade, Belgrade, Serbia; GD Department of Neurosurgery, Universitas Sebelas Maret (March Eleventh University), Jakarta, Indonesia; GE Department of Pharmaceutics and Pharmaceutical Technology, Bayero University, Kano, Nigeria; GF School of Pharmacy, BRAC University, Dhaka, Bangladesh; GG Research and Publication Department, World Health Organization (WHO), Dhaka, Bangladesh; GH Department of Clinical Pharmacy & Pharmacy Practice, Asian Institute of Medicine, Science and Technology, Bedong, Malaysia; GI Malaysian Academy of Pharmacy, Puchong, Malaysia; GJ Department of Physical Medicine and Rehabilitation, Université Paris Cité, Paris, France; GK Research and Development Unit, Biomedical Research Networking Center for Mental Health Network (CiberSAM), Barcelona, Spain; GL College of Medicine and Health Sciences, Arabian Gulf University, Manama, Bahrain; GM Government Hospitals, Manama, Bahrain; GN Department of Health and Safety, Dubai Municipality, Dubai, United Arab Emirates; GO Department of Laboratory Sciences, Sirjan School of Medical Sciences, Sirjan, Iran; GP Malla Reddy Vishwavidyapeeth, Hyderabad, India; GQ Sri Devraj Urs Academy of Higher Education and Research, Kolar, India; GR Department of Oral Medicine and Periodontology, University of Peradeniya, Peradeniya, Sri Lanka; GS Department of Oral Medicine and Periodontology, Saveetha University, Chennai, India; GT School of Biology and Engineering, Guizhou Medical University, Guiyang, China; GU Rothschild Foundation Hospital, Institut Français de Myopie, Paris, France; GV Singapore Eye Research Institute, Singapore Eye Research Institute, Singapore, Singapore; GW Department of Community Medicine, Manipal Academy of Higher Education, Mangalore, India; GX Department of Economics, National Open University, Benin City, Nigeria; GY Department of General Medical Practice No. 2, Kazakh National Medical University, Almaty, Kazakhstan; GZ The Hansjörg Wyss Department of Plastic and Reconstructive Surgery, NYU Langone Health, New York, NY, USA; HA Cleft Lip and Palate Surgery Division, Global Smile Foundation, Norwood, MA, USA; HB Department of Psychiatry, King George's Medical University, Lucknow, India; HC Saveetha Medical College and Hospital, Saveetha University, Chennai, India; HD Amity Institute of Forensic Sciences, Amity University, Noida, India; HE Department of Pharmacology, All India Institute of Medical Sciences, Raipur, India; HF Department of Public Health, New Mexico State University, Las Cruces, NM, USA; HG School of Traditional Chinese Medicine, Xiamen University Malaysia, Sepang, Malaysia; HH Neurology Department, Tehran University of Medical Sciences, Tehran, Iran; HI Department of Medicine, Alborz University of Medical Sciences, Karaj, Iran; HJ Children's Medical Center, Tehran University of Medical Sciences, Tehran, Iran; HK Scientific and Educational Center for Neurology and Applied Neuroscience, Kazakh National Medical University, Almaty, Kazakhstan; HL Department of Anthropology, Panjab University, Chandigarh, India; HM Department of Demography, University of Montreal, Montreal, QC, Canada; HN Department of Social and Preventive Medicine, University of Montreal, Montreal, QC, Canada; HO Research and Publication Activity Division, Kazakh National Medical University, Almaty, Kazakhstan; HP Center of Medicine and Public Health, Asfendiyarov Kazakh National Medical University, Almaty, Kazakhstan; HQ Amity Institute of Health Allied Sciences, Amity University Uttar Pradesh, Noida, India; HR Department of Health Management, University of Hail, Hail, Saudi Arabia; HS Department of Economics, Manipal University, Jaipur, India; HT Clinical Research Center, Turku University Hospital, Turku, Finland; HU Heart Center, University of Turku and Turku University Hospital, Turku, Finland; HV Department of Clinical and Experimental Medicine, University of Catania, Catania, Italy; HW Department of Precision Medicine, Sungkyunkwan University, Suwon-si, South Korea; HX Department of Biomedical and Neuromotor Sciences, University of Bologna, Bologna, Italy; HY School of Public Health, Xuzhou medical university, Xuzhou, China; HZ Discipline of Physiology, National University of Ireland - Galway, Galway, Ireland; IA School of Medicine, Federal University of Juiz de Fora, Juiz de Fora, Brazil; IB Department of Population Health Sciences, Duke University, Durham, NC, USA; IC Department of Family Medicine, University of North Carolina Chapel Hill, Chapel Hill, NC, USA; ID Department of Neurosciences and Behavioral Sciences, University of São Paulo, Ribeirão Preto, Brazil; IE Department of Emergency Medicine, Sri Lakshmi Narayana Institute of Medical Science, Puducherry, Pondicherry, India; IF Rabigh Faculty of Medicine, King Abdulaziz University, Jeddah, Saudi Arabia; IG College of Health Sciences, Debre Tabor University, Debre Tabor, Ethiopia; IH Digestive Diseases Research Institute, Tehran University of Medical Sciences, Tehran, Iran; II Department of Neurosurgery, Icahn School of Medicine at Mount Sinai, New York, NY, USA; IJ Faculty of Humanities and Health Sciences, Curtin University, Sarawak, Malaysia; IK Jeffrey Cheah School of Medicine and Health Sciences, Monash University, Subang Jaya, Malaysia; IL Department of Anatomy and Developmental Biology, Monash University, Clayton, VIC, Australia; IM Department of Anatomy, Genetics and Biomedical Informatics, University of Colombo, Colombo, Sri Lanka; IN Department of Emergency Medicine, University of Thessaly, Larissa, Greece; IO Department of Medical Laboratory Sciences, Adigrat University, Adigrat, Ethiopia; IP General Administration Department, Helsinki University Hospital, Helsinki, Finland; IQ School of Health Sciences, University of Melbourne, Melbourne, VIC, Australia; IR University Centre Varazdin, University North, Varazdin, Croatia; IS Department of Paediatrics, University of Kelaniya, Ragama, Sri Lanka; IT University Paediatrics Unit, Colombo North Teaching Hospital, Ragama, Sri Lanka; IU Clinical Emergency Department, Pomeranian Medical University, Szczecin, Poland; IV Pomeranian Medical University, Szczecin, Poland; IW Multidisciplinary Department of Medical-Surgical and Dental Specialties, University of Campania Luigi Vanvitelli, Naples, Italy; IX Saveetha Dental College and Hospitals, Saveetha University, Chennai, India; IY Department of Pharmacology, All India Institute of Medical Sciences, Mangalagiri, India; IZ Faculty of Medicine, University of Khartoum, Khartoum, Sudan; JA Department of Medicine, Government Medical College Kozhikode, Kozhikode, India; JB Health Systems and Policy Research Unit, Ahmadu Bello University, Zaria, Nigeria; JC Heidelberg Institute of Global Health (HIGH), Heidelberg University, Heidelberg, Germany; JD Department of Mathematics, The University of Jordan, Amman, Jordan; JE Nonlinear Dynamics Research Center (NDRC), Ajman University, Ajman, United Arab Emirates; JF Gastrointestinal and Liver Diseases Research Center, Iran University of Medical Sciences, Tehran, Iran; JG Preventive Medicine and Public Health Research Center, Iran University of Medical Sciences, Tehran, Iran; JH Baan Clinic, Tehran, Iran; JI Neurosciences Research Center (NSRC), Tabriz University of Medical Sciences, Tabriz, Iran; JJ Student Research Committee, Tabriz University of Medical Sciences, Tabriz, Iran; JK Amity Institute of Pharmacy, Amity University, Noida, India; JL Department of Community and Global Health, The University of Tokyo, Tokyo, Japan; JM Clinical Epidemiology Research Unit, Mexican Institute of Social Security, Villa de Alvarez, Mexico; JN Postgraduate in Medical Sciences, Universidad de Colima, Colima, Mexico; JO College of Medicine, Shaqra University, Shaqra, Saudi Arabia; JP Department of Pediatrics & Pediatric Pulmonology, Institute of Mother & Child Care, Multan, Pakistan; JQ Department of Geriatric Health, Tabriz University of Medical Sciences, Tabriz, Iran; JR Department of Health Education & Promotion, Gonabad University of Medical Sciences, Gonabad, Iran; JS Department of Dental Public Health, King Abdulaziz University, Jeddah, Saudi Arabia; JT Department of Neurology, Case Western Reserve University, Cleveland, OH, USA; JU Department of Psychiatry, University of Oxford, Oxford, UK; JV Department of Neurosciences, Kenya Medical Research Institute/Wellcome Trust Research Programme, Kilifi, Kenya; JW Institute for Global Health Innovations, Duy Tan University, Da Nang, Vietnam; JX Faculty of Public Health, VNU University of Medicine and Pharmacy, Hanoi, Vietnam; JY International Institute for Training and Research (INSTAR), VNU University of Medicine and Pharmacy, Hanoi, Vietnam; JZ International Islamic University Islamabad, Islamabad, Pakistan; KA Department of Humanities and Social Science, University for International Studies in Rome, Rome, Italy; KB Department of Radiology, Mayo Clinic, Rochester, MN, USA; KC School of Information, University of California Berkeley, Berkeley, CA, USA; KD School of Medicine, Johns Hopkins University, Baltimore, MD, USA; KE Department of Physiology, University of Benin, Edo, Nigeria; KF Department of Physiology, Benson Idahosa University, Benin City, Nigeria; KG Department of Applied Economics and Quantitative Analysis, University of Bucharest, Bucharest, Romania; KH Bioinformatics Department, National Institute of Research and Development for Biological Sciences, Bucharest, Romania; KI Pfizer Research & Development, Pfizer Inc., Groton, CT, USA; KJ Health Promotion Research Center, Zahedan University of Medical Sciences, Zahedan, Iran; KK Department of Psychiatry and Behavioural Neurosciences, McMaster University, Hamilton, ON, Canada; KL Department of Psychiatry, University of Lagos, Lagos, Nigeria; KM Center for Clinical and Epidemiological Research, University of São Paulo, São Paulo, Brazil; KN Associação Brasileira de Cefaleia em Salvas e Enxaqueca (ABRACES), São Paulo, Brazil; KO Peking University People's Hospital, Peking University, Beijing, China; KP Graduate School of Health, University of Technology Sydney, Sydney, NSW, Australia; KQ Department of Medicine, University of Ibadan, Ibadan, Nigeria; KR Department of Medicine, University College Hospital, Ibadan, Ibadan, Nigeria; KS Department of Respiratory Medicine, Jagadguru Sri Shivarathreeswara University, Mysore, India; KT Department of Public Health, University “Federico II” of Naples, Naples, Italy; KU Department of Neurology, University of Bern, Biel/Biene, Switzerland; KV Department of Neurology, University of Cyprus, Nicosia, Cyprus; KW Department of Emergency Medicine, University of Bern, Bern, Switzerland; KX Vision and Eye Research Institute, Anglia Ruskin University, Cambridge, UK; KY Division of Health Policy and Management, University of Minnesota, Minneapolis, MN, USA; KZ Department of Psychiatry, All India Institute of Medical Sciences, Bhubaneswar, India; LA College of Dental Medicine, Roseman University of Health Sciences, South Jordan, UT, USA; LB Department of Human Anatomy, All India Institute of Medical Sciences, Bathinda, India; LC IRCCS Fondazione Don Carlo Gnocchi, Milan, Italy; LD Department of Psychiatry, University of São Paulo, São Paulo, Brazil; LE International Institute for Educational Planning (IIEP), Albert Einstein Hospital, São Paulo, Brazil; LF Department of Food, Environmental and Nutritional Sciences, University of Milan, Milan, Italy; LG CMH Lahore Medical College, CMH Lahore Medical College & Institute of Dentistry, Lahore, Pakistan; LH Center of Excellence in Behavioral Medicine, Nguyen Tat Thanh University, Ho Chi Minh City, Vietnam; LI Department of Neurology, Smt. B.K.S. Medical Institute and Research Center, Vadodara, India; LJ Department of Biochemistry, JSS Academy of Higher Education and Research, Mysuru, India; LK Department of Biostatistics, Epidemiology, and Informatics, University of Pennsylvania, Philadelphia, PA, USA; LL Division of Psychology and Mental Health, University of Manchester, Manchester, UK; LM Department of Biostatistics, National Institute of Preventive and Social Medicine, Dhaka, Bangladesh; LN Future Technology Research Center, National Yunlin University of Science and Technology, Yunlin, Taiwan; LO Department of Clinical Sciences, University of Sharjah, Sharjah, United Arab Emirates; LP Department of Cardiology, Mansoura University, Mansoura, Egypt; LQ Department of Family Medicine, Rajarata University of Sri Lanka, Anuradhapura, Sri Lanka; LR Department of Primary Care and Public Health, Imperial College London, London, UK; LS Academic Public Health England, Public Health England, London, UK; LT Department of Epidemiology and Biostatistics, Rafsanjan University of Medical Sciences, Rafsanjan, Iran; LU Department of Public Health Sciences, University of Connecticut, Farmington, CT, USA; LV Department of Psychiatry, Yale University, New Haven, CT, USA; LW Faculty of Nursing, Chulalongkorn University, Bangkok, Thailand; LX Department of Neurosciences, Maurizio Bufalini Hospital, Cesena, Italy; LY Centre for Global Epilepsy, University of Oxford, Oxford, UK; LZ Fondazione Policlinico Universitario A. Gemelli, Cuore Università Cattolica del Sacro Cuore (Catholic University of Sacred Heart), Rome, Italy; MA Széchenyi István University, Gyor, Hungary; MB Operational Research Center in Healthcare, Near East University, Cyprus, Turkiye; MC Research Center for Immunodeficiencies, Tehran University of Medical Sciences, Tehran, Iran; MD Center for Global Health Research, Saveetha University, Chennai, India; ME Biotechnology Research Center, Mashhad University of Medical Sciences, Mashhad, Iran; MF College of Medicine, University of Sharjah, Sharjah, United Arab Emirates; MG Faculty of Pharmacy, Mansoura University, Mansoura, Egypt; MH Department of Entomology, Ain Shams University, Cairo, Egypt; MI Medical Ain Shams Research Institute (MASRI), Ain Shams University, Cairo, Egypt; MJ University of São Paulo City, São Paulo, Brazil; MK Department of Pharmacology, All India Institute of Medical Sciences, Jodhpur, India; ML Indira Gandhi Medical College and Research Institute, Puducherry, India; MM Botany Department, Bodoland University, Kokrajhar, India; MN Department of Neurosurgery, University of California San Francisco, San Francisco, CA, USA; MO Department of Medicine, Swami Vivekanand Subharti University, Meerut, India; MP Faculty of Medicine, Misr University for Science and Technology, 6th of October city, Egypt; MQ Department of Physics, The University of Lahore, Lahore, Pakistan; MR Center for Medical and Bio-Allied Health Sciences Research, Ajman University, Ajman, United Arab Emirates; MS Centre For Interdisciplinary Research In Basic Sciences (CIRBSc), Jamia Millia Islamia, New Delhi, India; MT Lancaster University, Lancaster, UK; MU Columbia University, New York, NY, USA; MV Department for Evidence-based Medicine and Evaluation, University for Continuing Education Krems, Krems, Austria; MW Department of Pharmacology, Manipal Academy of Higher Education, Manipal, India; MX Manipal College of Dental Sciences, Mangalore, Manipal Academy of Higher Education, Manipal, India; MY Alimentary Tract Research Center, Ahvaz Jundishapur University of Medical Sciences, Ahvaz, Iran; MZ Department of Research and Academics, Kathmandu Cancer Center, Bhaktapur, Nepal; NA Person-Centered Research, Monash University, Box Hill, VIC, Australia; NB Department of Pharmacology, Government Medical College and Hospital, Chandigarh, India; NC School of Medicine, Baylor College of Medicine, Houston, TX, USA; ND Department of Medicine Service, US Department of Veterans Affairs (VA), Houston, TX, USA; NE Department of Computer Science & Engineering, Central University of Punjab, Bathinda, India; NF Books Committee, Royal College of Psychiatrists, London, UK; NG Department of Public Health, Jazan University, Jazan, Saudi Arabia; NH Department of Global Health, University of Washington, Seattle, WA, USA; NI Division of Preventive Medicine, University of Alberta, Edmonton, AB, Canada; NJ School of Public Health, University of Alberta, Edmonton, AB, Canada; NK Department of Analytical and Applied Economics, Utkal University, Bhubaneswar, India; NL Department of Pharmacology, All India Institute of Medical Sciences, Deoghar, India; NM Department of Neurology, Neurocenter of Southern Switzerland (NSI), Lugano, Switzerland; NN Department of Computer and Software Engineering, National University of Science and Technology (NUST), Islamabad, Pakistan; NO Department of Psychiatry, Bahir Dar University, Bahir Dar, Ethiopia; NP School of Medicine at the Federal University of Minas Gerais (UFMG), Federal University of Minas Gerais, Belo Horizonte, Brazil; NQ Department of Clinical Medicine of the Fluminense Federal University, Fluminense Federal University, Rio de Janeiro, Brazil; NR Pediatric Intensive Care Unit, King Saud University, Riyadh, Saudi Arabia; NS College of Medicine, Alfaisal University, Riyadh, Saudi Arabia; NT Department of Preventive Medicine, Northwestern University, Chicago, IL, USA; NU International Centre for Eye Health, London School of Hygiene & Tropical Medicine, London, UK; NV Department of Neurology, All India Institute of Medical Sciences, Gorakhpur, India; NW Department of Internal Medicine, University of Medicine and Pharmacy at Ho Chi Minh City, Ho Chi Minh City, Vietnam; NX Department of Business Analytics, University of Massachusetts Dartmouth, Dartmouth, MA, USA; NY Research and Advocacy Initiative, ALS Vietnam, Quang Ngai, Vietnam; NZ Laboratory of Clinical Pharmacology, Aristotle University of Thessaloniki, Thessaloniki, Greece; OA Department of Epidemiology and Public Health, University College London, London, UK; OB ESIC Medical College PGIMSR and Model Hospital, Bengaluru, India; OC ESIC Alumni Association, Bengaluru, India; OD Department of Biosciences and Biotechnology, University of Medical Sciences, Ondo, Ondo, Nigeria; OE Hayatabad Medical Complex, Postgraduate Medical Institute, Peshawar, Pakistan; OF Department of Physiotherapy, Bayero University, Kano, Nigeria; OG Department of Rehabilitation Sciences, Hong Kong Polytechnic University, Hong Kong, China; OH Department of Psychiatry, Federal Neuropsychiatric Hospital, Kaduna, Nigeria; OI Department of Cardiovascular Sciences, Katholieke Universiteit Leuven, Leuven, Belgium; OJ UKK Institute, Tampere, Finland; OK Faculty of Medicine and Health Technology, Tampere University, Tampere, Finland; OL Raffles Neuroscience Centre, Raffles Hospital, Singapore, Singapore; OM Yong Loo Lin School of Medicine, National University of Singapore, Singapore, Singapore; ON Department of Physiotherapy, Universidad Europea de Madrid (European University of Madrid), Villaviciosa de Odón, Spain; OO Department of Paediatrics, All India Institute of Medical Sciences, Bathinda, India; OP School of Public Health, Xuzhou Medical University, Xuzhou, China; OQ Shandong University of Traditional Chinese Medicine, Shandong University of Traditional Chinese Medicine, Jinan, China; OR Department of Physical Therapy, Naresuan University, Phitsanulok, Thailand; OS Department of Experimental Dentistry, Wroclaw Medical University, Wroclaw, Poland; OT Department of Nutrition, Tufts University, Boston, MA, USA; OU Department of Social and Behavioral Sciences, Harvard University, Boston, MA, USA; OV Department of Community Medicine, Apollo Institute of Medical Sciences and Research, Hyderabad, India; OW Department of Pharmacology, Bahir Dar University, Bahir Dar, Ethiopia; OX Pharmacy Department, Alkan Health Science, Business and Technology College, Bahir Dar, Ethiopia; OY Department of Pediatrics, Kyung Hee University, Seoul, South Korea; OZ Department of Biostatistics, University of Toyama, Toyama, Japan; PA Department of Public Health, Juntendo University, Tokyo, Japan; PB Department of Public Health, Jigjiga University, Jigjiga, Ethiopia; PC Sant'Elia Hospital, University of Catania, Caltanissetta, Italy; PD Department of Bioengineering and Therapeutical Sciences, University of California San Francisco, San Francisco, CA, USA; PE Department of Administration, PGxAI, San Francisco, CA, USA; PF School of Health and Biomedical Sciences, Royal Melbourne Institute of Technology (RMIT) University, Melbourne, VIC, Australia; PG Department of Health Management, Shengjing Hospital of China Medical University, Shenyang, China; PH Department of Biochemistry and Pharmacogenomics, Medical University of Warsaw, Warsaw, Poland; PI Department of Neurology, University of Copenhagen, Copenhagen, Denmark

## Abstract

**Background:**

The Global Burden of Diseases, Injuries, and Risk Factors Study (GBD) 2023 estimates health loss from migraine, tension-type headache, and medication-overuse headache. This study presents updated results on headache-attributed burden from 1990 to 2023, along with clinical and public health implications.

**Methods:**

Data on the prevalence, incidence, or remission of migraine, tension-type headache, and medication-overuse headache were extracted from published population-based studies. We used hierarchical Bayesian meta-regression modelling to estimate global, regional, and country-level prevalence of headache disorders. For the first time in GBD 2023, age-specific and sex-specific estimates of time in symptomatic state were applied by meta-analysing individual participant data from 41 653 individuals from the general populations of 18 countries from all parts of the world. Disability weights were applied to calculate years lived with disability (YLDs). Since medication-overuse headache is a sequela of a mistreated primary headache (due to medication overuse), its burden was reattributed to migraine or tension-type headache, informed by a meta-analysis of three longitudinal studies.

**Findings:**

In 2023, 2·9 billion individuals (95% uncertainty interval 2·6–3·1) were affected by headache disorders, with a global age-standardised prevalence of 34·6% (31·6–37·5) and a YLD rate of 541·9 (373·4–739·9) per 100 000 population, with 487·5 (323·0–678·8) per 100 000 population attributed to migraine. The prevalence rates of these headache disorders have remained stable over the past three decades. YLD rates due to headache disorders were more than twice as high in females (739·9 [511·2–1011·5] per 100 000) as in males (346·1 [240·4–481·8] per 100 000). Medication-overuse headache contributed 58·9% of the YLD estimates for tension-type headache in males and 56·1% in females, as well as 22·6% of the YLD estimates for migraines in males and 14·1% in females.

**Interpretation:**

Headache disorders, in particular migraine, continue to be a major global health challenge, emphasising the need for effective management and prevention strategies. Much headache-attributed burden could be averted or eliminated by avoiding overuse of medication (including over-the-counter medication), underscoring the importance of public education.

**Funding:**

Gates Foundation.

## Introduction

Headache disorders are among the most prevalent disorders worldwide.^1^, ^2^, ^3^ In the past two decades, they have also been recognised as being among the leading causes of health loss.^2^, ^3^, ^4^ Currently, the Global Burden of Diseases, Injuries, and Risk Factors Study (GBD) produces estimates for migraine, tension-type headache, and medication-overuse headache—the most common headache types with greatest impacts on population health.^5^

Since there are no diagnostic biomarkers, the diagnoses of these three headache types are based on clinical criteria described in the International Classification of Headache Disorders (ICHD;^6^ panel). The reliance on self-reported symptoms is diagnostically challenging and highly dependent on the doctor–patient interaction; in epidemiological enquiry, with no such interaction, the challenges are greater, and uncertainties inevitable. Migraine and tension-type headache are identified through the presence or absence, respectively, of the same set of clinical characteristics, with migraine almost invariably being more bothersome than tension-type headache (eg, experiencing nausea, an associated symptom of migraine but not of tension-type headache, is more bothersome than not experiencing nausea). A definite diagnosis of migraine or tension-type headache is made when all criteria A–E for the diagnosis are fulfilled. Notably, the ICHD allows for a probable diagnosis when all but one of criteria A–D are fulfilled.^6^ In a clinical setting, a probable diagnosis is often later confirmed as definite.

Panel **Diagnostic criteria for migraine, tension-type headache, and medication-overuse headache according to the International Classification of Headache Disorders 3rd Edition (ICHD-3)6**

**Migraine**

(A) At least five attacks fulfilling criteria B–D
(B) Lasting 4–72 h
(C) At least two of the following four characteristics:
  (1) unilateral location
  (2) pulsating quality
  (3) moderate or severe pain intensity
  (4) aggravation by or causing avoidance of routine physical activity
(D) At least one of the following:
  (1) nausea and/or vomiting
  (2) photophobia and phonophobia
(E) Not better accounted for by another ICHD-3 diagnosis

**Tension-type headache**

(A) At least ten attacks fulfilling criteria B–D
(B) Lasting 30 min to 7 days
(C) At least two of the following four characteristics:
  (1) bilateral location
  (2) pressing (non-pulsating) quality
  (3) mild or moderate pain intensity
  (4) not aggravated by routine physical activity
(D) Both of the following:
  (1) no nausea or vomiting
  (2) no more than one of photophobia or phonophobia
(E) Not better accounted for by another ICHD-3 diagnosis

**Medication-overuse headache**

(A) Headache occurring on ≥15 days per month in a patient with a pre-existing headache disorder
(B) Regular overuse for >3 months of one or more drugs that can be taken for acute and/or symptomatic treatment of headache
(C) Not better accounted for by another ICHD-3 diagnosis

Unlike migraine and tension-type headache, medication-overuse headache does not develop de novo, but is a sequela of a primary headache (in virtually all cases, migraine or tension-type headache)^7^ brought on by over-frequent consumption of acute medication. By definition, medication-overuse headache is present on at least 15 days per month.^6^

**Research in context**

**Evidence before this study**

We searched PubMed to identify studies addressing global headache burden between Jan 1, 1990, and Jan 1, 2025, using the following search string: (headache[Title/Abstract]) AND (burden[Title/Abstract] OR health loss[Title/Abstract] OR disability[Title/Abstract]) AND ((global[Title/Abstract]) OR (international[Title/Abstract])). We found that studies mainly looked at small geographical areas (commonly single countries), focused solely on prevalence without any attempt to address health loss, or used results from the Global Burden of Disease, Injuries, and Risk Factors Study (GBD). The Global Campaign against Headache is one effort that stands out from this, consistently gathering data on prevalence and headache-attributed burden across the world. This campaign has resulted in numerous country-specific publications on the burden of headache disorders, which has informed GBD's headache models. Since migraine was included in GBD in 2000, headache disorders have been ranked among the top causes of health loss. Later, estimates for tension-type headache and medication-overuse headache have been added, the latter as a sequela of mistreated migraine or tension-type headache. Health loss has been especially high among young adults and females owing to the high prevalence of migraine. Evidence exists that females have longer headache episodes than males, but previous GBD rounds have not accounted for this.

**Added value of this study**

This study provides improved insight into the burden of headache disorders by incorporating more granular estimates of time in symptomatic state for migraine and tension-type headache in GBD. This was achieved by analysing a large amount of population-representative individual participant data, allowing separate estimates by age and sex. Compared with previous GBD rounds, this approach reduced the uncertainty of the health loss estimates and revealed greater sex differences, health loss among females (739·9 years lived with disability [YLDs] per 100 000 population) being more than double the health loss among males (346·1 YLDs per 100 000). This study also indicated that these health losses would be greatly reduced by avoiding acute medication overconsumption, since more than 50% of health loss attributed to tension-type headache and more than 15% attributed to migraine (>20% to all headache) was actually due to medication-overuse headache.

**Implications of all the available evidence**

Headache disorders continue to be among the most prevalent disorders globally and migraine among the most disabling. Females have more than twice the burden of males, owing not only to a higher prevalence of migraine but also to the fact that, at an individual level, they spend more time with headache. Improved coverage of effective headache medications (including preventive treatments) is needed, but this must be done in tandem with education on the correct use of acute medications to avoid the increased burden associated with their overconsumption.

The health loss caused by headache disorders is especially high among young adults and females, in both cases owing to a high prevalence of migraine. Previous iterations of GBD have, however, not accounted for the fact that females have longer-duration attacks than males.^8^ This study presents novel methods implemented in GBD 2023 to account for age and sex differences in time spent with headache, and uses these to present updated estimates of headache-attributed burden from 1990 to 2023, along with the clinical and public health implications.

This manuscript was produced as part of the GBD Collaborator Network and in accordance with the GBD protocol.^9^

## Methods

### Overview

We give here an overview of the methods relevant for estimating the burden of headache disorders. Details can be found in appendix 1 (pp 3–17).

In the GBD hierarchy of diseases and injuries, headache disorders are a Level 3 cause, under neurological disorders (Level 2) and non-communicable diseases (Level 1). At Level 4, the most granular Level, headache disorders include migraine and tension-type headache. Since medication-overuse headache is a sequela of a pre-existing primary headache, its burden is wholly reattributed to migraine or tension-type headache in a ratio informed by a meta-analysis of three prospective longitudinal studies reporting the proportions of medication-overuse headache origin: 73·2% (95% uncertainty interval [UI] 63·7–81·0) for migraine and 26·8% (19·0–36·3) for tension-type headache.^10^, ^11^, ^12^

The case definitions for migraine, tension-type headache, and medication-overuse headache in GBD are based on the diagnostic criteria (appendix 1 pp 3–4). Separate models for definite and probable diagnoses exist, which are combined to give overall estimates of migraine and tension-type headache.

In GBD, disease burden is expressed as disability-adjusted life-years (DALYs), which are calculated as the sum of years of life lost (YLLs) to premature death and years lived with disability (YLDs).^13^ Since GBD does not estimate any deaths from headache disorders, DALYs are equivalent to YLDs for headache disorders. YLDs are calculated for each headache type by multiplying 1-year prevalence by proportion of time in symptomatic state and the associated disability weight (quantifying the health loss experienced while in a symptomatic state).

### Data sources

The input data informing GBD models were based on published population-based studies. The PubMed search strings used for systematic reviews are shown in appendix 1 (pp 4–5). Only studies reporting on the prevalence, incidence, or remission of migraine (definite or probable), tension-type headache (definite or probable), or medication-overuse headache were included.

The last systematic review informing GBD headache models was carried out in 2017. Data on migraine were extracted from 182 sources from 64 countries and 18 GBD regions, and on tension-type headache from 125 sources from 49 countries and 17 GBD regions. For medication-overuse headache, data were extracted from 47 sources from 30 countries and ten GBD regions. The vast majority of data (94·9%) related to prevalence. Western Europe had substantially more data sources than any of the other regions for each headache type, and data on definite migraine were the most abundant compared with the other headache diagnoses. Detailed information on data sources is available in appendix 1 (pp 5–11).

### Data standardisation

Extracted data were divided by age and sex where necessary. When studies reported prevalence for all sexes combined, estimates were divided by generating and applying a global sex ratio using the meta-regression—Bayesian, regularized, trimmed (MR-BRT) tool.^14^ Furthermore, when studies reported prevalence across large age groups, these were divided across 5-year age bins according to the global age pattern of prevalence modelled by a hierarchical Bayesian meta-regression modelling tool, DisMod-MR 2.1.^13^

To mitigate the influence of studies with low methodological quality and potential bias, we evaluated data for systematic differences and corrected accordingly using MR-BRT (appendix 1 pp 11–12). The following eight quality criteria for headache epidemiological studies were considered:^5^ representativeness of the population of interest; sampling quality; recall period; participation rate; survey method; validation of diagnostic instrument; diagnostic criteria; headache type (definite or probable) assumed. Detailed information on data standardisation is available in appendix 1 (pp 11–12).

### Estimation of prevalence

We used DisMod-MR 2.1 to estimate the prevalences of migraine, tension-type headache, and medication-overuse headache from 1990 to 2023, globally, regionally, and for individual countries. Estimates could be obtained in countries and regions where no primary studies were published using a geographical cascade where model fits were sequentially passed down as priors to the next geographical level (eg, super-region to region, region to country). In each model, it was assumed that there was no incidence before 5 years of age and that there was no mortality from headache disorders. No risk factors were used to inform prevalence estimation.

### Time in symptomatic state

In GBD 2023, we updated estimates of time in symptomatic state for migraine and tension-type headache, defined as the proportion of time during which a person has a headache.

Using the HARDSHIP (Headache-Attributed Restriction, Disability, Social Handicap and Impaired Participation) adult database,^15^ an individual participant data meta-analysis was performed among 41 653 individuals (19 590 males) aged 18–65 years from the general populations of 18 countries representing all seven GBD super-regions. In all contributing surveys, participants reported the number of days with headache during the preceding month or year and usual duration (hours) of their headache episodes. At the individual level, reported headache frequency was multiplied by reported usual duration of episodes to yield time in symptomatic state, and expressed as a proportion of all time.

Mean times in symptomatic state were thereby extracted for definite migraine, probable migraine, definite tension-type headache, and probable tension-type headache. Since time in symptomatic state varied across age and differed between males and females, age-specific and sex-specific estimates were made. As a trade-off between granularity and statistical power, and to reflect the age pattern, three age bins (<35 years, 35–49 years, and ≥50 years) were applied.

For medication-overuse headache, the same estimate of 53·2% time in symptomatic state as in previous iterations (based on a meta-analysis; appendix 1 p 16) was applied before reattribution to migraine (38·9% [28·4–49·4]) and tension-type headache (14·3% [7·0–21·6]) in the proportions stated earlier.

### Disability weights

All disability weights in GBD were determined through surveys of the general population and the use of lay descriptions of health states.^16^, ^17^ These weights range from 0 (indicating no health loss) to 1 (a health loss equivalent to being dead). The lay descriptions and corresponding disability weights of migraine, tension-type headache, and medication-overuse headache are shown in table 1. On average, during an attack, people with migraine have a health loss of 44·1% compared with people of full health. The equivalent values are 3·7% for tension-type headache and 22·3% for medication-overuse headache.

**Table 1:** Disability weights for headache disorders modelled in GBD^16^, ^17^

	**Health state description**	**Disability weight (95% UI)**
Migraine	Has severe, throbbing head pain and nausea that cause great difficulty in daily activities and sometimes confine the person to bed. Moving around, light, and noise make it worse.	0·441 (0·294–0·588)
Tension-type headache	Has a moderate headache that also affects the neck, which causes difficulty in daily activities.	0·037 (0·022–0·057)
Medication-overuse headache	Has daily headaches, felt as dull pain and often lasting all day, with poor sleep, nausea, and fatigue. The person takes medicine for the headaches, which provides little relief but is needed to avoid having worse symptoms.	0·223 (0·146–0·313)

UI=uncertainty interval.

### Presentation of results

The 95% UIs of YLDs were propagated from the 95% UIs of prevalence, time in symptomatic state, and disability weight estimates. These were derived from 250 samples from each estimate, using the 2·5th and 97·5th percentile values of the 250 values to determine the upper and lower bounds. After YLDs were estimated, an adjustment was made to account for comorbidity between all diseases in GBD.^13^

Age-standardised rates were estimated using the GBD standard population.^13^

Analyses were done in R (version 4.2.2).

### Role of the funding source

The funder of the study had no role in study design, data collection, data analysis, data interpretation, or writing of the report.

## Results

Table 2 shows the age-specific and sex-specific time in symptomatic state estimates for migraine and tension-type headache. Females consistently spent more time with headache across all age groups and case definitions than males. For definite migraine, time in symptomatic state increased steadily with age—in males ranging from 6·34% (95% UI 5·48–7·20) in those younger than 35 years to 8·67% (6·96–10·38) in those aged 50 years or older, and in females from 9·30% (8·53–10·06) in those younger than 35 years to 12·77% (11·37–14·18) in those aged 50 years or older. For both probable migraine and definite tension-type headache, time in symptomatic state was considerably lower than that for definite migraine, was similar in those younger than 35 years and those aged 35–49 years, increasing in those aged 50 years or older. Probable tension-type headache was associated with the least time in symptomatic state, but this also peaked in those aged 50 years or older.

**Table 2:** Estimates of mean time in symptomatic state for migraine and tension-type headache, by sex and age

	**Males**	**Females**
**Definite migraine**		
<35 years	6·34% (5·48–7·20)	9·30% (8·53–10·06)
35–49 years	7·86% (6·74–8·98)	10·13% (9·29–10·96)
≥50 years	8·67% (6·96–10·38)	12·77% (11·37–14·18)
**Probable migraine**		
<35 years	3·60% (2·94–4·26)	5·65% (4·96–6·33)
35–49 years	3·36% (2·78–3·94)	5·19% (4·53–5·84)
≥50 years	6·04% (4·50–7·58)	7·16% (6·07–8·25)
**Definite tension-type headache**		
<35 years	2·61% (2·30–2·92)	3·98% (3·57–4·39)
35–49 years	2·48% (2·13–2·83)	3·59% (3·16–4·02)
≥50 years	3·55% (2·98–4·12)	4·57% (3·92–5·21)
**Probable tension-type headache**		
<35 years	1·39% (0·93–1·85)	1·88% (1·42–2·34)
35–49 years	0·99% (0·75–1·23)	2·15% (1·42–2·88)
≥50 years	2·44% (1·30–3·58)	3·56% (1·89–5·23)

Data represent the mean percentage of time in a year in which a person is experiencing headache. Values in parentheses are 95% uncertainty intervals.

**Figure 1:**
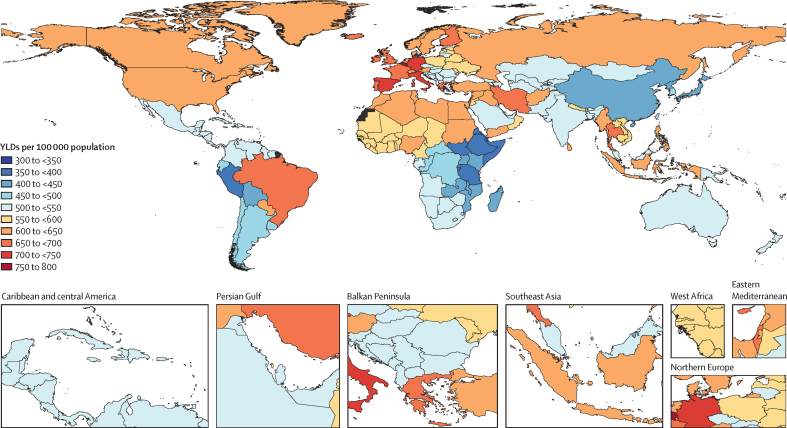
Age-standardised YLD rates due to headache disorders, by country, all sexes, 2023 YLDs=years lived with disability.

In 2023, 2·9 billion individuals (2·6-3·1) were estimated to have a headache disorder, equating to a global age-standardised prevalence of 34·6% (31·6–37·5), which was higher among females (37·5% [34·4–40·4]) than males (31·8% [28·9–34·8]). The global YLD count was 45·5 million (31·4–62·0), equating to an age-standardised rate of 541·9 YLDs (373·4–739·9) per 100 000 population, over twice as high among females (739·9 YLDs [511·2–1011·5] per 100 000) as among males (346·1 YLDs [240·4–481·8] per 100 000). A map of age-standardised YLDs attributed to headache disorders is shown in figure 1.

Tension-type headache was more common than migraine with a global age-standardised prevalence of 24·9% (22·0–28·2), which was relatively similar between males (24·4% [21·6–27·6]) and females (25·5% [22·4–28·7]). The global age-standardised prevalence of migraine was 14·1% (12·1–15·9), and was significantly higher among females (17·6% [15·2–19·9]) than males (10·5% [9·0–12·1]).

In 2023, migraine alone caused 40·9 million (27·1–56·9) YLDs. In terms of age-standardised YLD rates, migraine ranked eighth among all conditions, with a rate of 487·5 YLDs (323·0–678·8) per 100 000. In 1990, migraine ranked fifth, with a similar rate of 481·6 YLDs (319·6–668·5) per 100 000. For tension-type headache, the respective rates were 54·4 YLDs (36·7–77·3) per 100 000 in 2023 and 55·5 YLDs (37·6–79·0) per 100 000 in 1990.

**Figure 2:**
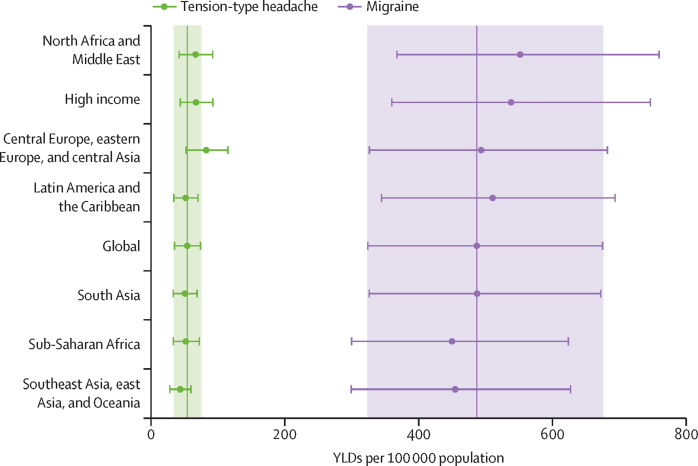
Age-standardised rate of YLDs attributed to migraine and tension-type headache globally and by super-region, 2023 Points and error bars represent means and 95% uncertainty intervals. Vertical lines show the mean global estimates and shaded areas are global 95% uncertainty intervals. YLDs=years lived with disability.

Table 3 shows age-standardised estimates of prevalence and YLDs for migraine and tension-type headache in 1990 and 2023 for seven GBD super-regions and globally. The equivalent country-specific estimates are shown in appendix 1 (pp 17–31). The north Africa and Middle East super-region had the highest age-standardised YLD rate for migraine in 2023 (552·8 YLDs [366·5–757·9] per 100 000 population), closely followed by the high-income super-region (538·7 YLDs [358·6–748·4] per 100 000). Central Europe, eastern Europe, and central Asia had the highest age-standardised YLD rate for tension-type headache (82·9 YLDs [55·7–118·9] per 100 000). The lowest rate for migraine was found in sub-Saharan Africa (450·8 YLDs [299·5–623·9] per 100 000), and for tension-type headache the lowest rate was in southeast Asia, east Asia, and Oceania (43·9 YLDs [29·6–62·9] per 100 000). There was a high degree of overlap between the 95% UIs of the different GBD super-regions, and, for migraine, the estimates for all regions were placed well within the 95% UI of the global estimate (figure 2).

**Table 3:** Age-standardised prevalence and YLD rates attributed to migraine and tension-type headache globally and by super-region, 1990 and 2023

	**Prevalence per 100 000 population**		**YLDs per 100 000 population**	
	1990	2023	1990	2023
**Migraine**				
Global	13 817 (11 925–15 630)	14 053 (12 067–15 881)	481·6 (319·6–668·5)	487·5 (323·0–678·8)
Central Europe, eastern Europe, and central Asia	13 691 (11 785–15 571)	13 624 (11 687–15 500)	498·8 (326·9–686·7)	493·8 (323·3–677·6)
High income	15 588 (13 505–17 689)	15 553 (13 384–17 586)	543·5 (358·7–752·2)	538·7 (358·6–748·4)
Latin America and Caribbean	14 797 (12 635–16 806)	14 818 (12 600–16 807)	511·6 (343·6–706·0)	511·1 (341·5–704·7)
North Africa and Middle East	15 090 (12 912–17 382)	15 157 (13 103–17 359)	552·3 (365·9–763·3)	552·8 (366·5–757·9)
South Asia	14 606 (12 619–16 739)	14 611 (12 587–16 633)	484·3 (327·1–671·5)	487·8 (324·1–684·3)
Southeast Asia, east Asia, and Oceania	12 136 (10 450–13 732)	12 977 (11 190–14 700)	424·9 (285·0–594·1)	455·1 (300·9–633·0)
Sub-Saharan Africa	12 892 (10 968–14 734)	12 987 (11 053–14 886)	444·7 (293·5–617·9)	450·8 (299·5–623·9)
**Tension-type headache**				
Global	25 060 (22 168–28 223)	24 940 (21 975–28 157)	55·5 (37·6–79·0)	54·4 (36·7–77·3)
Central Europe, eastern Europe, and central Asia	31 434 (27 797–35 248)	31 450 (27 749–35 393)	84·1 (56·3–120·7)	82·9 (55·7–118·9)
High income	32 241 (28 708–36 399)	31 985 (28 353–35 954)	67·8 (46·7–96·2)	67·5 (46·4–96·2)
Latin America and Caribbean	25 821 (22 799–29 061)	25 650 (22 609–29 089)	51·7 (34·9–73·5)	52·0 (35·1–73·7)
North Africa and Middle East	23 777 (20 529–27 186)	23 979 (20 765–27 289)	66·4 (43·6–97·5)	66·9 (44·0–97·4)
South Asia	25 979 (23 078–29 288)	25 991 (23 077–29 267)	50·2 (34·0–70·8)	50·8 (34·3–71·7)
Southeast Asia, east Asia, and Oceania	19 330 (17 045–21 799)	20 849 (18 309–23 506)	41·9 (28·2–60·1)	43·9 (29·6–62·9)
Sub-Saharan Africa	22 842 (19 842–25 916)	22 746 (19 761–25 792)	51·8 (34·6–75·3)	52·3 (34·9–75·9)

Values in parentheses are 95% uncertainty intervals. YLD=years lived with disability.

**Figure 3:**
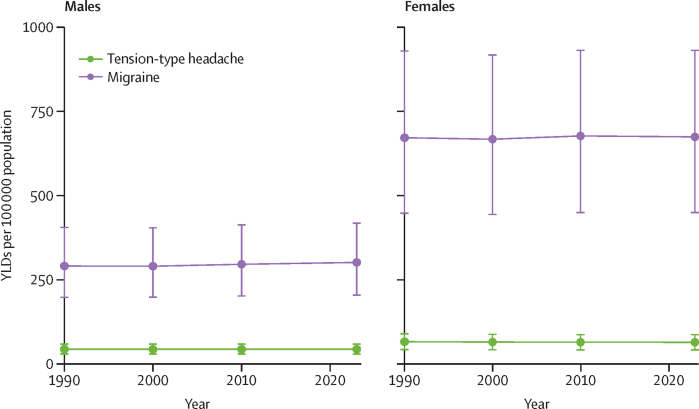
Global age-standardised rates of YLDs attributed to migraine and tension-type headache, by sex, 1990–2023 Points and error bars represent means and 95% uncertainty intervals. YLDs=years lived with disability.

There were minimal changes in age-standardised YLD rates for both migraine and tension-type headache between 1990 and 2023 (figure 3).

For migraine, medication-overuse headache contributed 6·0% of the prevalence estimates for males and 5·0% for females, but 22·6% of the YLD estimates for males and 14·1% for females. For tension-type headache, medication-overuse headache contributed only 0·9% of the prevalence estimates for males and 1·3% for females, but 58·9% and 56·1% of the YLD estimates, respectively. Hence, 20·8% of headache-attributed YLDs came from medication-overuse headache.

Details of how the underlying headache diagnoses (definite diagnosis, probable diagnosis, and medication-overuse headache) contributed to age-standardised prevalence and YLD rates can be found in appendix 1 (pp 31–33).

## Discussion

Headache disorders continue to be among the most prevalent and burdensome conditions worldwide, as evidenced by the 2·9 billion individuals affected in 2023. The notable age-standardised rate of 541·9 YLDs per 100 000 population positions headache disorders as the sixth most disabling group of conditions worldwide.^13^ Our findings highlight the pressing need for effective management and prevention strategies for headache disorders.

Despite that, tension-type headache was almost twice as prevalent as migraine (24·9% *vs* 14·1%), migraine accounted for 90% of YLDs attributed to headache disorders in 2023. This was because of the substantially higher disability weight for migraine (0·441 *vs* 0·037),^16^ a reflection of the more severe symptoms associated with migraine episodes than with tension-type headache episodes.^6^ Notably, these disability weights do not currently differentiate between probable and definite diagnoses. Mostly, individuals are diagnosed with probable rather than definite migraine because attack duration either falls short of or exceeds the 4–72 h specified in the diagnostic criteria,^18^, ^19^ which is already factored into the time in symptomatic state estimates. Nonetheless, a proportion of individuals with probable migraine—those who fulfil the duration criterion—should probably be assigned a lower disability weight, since, by not fulfilling another criterion (and thereby falling short of meeting criteria for definite migraine), they are likely to have fewer debilitating symptoms (eg, nausea or vomiting). Conversely, a proportion of individuals with probable tension-type headache should probably be assigned a higher disability weight, for the converse reason (ie, by not fulfilling one diagnostic criterion of tension-type headache, they have at least one migraine-like symptom, such as nausea or vomiting).

It should be emphasised that, within the scheme of the ICHD diagnostic criteria, “probable” diagnoses represent probable cases of migraine or tension-type headache; they do not recognise “probable migraine” and “probable tension-type headache” as entities distinct from “definite migraine” or “definite tension-type headache” (terms that are not used in the ICHD). The diagnostic criteria of ICHD^6^ prioritise specificity over sensitivity because their primary intent is uniformity of meaning in research.^20^ In clinical settings, probable diagnoses can be applied tentatively, with follow-up allowing for confirmation or rejection in the course of management. Such confirmation or rejection is not possible in cross-sectional epidemiological studies, which are the predominant source of data on prevalence and attributable burden.

More YLDs are attributed to migraine also because individuals with migraine spend a considerably greater proportion of their time with headache than do those with tension-type headache (table 2). The incorporation of age-specific and sex-specific estimates of time in symptomatic state represents a methodological refinement in GBD 2023. In previous GBD iterations, estimates of time in symptomatic state were based on a meta-analysis of 19 surveys (n=29 062, age 18–65 years), of which three surveys were of clinical samples and another three were of samples from patient organisations. By contrast, the new estimates were derived from high-quality population-representative individual participant data (n=41 653). This new meta-analysis greatly reduced uncertainty in the YLD estimates (the 95% UIs of global age-standardised YLDs per 100 000 population was 117·6–1245·4 in GBD 2021^2^ and 373·4–739·9 in GBD 2023), and increased the evident difference in YLDs between females (739·9 [511·2–1011·5] per 100 000) and males (346·1 [240·4–481·8] per 100 000). Also of interest was the positive association between age and time in symptomatic state, especially for definite migraine (not to be confused with the inverted U-shaped relationship between age and prevalence). The reason for this association is not clear. One explanation could be that those who are most severely affected (longest headache duration or highest frequency) are least likely to go into remission, such that the mean individual time in symptomatic state is greater among older people with migraine. In support of this, chronic migraine is reportedly more likely than episodic migraine to persist into later life.^21^

Of profound clinical relevance, and implication for public health, was the very substantial burden attributed to medication-overuse headache. Our findings indicate that more than 20% of headache-attributed burden would be mitigated or completely averted if an important minority of people with headache did not overuse medication. This underpins the need to raise awareness, among both people with headache and health-care providers, about the risks associated with the overuse of acute medication. The studies contributing to GBD make clear that medication overuse is a global problem, not restricted to high-income countries.

Our analysis revealed no substantial differences in headache-attributed YLDs between the various GBD super-regions or across time. Notably, despite drawing data from 41 653 individuals, our meta-analysis of time in symptomatic state was unequipped for a detailed breakdown by location or year. Our results therefore imply that there has been no meaningful change in the age-standardised prevalence of migraine or tension-type headache over the past three decades, suggesting no modulation of the underlying, and largely unknown, causes of these disorders. An important caveat to this observation is that the last literature review informing our prevalence estimates was done in 2017. Any potential effect of SARS-CoV-2 and the resultant pandemic is, therefore, not captured. Additionally, any recent shifts in headache-attributed burden following the introduction of new migraine-specific medications^22^ such as CGRP inhibitors (to which time in symptomatic state would be more sensitive than prevalence) are not captured. Any such shifts would have been very small, because, even where these new medications are available (and in many parts of the world they are not),^23^ coverage is minimal.

These updated findings of GBD strongly support the call for better headache care in all parts of the world.^24^, ^25^ Structured headache services, based in primary care, and tailored to local health-care infrastructure and resources, are the solution proposed by the Global Campaign against Headache.^26^, ^27^, ^28^ The humanitarian argument for alleviating suffering is backed by theoretical modelling of the expected cost-effectiveness of structured headache services in all economies, with the potential for cost saving in some economies (taking the indirect costs of lost productivity into account).^28^ Structured headache services are built on educational pillars, guiding people to appropriate levels of care (only a small minority need specialist care) while also promoting the appropriate use of efficacious treatments and discouraging overuse. In this context, it should be noted that efficacious treatments exist, but have low availability in many parts of the world.^23^ Pharmaceutical companies, especially those marketing the new drugs, have both a responsibility and an opportunity here: rather than fighting to increase share in the saturated market of high-income countries, they should work together to expand the market, taking drugs affordably to low-income and middle-income countries.

To date, this is the most comprehensive analysis of population-level burden attributed to headache disorders. While this is a major strength, the study has several limitations, some already discussed. Prevalence data were scarce (or non-existent) in numerous countries, especially low-income countries and particularly with regard to tension-type headache and medication-overuse headache. Because of this, we caution against overly granular geographical comparisons, and in this Article compare only broader regional areas. Correction of prevalence estimates from studies possibly subject to systematic biases were also influenced by scarcity of data, which might have affected the reliability of the corrections. Correction was nevertheless perceived as preferable, as exclusion would have further decreased the number of data sources. GBD has models for only three headache types; although these three (among the 200 described in the ICHD)^6^ are by far the greatest contributors to population health loss of headache disorders, and although any burden associated with secondary headaches is accounted for through the modelling of their underlying causes, there is unmeasured burden attributable to a few primary headaches other than migraine and tension-type headache (in particular, cluster headache). However, these primary headaches are relatively uncommon (cluster headache has a prevalence of about 0·1%),^5^ so at population level they account for only a small number of YLDs, while the scarcity of robust epidemiological data prevents the construction of reliable models. The burden attributed to medication-overuse headache was wholly reattributed to migraine or tension-type headache. In a very small proportion of cases, another primary headache, such as chronic cluster headache or new daily persistent headache, might have been the preexisting headache type,^6^ but these other headaches are relatively rare, and medication-overuse headache arising from them even rarer. Another limitation pertaining to our estimates for medication-overuse headache is the fact that they were derived from cross-sectional prevalence studies. Diagnoses therefore depended on the association of medication with high headache frequency, without evidence of causation. This is an insurmountable limitation of population-based studies. The new estimates of time in symptomatic state were derived from samples of people aged 18–65 years, and their validity for children, adolescents, and those aged over 65 years is unproven. Finally, no GBD risk factors (eg, high blood pressure, air pollution, malnutrition, or high LDL) informed our estimates.

In conclusion, headache disorders remain a very substantial global health concern as the sixth leading cause of health loss, affecting 2·9 billion individuals in 2023. The burden of headache disorders is twice as high among females, who not only have a higher prevalence of migraine but also, at an individual level, spend a greater proportion of their time with headache than males. While simple and cost-effective treatment options exist, they fail to reach many who would benefit from them, partly because headache care is poorly organised but also, and importantly, because of educational failures leading to suboptimal utilisation. The latter includes medication misuse, with regard to which the disproportionate effect of medication-overuse headache offers a compelling opportunity for health-loss prevention through education of both the population and health-care professionals.

### GBD 2023 Headache Collaborators

### Affiliations

### Contributors

### Data sharing

This study follows the Guidelines for Accurate and Transparent Health Estimates Reporting (GATHER). To download citations and metadata for the input data sources used in the GBD 2023 analyses presented in this study, please visit the GBD 2023 Sources Tool (https://ghdx.healthdata.org/gbd-2023/sources).

## Declaration of interests

Ali Ahmed reports support for their participation in the current manuscript from the US National Institute of Mental Health (NIMH) RO1MH126768 and R21MH132406; grants or contacts from the U.S. National Institute of Mental Health (NIMH) RO1MH126768 and R21MH132406 outside the submitted work. S Bhaskar reports grants or contracts from Japan Society for the Promotion of Science (JSPS), Japanese Ministry of Education, Culture, Sports, Science and Technology (MEXT), Grant-in-Aid for Scientific Research (KAKENHI; grant ID 23KF0126), JSPS and the Australian Academy of Science, JSPS International Fellowship (grant ID P23712); leadership or fiduciary role in other board, society, committee or advocacy group, paid or unpaid, with Rotary District 9675 (Sydney, Australia), Global Health & Migration Hub Community, Global Health Hub Germany (Berlin, Germany), PLoS One, BMC Neurology, Frontiers in Neurology, Frontiers in Stroke, Frontiers in Public Health, Journal of Aging Research, Neurology International, Diagnostics, and BMC Medical Research Methodology, College of Reviewers, Canadian Institutes of Health Research (CIHR), Government of Canada, World Headache Society (Bengaluru, India), Cariplo Foundation (Milan, Italy), National Cerebral and Cardiovascular Center, Department of Neurology, Division of Cerebrovascular Medicine and Neurology, Suita (Osaka, Japan), Cardiff University Biobank (Cardiff, UK), Rotary Reconciliation Action Plan, Japan Connect (Osaka, Japan); all outside the submitted work. E Caronna reports grants from Juan Rodes—Instituto Carlos III; payment or honoraria for lectures, from Organon, TEVA, and Dr Reddy's; support for attending meetings from Organon and TEVA; and other financial or non-financial interests as Junior Editor for Cephalalgia; all outside the submitted work. A K Demetriades reports Leadership or fiduciary role in other board, society, committee or advocacy group, unpaid, with EANS (European Association of Neurosurgical Societies) as a Board member, Global Neuro Foundation as a Board Member, and with AO SPINE as Steering Committee Member for Knowledge Forum Degenerative, all outside the submitted work. X Ding reports grants or contracts from the American Heart Association through a 2-year predoctoral fellowship (DOI: 10.58275/AHA.25PRE1373497.pc.gr.227106); quarterly payments made to their institution, all outside the submitted work. A Faro reports support for their participation in the present manuscript from National Council for Scientific and Technological Development (CNPq, Brazil). A Hassan reports consulting fees from Novartis, Sanofi Genzyme, Biologix, Astra Zeneca, Pfizer, Merz, Roche, Merck, Hikma Pharma, Janssen, Inspire Pharma, Future Pharma, Elixir pharma; Payment or honoraria for lectures, presentations, speakers bureaus, manuscript writing or educational events from Novartis, Allergan, Abbvie, Merck, Biologix, Viatris, Pfizer, Eli Lilly, Janssen, Roche, Sanofi Genzyme, Bayer, AstraZeneca, Hikma Pharma, Al Andalus, Chemipharm, Lundbeck, Elixir, EvaPharma, Inspire Pharma, Future Pharma and Habib Scientific Office, and Everpharma; support for attending meetings and/or travel from Novartis, Allergan, Merz, Pfizer, Merck, Biologix, Roche, Sanofi Genzyme, Bayer, Hikma Pharma, Chemipharm, and Al Andalus and Clavita Pharm; leadership or fiduciary role in other board, society, committee or advocacy group, paid or unpaid, as Vice President of MENA headache society, board member of the Multiple Sclerosis Chapter of the Egyptian Society of Neurology, board member of the Headache Chapter of the Egyptian Society of Neurology, member of committee of Education of the international Headache Society (IHS), membership committee of IHS, and regional committee of HIS; all outside the submitted work. A K Hus⊘y reports payment or honoraria for a presentation held in January 2025 for headache nurses on treatment of migraine lectures from Teva Pharmaceuticals; leadership or fiduciary role in other board, society, committee or advocacy group, unpaid, as an editorial board member of The Journal of Headache and Pain; all outside the submitted work. I M Ilic reports support for their participation in the current manuscript from Ministry of Science, Technological Development and Innovation of the Republic of Serbia (no. 451-03-137/2025-03/200110). N E Ismail reports leadership or fiduciary role in other board, society, committee or advocacy group, unpaid, as Bursar and Council Member, Malaysian Academy of Pharmacy, Malaysia and as a committee member, Malaysian Pharmacists Society Education Chapter Committee, all outside the submitted work. K Krishan reports non-financial support from the UGC Centre of Advanced Study, CAS II, awarded to the Department of Anthropology, and RUSA 2.O grant awarded to Panjab University (Chandigarh, India), outside the submitted work. B Oancea reports support for their participation in the present manuscript from Ministry of Research, Innovation and Digitalization through the Core Program of the National Research, Development and Innovation Plan 2022–2027, project number PN 23-02-0101, contract number 7N/2023, and PNRR/2022/C9/MCID/I8 project 760096. H Sarma reports support for their participation in the current manuscript from Department of Botany. Bodoland University. J A Singh reports consulting fees from ROMTech, Atheneum, Clearview Healthcare Partners, American College of Rheumatology, Yale, Hulio, Horizon Pharmaceuticals, DINORA, ANI/Exeltis USA, Frictionless Solutions, Schipher, Crealta/Horizon, Medisys, Fidia, PK Med, Two Labs, Adept Field Solutions, Clinical Care Options, Putnam Associates, FocusForward, Navigant Consulting, Spherix, MedIQ, Jupiter Life Science, UBM, Trio Health, Medscape, WebMD, Practice Point Communications, and the National Institutes of Health; payment or honoraria for lectures, presentations, speakers bureaus, manuscript writing or educational events from Simply Speaking; support for attending meetings and/or travel from OMERACT, an international organisation that develops measures for clinical trials and receives arm's length funding from 12 pharmaceutical companies, as past steering committee member to attend their meeting every 2 years; participation on a Data Safety Monitoring Board or Advisory Board with FDA Arthritis Advisory Committee (unpaid); leadership or fiduciary role in other board, society, committee or advocacy group, paid or unpaid as a past steering committee member of the OMERACT; stock or stock options in Atai Life Sciences, Kintara Therapeutics, Intelligent Biosolutions, Acumen Pharmaceutical, TPT Global Tech, Vaxart Pharmaceuticals, Atyu Biopharma, Adaptimmune Therapeutics, GeoVax Labs, Pieris Pharmaceuticals, Enzolytics, Seres Therapeutics, Tonix Pharmaceuticals Holding Corp, Aebona Pharmaceuticals, and Charlotte's Web Holdings, and previously owned stock options in Amarin, Viking, and Moderna Pharmaceuticals; all outside the submitted work. T J Steiner reports support for travel and accommodation from European Headache Federation, Norwegian Headache Research Centre, International Headache Society, Danish Headache Society; leadership or fiduciary role in other board, society, committee or advocacy group, paid or unpaid, as Director and Trustee of Lifting The Burden: the Global Campaign against Headache; all outside the submitted work. S Straube reports grants or contracts from Workers’ Compensation Board—Alberta, through payments to their institution; payment or honoraria for lectures, presentations, speakers bureaus, manuscript writing or educational events from Occupational Medicine Specialists of Canada; leadership or fiduciary role in other board, society, committee or advocacy group, paid or unpaid, as an editorial board member of SN Comprehensive Clinical Medicine, The Journal of Headache and Pain, Scandinavian Journal of Pain, MSI Foundation, and receiving honoraria from Ontario Tech University to serve on the scientific committee for the development of evidence-based care pathways for the Alberta Care-First auto insurance reform (Ontario Tech University received a grant from the Government of Alberta); all outside the submitted work. V-S Teriotis reports grants or contracts from European Academy of Neurology, European Committee for Treatment and Research in Multiple Sclerosis; support for attending meetings and/or travel from Inovis, Genesis Pharma, Novartis; all outside the submitted work. M Zielińska reports other financial or non-financial interests in Alexion, AstraZeneca Rare Diseases as an employee.
