# Supplementary material for: Global, regional, and national burden of headache disorders, 1990–2023: a systematic analysis for the Global Burden of Disease Study 2023
Source: Lancet Neurol. 2025 Dec;24(12):1005–15. doi: 10.1016/S1474-4422(25)00402-8 (PMC12612381; doi:10.1016/S1474-4422(25)00402-8)
Supplement: Supplementary appendix [file mmc1.pdf]

THE LANCET
Neurology

Supplementary appendix 1

This appendix formed part of the original submission and has been peer reviewed. We post it as supplied by the authors.

Supplement to: GBD 2023 Headache Collaborators. Global, regional, and national burden of headache disorders, 1990–2023: a systematic analysis for the Global Burden of Disease Study 2023. *Lancet Neurol* 2025; **24:** 1005–15.

Global, regional, and national burdens of headache disorders, 1990-2023: a systematic analysis for the Global Burden of Disease Study 2023

Appendix

Table of Contents

List of tables **2**
1. List of figures **2**
2. Headaches Flow Chart **3**
3. Case definitions **3**
3.1. Migraine *3*
3.2. Tension-type headache *4*
3.3. Medication overuse headache *4*
4. Input data prevalence **4**
4.1. Systematic Reviews *4*
4.1.1. Migraine *4*
4.1.2. Tension-type headache *5*
4.1.3. Medication overuse headache *5*
4.2. Data sources *5*
4.3. Data standardization *11*
4.3.1. Age and sex splitting *11*
4.3.2. Study quality and bias adjustment *11*
4.4. Total Headache Model Data Adjustment *12*
5. Modelling strategy **14**
5.1. Migraine *14*
5.2. Tension-type headache *14*
5.3. Medication overuse headache *14*
6. Time Symptomatic Analysis **14**
6.1. Input data time symptomatic *14*
6.2. Mathematical calculation *15*
6.3. Time symptomatic estimates *16*
7. Headache Severity Distribution **16**
8. Supplementary results **17**
8.1. Country-specific prevalence and YLD estimates *17*
8.2. Prevalence and burden of underlying headache diagnosis *31*
9. References **33**

List of tables

Supplementary table 1. Data source count on migraine, tension-type headache and medication overuse headache 7
Supplementary table 2. Study-specific quality and bias covariate definitions... 11
Supplementary table 3. MR-BRT Crosswalk Adjustment Factors for Headaches 12
Supplementary table 4. Time symptomatic input data... 15
Supplementary table 5. Proportion of time symptomatic by age, sex and headache type 16
Supplementary table 6. Medication overuse headache: combined proportion split and time symptomatic proportion 16
Supplementary table 7. Severity distribution, details on the severity levels for headaches and the associated disability weight (DW) with that severity... 16
Supplementary table 8. Country-specific age-standardized prevalence and years lived with disability (YLD) rates (per 100,000 persons) attributed to migraine 17
Supplementary table 9. Country-specific age-standardized prevalence and years lived with disability (YLD) rates (per 100,000 persons) attributed to tension-type headache... 24
Supplementary table 10. Global age-standardized prevalence and YLD estimates for migraine and tension-type headache by sex and underlying headache diagnosis (definite diagnosis, probable diagnosis, or medication overuse headache) 31

1. **List of figures**

Supplementary figure 1. GBD Headache disorder Model Flow chart 3
Supplementary figure 2. Map of input data sources informing migraine 6
Supplementary figure 3. Map of input data sources informing tension-type headache.. 6
Supplementary figure 4. Map of input data sources informing medication overuse headache 6
Supplementary figure 5. Definite migraine to total migraine adjustment.. 13
Supplementary figure 6. Definite TTH to total TTH adjustment... 13
Supplementary figure 7. Meta-analysis informing medication overuse headache split into migraine or tension-type headache 14
Supplementary figure 8. Global prevalence of migraine and tension-type headache in males and females stratified by age, broken down by underlying headache diagnosis (definite diagnosis, probable diagnosis, or medication overuse headache 32
Supplementary figure 9. Global years lived with disability (YLD) rates (per 100,000 persons) of migraine and tension-type headache in males and females stratified by age, broken down by underlying headache diagnosis (definite diagnosis, probable diagnosis, or medication overuse headache) 33

2. **Headaches Flow Chart**

Supplementary figure 1. GBD Headache disorder Model Flow chart

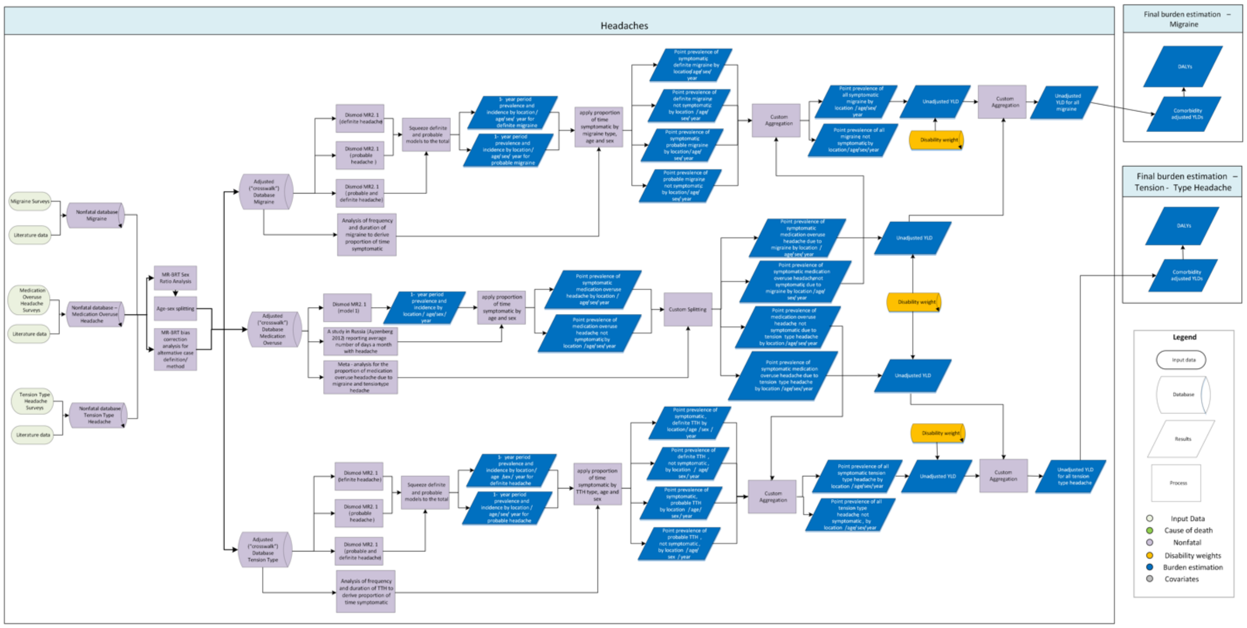

In GBD 2023, we report estimates for migraine and tension-type headaches (TTH). However in our modelling process, we collect data on more granular information pertaining to migraine and TTH by including definite and probable diagnosis types for each headache. Additionally, we also collect data on medication overuse headache (MOH) which informs estimates for migraine and TTH.

3. Case definitions

3.1. Migraine

Migraine is a disabling primary headache disorder, typically characterised by recurrent moderate or severe unilateral pulsatile headaches. The two major types are migraine without aura and migraine with aura (transient neurological symptoms). In GBD, we do not currently model health loss attributed to migraine aura symptoms. The reference diagnostic criteria for migraine is adapted from the International Classification of Headache Disorders (ICHD)-3 [1], where at least one headache episode in the past year needs to fulfill the following criteria:

1. Headache attacks lasting 4–72 hours (untreated or unsuccessfully treated)
2. Headache has at least two of the following four characteristics:
    a. Unilateral location
    b. Pulsating quality
    c. Moderate or severe pain intensity
    d. Aggravation by or causing avoidance of routine physical activity
3. During headache at least one of the following:
    a. Nausea and/or vomiting
    b. Photophobia and phonophobia
4. Not better accounted for by another ICHD-3 diagnosis

The migraine category includes both definite migraine cases and probable migraine cases. Definite migraine is a headache that satisfies all the criteria outlined above, while probable migraine satisfies all but one of the above criteria. Studies that have looked at the reasons for cases with probable headache not fulfilling criteria for definite

diagnosis have suggested that most often it is the duration criterion that is left unfilled [2-6].In most epidemiological studies, which results we rely on in our estimates, the last criterion (not better accounted for by another ICHD-3 diagnosis) cannot be fully ascertained due to operational constraints.

3.2. Tension-type headache

TTH is characterised by a dull, non-pulsatile, diffuse, band-like (or vice-like) pain of mild to moderate intensity in the head. The reference diagnostic criteria for TTH are adapted from the ICHD-3 [1], in which at least one headache episode in the past year needs to fulfill the following criteria:

1. Lasting from 30 minutes to 7 days
2. At least two of the following four characteristics:
    a. Bilateral location
    b. Pressing or tightening (non-pulsating) quality
    c. Mild or moderate intensity
    d. Not aggravated by routine physical activity such as walking or climbing stairs
3. Both of the following:
    a. No nausea or vomiting
    b. No more than one of photophobia or phonophobia
4. Not better accounted for by another ICHD-3 diagnosis

TTH category includes both definite TTH cases and probable TTH cases. Definite TTH is a headache that satisfies all criteria outlined above, while probable TTH satisfies all but one of the above criteria.

3.3. Medication overuse headache

Both migraine and TTH can give rise to MOH, with the following ICHD-3 [1] diagnostic criteria:

1. Headache occurring ≥15 days/month in a patient with a pre-existing headache disorder
2. Regular overuse for >3 months of one or more drugs that can be taken for acute and/or symptomatic treatment of headache
3. Not better accounted for by another ICHD-3 diagnosis

ICHD-3 explicitly states that, when a person fulfils criteria for both migraine and MOH, both diagnoses should be given. However, our GBD headache collaborators indicated that in population survey practice, a screening question to identify chronic headache (headache on ≥15 days/month) is often applied first, followed by questions to determine if medication overuse is present. This means the diagnoses of migraine and MOH become mutually exclusive (obviating any potential problem of double counting).

4. Input data prevalence

There are no updates to input data for GBD 2023. This is a major limitation and caution is warranted when interpreting estimates and trends in years post 2017. A systematic review is underway for the next GBD round.

4.1. Systematic Reviews

4.1.1. Migraine

We last conducted a systematic review of migraine for GBD 2017, which covered papers published through September 2017. The search string for this review was (((((("migraine disorders"[MeSH Terms] OR migraine[All Fields]) AND ((prevalence[Title/Abstract] OR incidence[Title/Abstract] OR remission[Title/Abstract] OR epidemiology[Title/Abstract]))))))).

Inclusion criteria of the systematic review were:

- Representative, population-based surveys

- Reporting of prevalence of migraine

In GBD 2017 we decided to exclude medical claims data as the adjustment needed to make the claims data comparable to population representative surveys was unstable.

4.1.2. Tension-type headache

We last conducted a systematic review of TTH for GBD 2017, which covered papers published through September 2017. The search string for this review was ((((("headache"[MeSH Terms]) OR ("headache"[Title/Abstract] AND "tension"[Title/Abstract])) AND ("epidemiology"[Title/Abstract] OR "prevalence"[Title/Abstract] OR "incidence"[Title/Abstract] OR "remission"[Title/Abstract])))).

Inclusion criteria of the systematic review were:

- Representative, population-based surveys
- Reporting of prevalence of TTH

In GBD 2017 we decided to exclude medical claims data, as the adjustment needed to make the claims data comparable to population representative surveys was unstable.

4.1.3. Medication overuse headache

We last conducted a systematic review of MOH for GBD 2017, which covered papers published through September 2017. The search string for this review was (("headache"[MeSH Terms] OR "headache"[Title/Abstract]) AND ("pharmaceutical preparations"[MeSH Terms] OR "pharmaceutical preparations"[Title/Abstract] OR "medication"[Title/Abstract]) AND ("epidemiology"[Title/Abstract] OR "prevalence"[Title/Abstract] OR "incidence"[Title/Abstract] OR "remission"[Title/Abstract])).

Inclusion criteria of the systematic review were:

- Representative, population-based surveys
- Reporting of prevalence of MOH

4.2. Data sources

Supplementary figures 2-4 and Supplementary table 1 show the number of disaggregated input data sources informing our migraine, TTH, and MOH models (white areas are areas without input data). For all three headache types, data were available from all seven GBD super regions.

Importantly, the term *source* here, is not equivalent to a single published study, rather GBD utilizes a source counting strategy that also account for different data collection location and years. In Supplementary table 1 and Supplementary figures 2-4, number of sources reported are disaggregated by country and territories.

Supplementary figure 2. Map of input data sources informing migraine

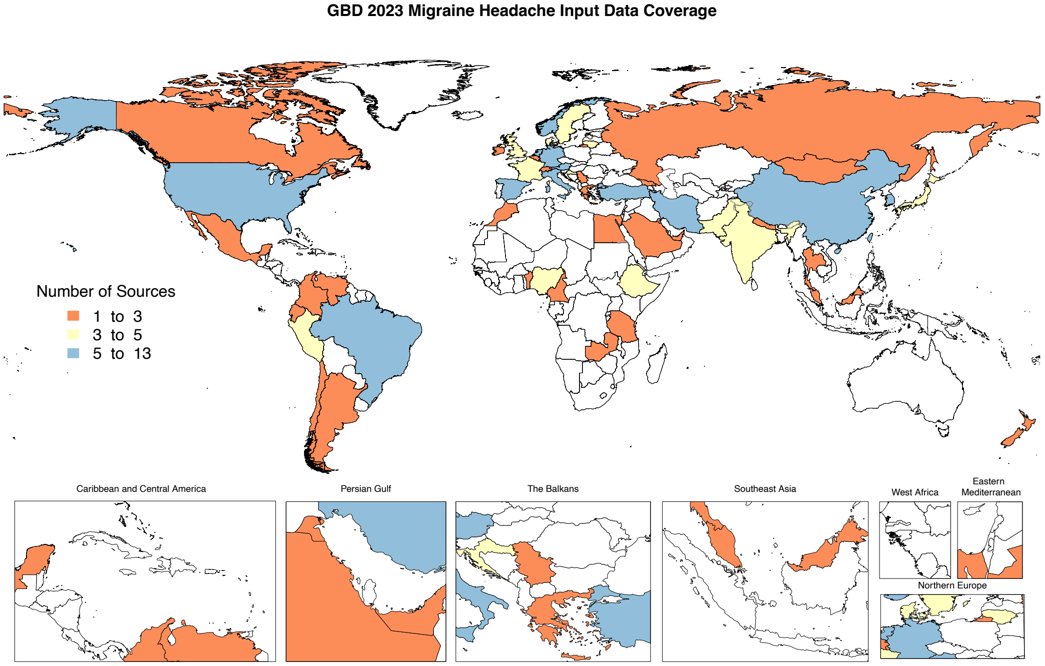

Supplementary figure 3. Map of input data sources informing tension-type headache

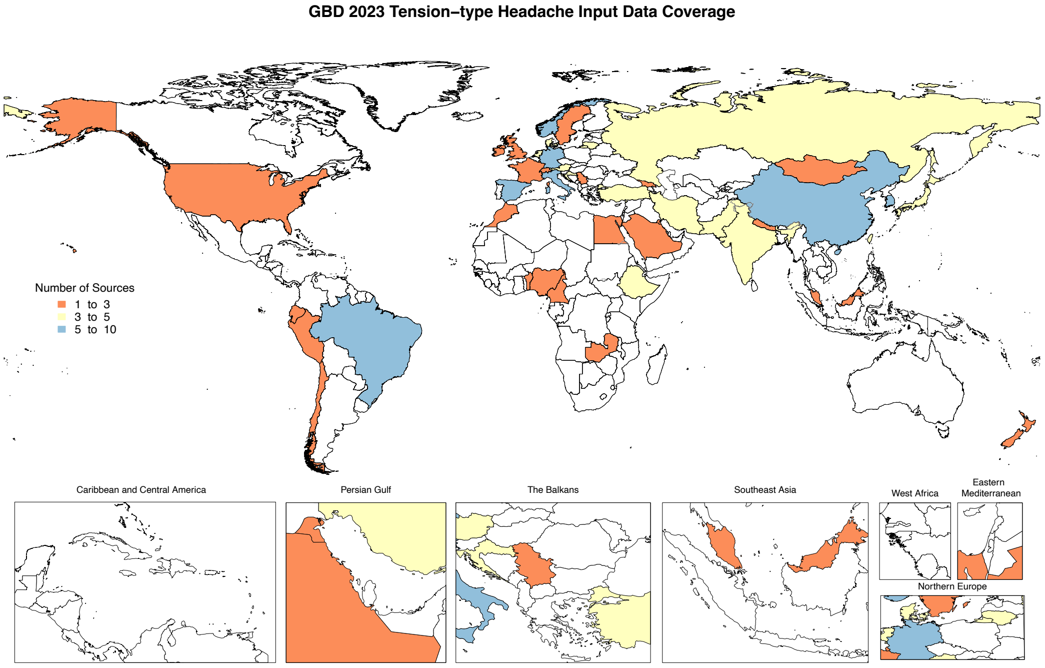

Supplementary figure 4. Map of input data sources informing medication overuse headache

GBD 2023 Medication Overuse Headache Input Data Coverage

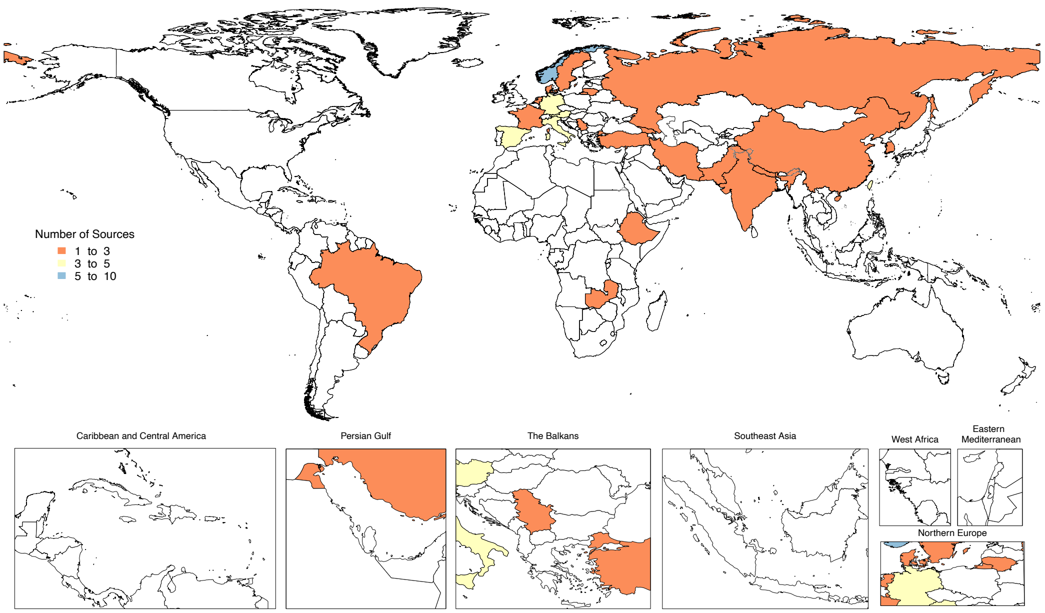

Supplementary table 1. Data source count on migraine, tension-type headache and medication overuse headache

Measure	GBD super region	GBD region	Location	Source count
		**Migraine**		
Prevalence	Southeast Asia, East Asia, and Oceania	East Asia	China	8
Prevalence	Southeast Asia, East Asia, and Oceania	East Asia	Taiwan	6
Prevalence	Southeast Asia, East Asia, and Oceania	Southeast Asia	Malaysia	1
Prevalence	Southeast Asia, East Asia, and Oceania	Southeast Asia	Thailand	2
Prevalence	Central Europe, Eastern Europe, and Central Asia	Central Asia	Georgia	1
Prevalence	Central Europe, Eastern Europe, and Central Asia	Central Asia	Mongolia	1
Prevalence	Central Europe, Eastern Europe, and Central Asia	Central Europe	Croatia	4
Prevalence	Central Europe, Eastern Europe, and Central Asia	Eastern Europe	Lithuania	3
Prevalence	Central Europe, Eastern Europe, and Central Asia	Eastern Europe	Russian Federation	2
Prevalence	High-income	High-income Asia Pacific	Japan	3
Prevalence	High-income	High-income Asia Pacific	Republic of Korea	5
Prevalence	High-income	High-income Asia Pacific	Singapore	1
Prevalence	High-income	Australasia	New Zealand	1
Prevalence	High-income	Western Europe	Austria	3
Prevalence	High-income	Western Europe	Belgium	1
Prevalence	High-income	Western Europe	Denmark	3
Prevalence	High-income	Western Europe	France	4
Prevalence	High-income	Western Europe	Germany	10

Prevalence	High-income	Western Europe	Greece	1
Prevalence	High-income	Western Europe	Ireland	1
Prevalence	High-income	Western Europe	Italy	6
Prevalence	High-income	Western Europe	Luxembourg	2
Prevalence	High-income	Western Europe	Netherlands	5
Prevalence	High-income	Western Europe	Norway	10
Prevalence	High-income	Western Europe	Spain	7
Prevalence	High-income	Western Europe	Sweden	3
Prevalence	High-income	Western Europe	Switzerland	1
Prevalence	High-income	Western Europe	United Kingdom	3
Prevalence	High-income	Southern Latin America	Argentina	1
Prevalence	High-income	Southern Latin America	Chile	1
Prevalence	High-income	High-income North America	Canada	2
Prevalence	High-income	High-income North America	United States of America	9
Prevalence	Latin America and Caribbean		Latin America and Caribbean	1
Prevalence	Latin America and Caribbean	Andean Latin America	Ecuador	2
Prevalence	Latin America and Caribbean	Andean Latin America	Peru	3
Prevalence	Latin America and Caribbean	Central Latin America	Colombia	1
Prevalence	Latin America and Caribbean	Central Latin America	Mexico	1
Prevalence	Latin America and Caribbean	Central Latin America	Venezuela (Bolivarian Republic of)	1
Prevalence	Latin America and Caribbean	Tropical Latin America	Brazil	9
Prevalence	North Africa and Middle East	North Africa and Middle East	Egypt	2
Prevalence	North Africa and Middle East	North Africa and Middle East	Iran (Islamic Republic of)	5
Prevalence	North Africa and Middle East	North Africa and Middle East	Kuwait	1
Prevalence	North Africa and Middle East	North Africa and Middle East	Morocco	2
Prevalence	North Africa and Middle East	North Africa and Middle East	Saudi Arabia	2
Prevalence	North Africa and Middle East	North Africa and Middle East	Türkiye	8
Prevalence	North Africa and Middle East	North Africa and Middle East	United Arab Emirates	1
Prevalence	South Asia	South Asia	India	4
Prevalence	South Asia	South Asia	Nepal	2
Prevalence	South Asia	South Asia	Pakistan	3
Prevalence	Sub-Saharan Africa	Eastern Sub-Saharan Africa	Ethiopia	3
Prevalence	Sub-Saharan Africa	Eastern Sub-Saharan Africa	United Republic of Tanzania	2
Prevalence	Sub-Saharan Africa	Eastern Sub-Saharan Africa	Zambia	2
Prevalence	Sub-Saharan Africa	Western Sub-Saharan Africa	Benin	1
Prevalence	Sub-Saharan Africa	Western Sub-Saharan Africa	Cameroon	1
Prevalence	Sub-Saharan Africa	Western Sub-Saharan Africa	Nigeria	3
Incidence	High-income	Western Europe	Germany	1
Incidence	High-income	Western Europe	Netherlands	1
Incidence	High-income	High-income North America	United States of America	1

Incidence	North Africa and Middle East	North Africa and Middle East	Türkiye	1
Remission	Central Europe, Eastern Europe, and Central Asia	Central Europe	Serbia	1
Remission	High-income	Western Europe	Austria	2
Remission	High-income	Western Europe	Germany	2
Remission	High-income	Western Europe	Norway	1
Remission	High-income	High-income North America	United States of America	1
		**Tension-type headache**		
Prevalence	Southeast Asia, East Asia, and Oceania	East Asia	China	5
Prevalence	Southeast Asia, East Asia, and Oceania	East Asia	Taiwan	3
Prevalence	Southeast Asia, East Asia, and Oceania	Southeast Asia	Malaysia	1
Prevalence	Central Europe, Eastern Europe, and Central Asia	Central Asia	Georgia	1
Prevalence	Central Europe, Eastern Europe, and Central Asia	Central Asia	Mongolia	1
Prevalence	Central Europe, Eastern Europe, and Central Asia	Central Europe	Croatia	3
Prevalence	Central Europe, Eastern Europe, and Central Asia	Eastern Europe	Lithuania	3
Prevalence	Central Europe, Eastern Europe, and Central Asia	Eastern Europe	Russian Federation	3
Prevalence	High-income	High-income Asia Pacific	Japan	3
Prevalence	High-income	High-income Asia Pacific	Republic of Korea	5
Prevalence	High-income	High-income Asia Pacific	Singapore	1
Prevalence	High-income	Australasia	New Zealand	1
Prevalence	High-income	Western Europe	Austria	2
Prevalence	High-income	Western Europe	Denmark	3
Prevalence	High-income	Western Europe	France	2
Prevalence	High-income	Western Europe	Germany	8
Prevalence	High-income	Western Europe	Ireland	1
Prevalence	High-income	Western Europe	Italy	6
Prevalence	High-income	Western Europe	Luxembourg	2
Prevalence	High-income	Western Europe	Netherlands	4
Prevalence	High-income	Western Europe	Norway	8
Prevalence	High-income	Western Europe	Spain	6
Prevalence	High-income	Western Europe	Sweden	2
Prevalence	High-income	Western Europe	Switzerland	1
Prevalence	High-income	Western Europe	United Kingdom	1
Prevalence	High-income	Southern Latin America	Chile	1
Prevalence	High-income	High-income North America	United States of America	1
Prevalence	Latin America and Caribbean		Latin America and Caribbean	1
Prevalence	Latin America and Caribbean	Andean Latin America	Ecuador	1
Prevalence	Latin America and Caribbean	Andean Latin America	Peru	2
Prevalence	Latin America and Caribbean	Tropical Latin America	Brazil	8
Prevalence	North Africa and Middle East	North Africa and Middle East	Egypt	1
Prevalence	North Africa and Middle East	North Africa and Middle East	Iran (Islamic Republic of)	3

Prevalence	North Africa and Middle East	North Africa and Middle East	Kuwait	1
Prevalence	North Africa and Middle East	North Africa and Middle East	Morocco	2
Prevalence	North Africa and Middle East	North Africa and Middle East	Saudi Arabia	2
Prevalence	North Africa and Middle East	North Africa and Middle East	Türkiye	4
Prevalence	South Asia	South Asia	India	3
Prevalence	South Asia	South Asia	Nepal	2
Prevalence	South Asia	South Asia	Pakistan	3
Prevalence	Sub-Saharan Africa	Eastern Sub-Saharan Africa	Ethiopia	3
Prevalence	Sub-Saharan Africa	Eastern Sub-Saharan Africa	Zambia	2
Prevalence	Sub-Saharan Africa	Western Sub-Saharan Africa	Benin	1
Prevalence	Sub-Saharan Africa	Western Sub-Saharan Africa	Cameroon	1
Prevalence	Sub-Saharan Africa	Western Sub-Saharan Africa	Nigeria	1
Remission	Central Europe, Eastern Europe, and Central Asia	Central Europe	Serbia	1
Remission	High-income	Western Europe	Austria	2
Remission	High-income	Western Europe	Germany	2
Remission	High-income	Western Europe	Norway	1
		**Medication overuse headache**		
Prevalence	Southeast Asia, East Asia, and Oceania	East Asia	China	1
Prevalence	Southeast Asia, East Asia, and Oceania	East Asia	Taiwan	3
Prevalence	Central Europe, Eastern Europe, and Central Asia	Central Asia	Georgia	1
Prevalence	Central Europe, Eastern Europe, and Central Asia	Eastern Europe	Lithuania	2
Prevalence	Central Europe, Eastern Europe, and Central Asia	Eastern Europe	Russian Federation	1
Prevalence	High-income	High-income Asia Pacific	Republic of Korea	1
Prevalence	High-income	Western Europe	Austria	1
Prevalence	High-income	Western Europe	Denmark	2
Prevalence	High-income	Western Europe	France	1
Prevalence	High-income	Western Europe	Germany	2
Prevalence	High-income	Western Europe	Italy	3
Prevalence	High-income	Western Europe	Luxembourg	1
Prevalence	High-income	Western Europe	Netherlands	2
Prevalence	High-income	Western Europe	Norway	4
Prevalence	High-income	Western Europe	Spain	4
Prevalence	High-income	Western Europe	Sweden	2
Prevalence	Latin America and Caribbean	Tropical Latin America	Brazil	1
Prevalence	North Africa and Middle East	North Africa and Middle East	Iran (Islamic Republic of)	1
Prevalence	North Africa and Middle East	North Africa and Middle East	Kuwait	1
Prevalence	North Africa and Middle East	North Africa and Middle East	Türkiye	1
Prevalence	South Asia	South Asia	India	1
Prevalence	South Asia	South Asia	Nepal	2
Prevalence	South Asia	South Asia	Pakistan	1

Prevalence	Sub-Saharan Africa	Eastern Sub-Saharan Africa	Ethiopia	1
Prevalence	Sub-Saharan Africa	Eastern Sub-Saharan Africa	Zambia	1
Remission	Central Europe, Eastern Europe, and Central Asia	Central Europe	Serbia	1
Remission	High-income	Western Europe	Austria	2
Remission	High-income	Western Europe	Germany	2
Remission	High-income	Western Europe	Norway	1

4.3. Data standardization

4.3.1. Age and sex splitting

Reported estimates of prevalence were split by age and sex where possible. First, if studies reported prevalence for broad age groups by sex (eg, prevalence in 15- to 65-year-old males and females separately), and also by specific age groups but for both sexes combined (eg, prevalence in 15- to 30-year-olds, then in 31- to 65-year-olds, for males and females combined), age-specific estimates were split by sex using the reported sex ratio and bounds of uncertainty. Second, prevalence data for both sexes that could not be split using a within-study ratio were split using a sex ratio derived from a meta-analysis of existing sex-specific data using a MR-BRT (meta-regression—Bayesian, regularised, trimmed) model [7]. The female to male ratio was 1.90 (1.85 to 1.96). Finally, after the application of bias adjustments, if studies reported estimates across age groups spanning 25 years or more, these were split into five-year age groups using the prevalence age pattern estimated by the best DisMod-MR 2.16 (disease model—Bayesian meta-regression) for each headache type.

4.3.2. Study quality and bias adjustment

We used a list of binary adjustment criteria which are a modified version of quality indicators of epidemiological studies on headache [8] and shown in the table below.

Supplementary table 2. Study-specific quality and bias covariate definitions

Study covariate	Notation	
	**Less desirable (1)**	**Reference (zero)**
**Other than one-year recall period**	Point prevalence	One-year prevalence
**Not representative**	Selected population	General population or community-based sample from whole country OR general population or community-based sample from defined region within a country, or school-based (for children)
**Low-quality sampling method**	Not stated OR no (or failed) attempt to secure representativeness	Total defined population, or random sample corrected for population demographics OR random sample uncorrected for population demographics
**Poor response**	Not stated, or <70%	70–100%
**Low-quality survey method and type of interviewer**	Not stated OR self-administered (unsupervised) questionnaire OR telephone or face-to-face interview by untrained or unspecified interviewer(s)	Face-to-face interview with headache expert or trained interviewer
**Low-quality validation of diagnostic instrument**	Instrument not specified or not validated OR validated, but sensitivity and/or specificity <70% OR validated only in screen-positive sub-sample, or in clinic or unspecified sample, but sensitivity and specificity 70%	Validated in target population or similar, and sensitivity and specificity 70%, or all diagnoses made in face-to-face or telephone interviews by headache expert
**Low-quality diagnostic criteria**	Not stated OR stated, other than ICHD OR ICHD (or reasonable modification)	ICHD (or reasonable modification)
**Headache type assumed**	Probable/definite headache has been assumed based on descriptions and not stated explicitly	Didn't have to assume headache type

Studies based on lifetime recall of headaches were not included because of the concern of significant recall bias. For migraine and TTH, we additionally tagged studies where the type of headache (probable/definite) was not explicitly

mentioned in the report but the type was determined based on the diagnostic criteria stated to the best of our understanding. This covariate is called “Headache type assumed”.

The mean and standard error for the coefficients were calculated using MR-BRT adjustment method. All study covariates were initially evaluated independently for each of the three types of headache. However, covariate values varied not only in magnitude but in direction across the three headache types. Because we assume that the same study covariate should adjust data at least in the same direction for all headache types, the final study covariates were evaluated taking all migraine, TTH, and MOH data into account. Studies conducted in a school setting were not adjusted for, as we were unable to find matches to inform a reliable crosswalk. These studies were not excluded because the headache models are relatively data sparse. Betas and inverse-logit values for these covariates are shown in the table below:

Supplementary table 3. MR-BRT Crosswalk Adjustment Factors for Headaches

**Data input**	**Reference or alternative case definition**	**Gamma**	**Beta Coefficient, Logit (95% UI)***	**Adjustment factor****
Other than one-year recall	Alt	1.20	-0.89 (-0.97 to -0.80)	0.30 (0.28 to 0.31)
Not representative	Alt		-0.39 (-0.45 to -0.33)	0.40 (0.39 to 0.42)
Low-quality sampling method	Alt		0.73 (0.66 to 0.79)	0.67 (0.66 to 0.69)
Poor response	Alt		-0.45 (-0.53 to -0.36)	0.40 (0.37 to 0.41)
Low-quality survey method	Alt		-0.22 (-0.31 to -0.13)	0.45 (0.42 to 0.47)
Low-quality diagnostic instrument	Alt		0.15 (0.13 to 0.19)	0.54 (0.53 to 0.55)
Low-quality diagnostic criteria	Alt		-0.37 (-0.43 to -0.32)	0.41 (0.39 to 0.42)
Headache type assumed	Alt		0.37 (0.33 to 0.42)	0.59 (0.58 to 0.60)

**MR-BRT crosswalk adjustments can be interpreted as the factor the alternative case definition is adjusted by to reflect what it would have been had it been measured using the reference case definition. If the log/logit beta coefficient is negative, then the alternative is adjusted up to the reference. If the log/logit beta coefficient is positive, then the alternative is adjusted down to the reference.*

***The adjustment factor column is the exponentiated beta coefficient. For log beta coefficients, this is the relative rate between the two case definitions. For logit beta coefficients, this is the relative odds between the two case definitions.*

4.4. Total Headache Model Data Adjustment

Because some data sources, especially earlier data from before ICHD became the standard (the initial criteria were published in 1988), largely report on definite migraine or TTH, we adjusted studies that reported only on definite headache type to the total headache type in order to better inform the total headache model. All data that reported on both definite and total migraine/TTH were used in regression models in order to derive an age- and sex-specific adjustment. This approach is consistent with GBD 2019 and 2021. The adjustment is shown in the graphs below.

Supplementary figure 5. Definite migraine to total migraine adjustment

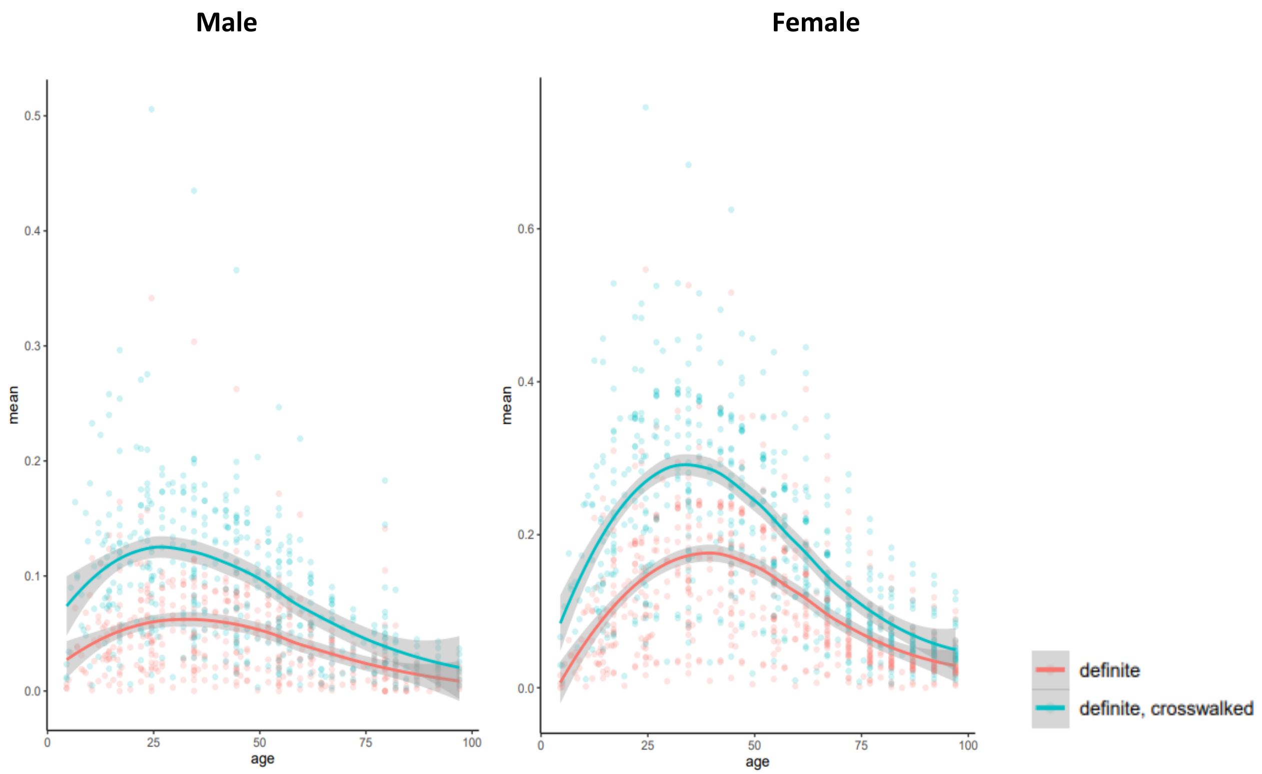

Supplementary figure 6. Definite TTH to total TTH adjustment

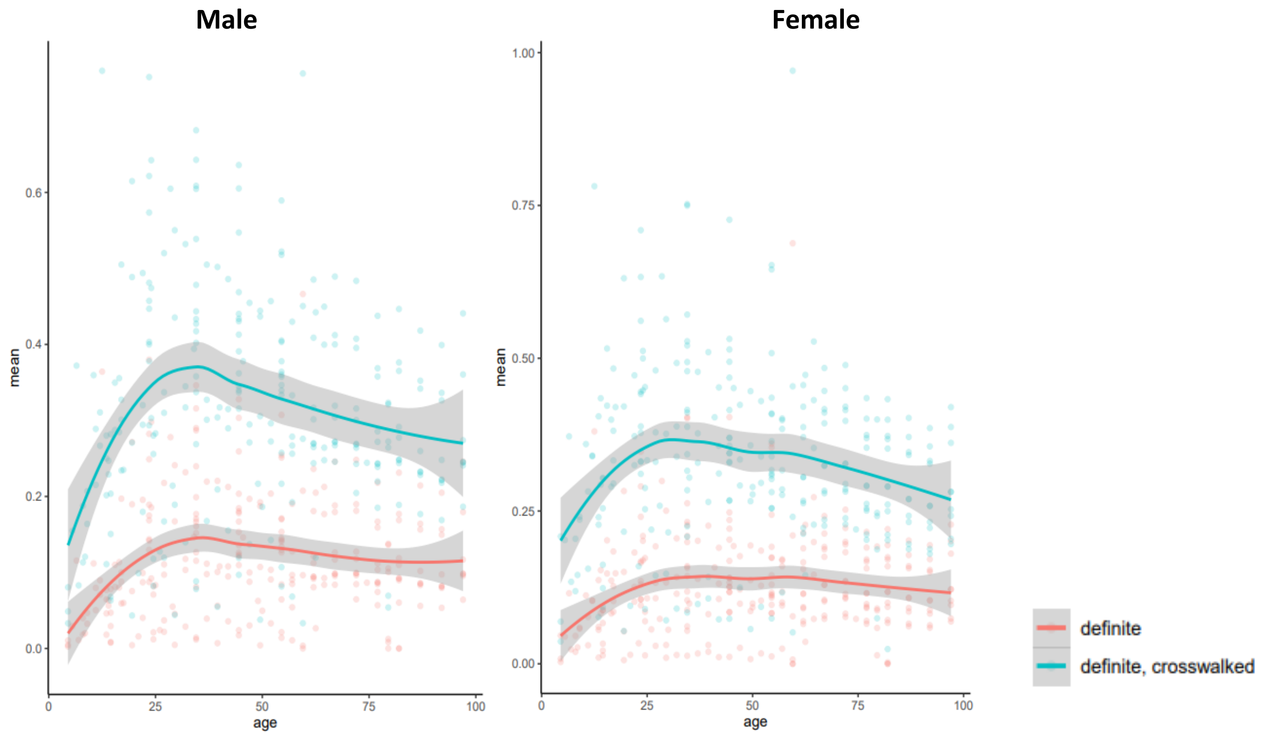

5. **Modelling strategy**

In GBD 2023, we made one significant update to the time-symptomatic analysis to better capture differences in headache frequency and duration by age and sex (described below). This is a substantial update compared to previous GBD rounds.

However, the remainder of the modelling strategy has largely remained the same compared to GBD 2019 and 2021 where standard DisMod-MR settings across all headache models included setting excess mortality to 0, and assuming that there was no incidence or prevalence before the age of 5 years. No covariates are used in any of the DisMod-MR models.

5.1. **Migraine**

We made no substantive changes in the modelling strategy of migraine from GBD 2019 and 2021. We continue to run separate DisMod-MR models for definite migraine, probable migraine, and the total migraine category and set an upper bound on remission of 0.1 across all models. After running the separate models, we then scaled the results of probable and definite headache to the total migraine envelope to ensure consistency.

5.2. **Tension-type headache**

We made no substantive changes in the modelling strategy of TTH from GBD 2019 and 2021. We ran separate DisMod-MR models for definite TTH, probable TTH, and the total TTH category, setting an upper bound on remission of 0.5 across all models. After running the separate models, we then scaled the results of probable and definite headache to the total headache envelope to ensure consistency.

5.3. **Medication overuse headache**

We made no substantive changes in the modelling strategy of MOH from GBD 2017 and 2021. Prior settings in the DisMod-MR model included an upper bound on remission of 0.4.

As MOH can develop from migraine or TTH, we split MOH burden into sequelae of migraine and TTH. Based on a 2017 meta-analysis of three sources, 73.2% (63.7–81.0) of MOH burden is assigned to MOH due to migraine, the remainder to MOH due to TTH. The forest plot is shown below.

Supplementary figure 7. Meta-analysis informing medication overuse headache split into migraine or tension-type headache

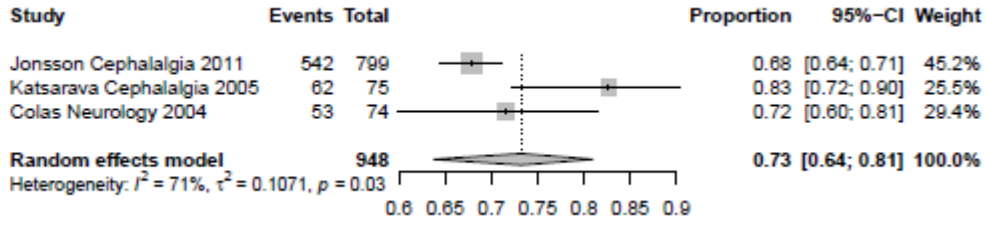

6. **Time Symptomatic Analysis**

6.1. **Input data time symptomatic**

Starting from GBD 2023, we have updated our time-symptomatic analysis for migraine and TTH using multi-country survey unit-record data from 18 countries, with data from at least one country in each GBD super region (see Supplementary table 3), in the Lifting The Burden survey series provided by our collaborators. Prior to GBD 2023, data from 19 countries (N=29,062), also part of the Lifting The Burden series, were used for this analysis. Importantly, three of these studies included participants from clinics and three others participants from patient organizations. In addition, the previous meta-analysis was based on aggregate data and the estimates were neither

age- nor sex-specific. In this updated analysis, we pooled only high-quality population-representative individual participant data from 18 countries, which resulted in 41,653 individual records. The age span of the participants were 18-65 years, the same as in previous GBD rounds.

Supplementary table 4. Time symptomatic input data

GBD super region	Country	Sample size
South-East Asia, East Asia & Oceania	China	5,041
Sub-Saharan Africa	Benin	2,400
	Cameroon	3,100
	Ethiopia	2,385
	Mali	2,105
	Zambia	1,085
South Asia	India	2,329
	Nepal	2,100
	Pakistan	4,223
Latin America & Caribbean	Peru	2,149
North Africa & Middle East	Morocco	2,575
	Saudi Arabia	2,316
Central Europe, Eastern Europe & Central Asia	Mongolia	2,041
	Russia	2,025
High Income	Lithuania	572
	Luxembourg	1,825
	Netherlands	2,414
	Spain	968

In the entire sample (N=41,653), 5,235 fulfilled the criteria for definite migraine, 4,953 the criteria for probable migraine, 10,442 the criteria for definite TTH, and 2,538 the criteria for probable TTH.

6.2. Mathematical calculation

Time in symptomatic state was calculated at individual level as a proportion by multiplying headache frequency with headache duration and dividing by total time available to have headache (range: 0-1).

$$Time\ in\ symptomatic\ state\ = (Headache\ frequency\ *\ Headache\ duration)/Total\ time\ available$$

However, since headache frequency was reported as days per time unit (days/month or days/year), as opposed to attacks per time unit, correction had to be made to avoid overestimation of time in symptomatic state. This was done by capping duration at 24 hours, i.e. one individual reporting a frequency of 4 days/month and a duration of 30 hours, would end up with the same time in symptomatic state ([4*24]/[30*24]) as someone reporting a frequency of

4 days/month and a duration of 24 hours. If duration had not been capped at 24 hours, 99 individuals would have ended up with a time in symptomatic state >100%, which is impossible.

6.3. Time symptomatic estimates

Proportion of time in symptomatic estimates can be interpreted as the average percentage of time in a year a person is actively experiencing a specific headache based on the case definitions. The updated proportions are shown below.

Supplementary table 5. Proportion of time symptomatic by age, sex and headache type

	Definite Migraine (Standard error)	Probable Migraine (Standard error)	Definite TTH (Standard error)	Probable TTH (Standard error)
Males				
5-35 years	0.0634 (0.0044)	0.0360 (0.0034)	0.0261 (0.0016)	0.0139 (0.0024)
35-49 years	0.0786 (0.0057)	0.0336 (0.0030)	0.0248 (0.0018)	0.0099 (0.0012)
50 and above	0.0867 (0.0087)	0.0604 (0.0078)	0.0355 (0.0029)	0.0244 (0.0058)
Females				
5-35 years	0.0930 (0.0039)	0.0565 (0.0035)	0.0398 (0.0021)	0.0188 (0.0023)
35-49 years	0.1013 (0.0043)	0.0519 (0.0033)	0.0359 (0.0022)	0.0215 (0.0037)
50 and above	0.1277 (0.0071)	0.0716 (0.0056)	0.0456 (0.0033)	0.0356 (0.0085)

For MOH no updates were made this round. We continue to use proportion of time symptomatic of 0.532, across age and sex, based on meta-analyzed results from two available studies on frequency and used the one available study on duration. Proportions are then applied to prevalence of each headache respectively to produce symptomatic prevalence of each headache type.

Supplementary table 6. Medication overuse headache: combined proportion split and time symptomatic proportion

Target	Mean proportion	lower	upper
Asymptomatic: MOH due to migraine	0.342	0.239	0.445
Symptomatic: MOH due to migraine	0.389	0.284	0.494
Asymptomatic: MOH due to TTH	0.126	0.056	0.196
Symptomatic: MOH due to TTH	0.143	0.070	0.216

7. Headache Severity Distribution

Supplementary table 7. Severity distribution, details on the severity levels for headaches and the associated disability weight (DW) with that severity.

Severity level	Lay description	DW (95% CI)
Symptomatic probable migraine	has severe, throbbing head pain and nausea that cause great difficulty in daily activities and sometimes confine the person to bed. Moving around, light, and noise make it worse.	0.441 (0.294-0.588)
Symptomatic definite migraine	has severe, throbbing head pain and nausea that cause great difficulty in daily activities and sometimes confine the person to bed. Moving around, light, and noise make it worse.	0.441 (0.294-0.588)

Symptomatic medication overuse headache due to migraine	has daily headaches, felt as dull pain and often lasting all day, with poor sleep, nausea and fatigue. The person takes medicine for the headaches, which provides little relief but is needed to avoid having worse symptoms.	0.223 (0.146-0.313)
Symptomatic probable tension-type headache	has a moderate headache that also affects the neck, which causes difficulty in daily activities.	0.037 (0.022-0.057)
Symptomatic definite tension-type headache	has a moderate headache that also affects the neck, which causes difficulty in daily activities.	0.037 (0.022-0.057)
Symptomatic medication overuse headache due to tension-type headache	has daily headaches, felt as dull pain and often lasting all day, with poor sleep, nausea and fatigue. The person takes medicine for the headaches, which provides little relief but is needed to avoid having worse symptoms.	0.223 (0.146-0.313)

8. Supplementary results

8.1. Country-specific prevalence and YLD estimates

Supplementary tables 8 and 9 show country-specific age-standardized prevalence and YLD rates (per 100,000 persons) attributed to migraine and tension-type headache respectively. As primary input data on prevalence are lacking from many of these countries, we caution against drawing overly confident conclusions based on these estimates.

Supplementary table 8. Country-specific age-standardized prevalence and years lived with disability (YLD) rates (per 100,000 persons) attributed to migraine

Country	Prevalence per 100,000 persons (95% UI)		YLDs per 100,000 persons (95% UI)	
	1990	2023	1990	2023
Afghanistan	15466 (13170–17986)	15057 (12797–17473)	547·8 (370–747·5)	536·8 (355–740·2)
Albania	13261 (11205–15480)	13435 (11344–15678)	458·1 (305·3–631·1)	466·5 (309·6–640·3)
Algeria	15037 (12780–17468)	15027 (12772–17467)	553·3 (367·5–760·4)	553·1 (371·7–753·3)
Andorra	16891 (14269–19567)	16980 (14348–19678)	585·4 (384–814·6)	585·2 (388·7–818·8)
Angola	12957 (10922–15231)	13138 (11085–15436)	440·5 (291·9–607·1)	453·3 (297·9–627·7)
Antigua and Barbuda	13676 (11335–15798)	13587 (11258–15705)	477·1 (322·5–663·2)	470·7 (314·8–654·8)
Argentina	10863 (9245–12556)	11278 (9612–13067)	379·2 (250·3–521·1)	392·5 (261–547·5)
Armenia	13392 (11377–15559)	13410 (11389–15564)	459·3 (300·3–634·4)	459·4 (301·5–633)
Australia	13344 (11235–15709)	13365 (11259–15729)	449·6 (299–621·6)	447·7 (297·9–612·5)
Austria	16248 (13698–18736)	16174 (13628–18632)	569·8 (369–783·9)	561·9 (368·3–776·1)
Azerbaijan	13428 (11410–15604)	13298 (11298–15453)	460·1 (300·9–639·9)	454·2 (298·3–619·8)
Bahamas	13642 (11303–15764)	13656 (11317–15778)	475·4 (322–658·4)	473·7 (315·9–661·5)
Bahrain	14176 (11991–16507)	13878 (11739–16159)	510·2 (339·8–701·6)	493·8 (331·9–682·3)
Bangladesh	14395 (12240–16659)	14559 (12391–16845)	472·3 (311·2–653·3)	485·8 (317·8–667·5)
Barbados	13667 (11324–15786)	13614 (11279–15732)	479 (327–666·6)	473·8 (312·7–659·8)
Belarus	12903 (10842–15039)	12867 (10820–15004)	487·1 (321·1–667·8)	485 (319–659·7)

Belgium	20053<br>(17135–23369)	20064<br>(17144–23379)	702<br>(456·8–974·7)	696·7<br>(456·2–960·3)
Belize	13439<br>(11123–15565)	13627<br>(11294–15754)	465·9<br>(315–646·1)	472<br>(316·5–653·2)
Benin	15443<br>(12976–17977)	15344<br>(12879–17862)	529·6<br>(350·6–725·7)	527·1<br>(345·4–727·8)
Bhutan	14329<br>(12182–16602)	14406<br>(12252–16676)	472·9<br>(309·9–658·9)	478·2<br>(318·6–667·3)
Bolivia (Plurinational State of)	10667<br>(9007–12561)	10615<br>(8960–12492)	372·4<br>(243·6–512·2)	369<br>(247·2–507·1)
Bosnia and Herzegovina	13410<br>(11323–15637)	13406<br>(11321–15637)	465·4<br>(306–640)	463<br>(308·7–633·1)
Botswana	13160<br>(11102–15466)	13070<br>(11028–15358)	457·8<br>(300·2–642)	450·3<br>(297·7–620·8)
Brazil	17728<br>(15277–20123)	18151<br>(15472–20733)	603<br>(414–826·7)	613·1<br>(415·4–834·4)
Brunei Darussalam	9966<br>(8401–11707)	9990<br>(8422–11724)	342·1<br>(228–474·9)	344·1<br>(230·2–472·4)
Bulgaria	13439<br>(11348–15676)	13335<br>(11263–15556)	466·7<br>(309·2–638·8)	462<br>(307·3–638·2)
Burkina Faso	15457<br>(12986–17991)	15394<br>(12927–17916)	529·3<br>(347·6–735·7)	530·8<br>(351·6–731·6)
Burundi	9984<br>(8494–11599)	9814<br>(8330–11439)	358·2<br>(234·8–496·8)	347·6<br>(229·2–481·6)
Cabo Verde	15577<br>(13100–18101)	15226<br>(12771–17732)	544·5<br>(359·5–753·8)	522·9<br>(346·5–718·4)
Cambodia	15819<br>(13387–18173)	15601<br>(13179–17944)	558·5<br>(368·8–771·9)	552<br>(361·2–770·3)
Cameroon	15345<br>(12883–17869)	15275<br>(12817–17795)	522·5<br>(346·5–715·7)	521·7<br>(348·1–721·7)
Canada	16489<br>(14424–18746)	16104<br>(13572–18736)	583·4<br>(377·9–801·4)	565·7<br>(374·7–785·7)
Central African Republic	13084<br>(11037–15374)	13051<br>(11003–15345)	444·1<br>(292–606·9)	442·3<br>(293·4–613·4)
Chad	15418<br>(12952–17942)	15283<br>(12824–17815)	529·7<br>(347–733·5)	521<br>(345·9–721·4)
Chile	11842<br>(10073–13751)	12013<br>(10080–13896)	414·1<br>(272·1–576·7)	416·5<br>(273·9–581·2)
China	10676<br>(9237–12256)	11420<br>(9841–12927)	372·6<br>(252·1–525·6)	399·9<br>(266·1–554·2)
Colombia	13662<br>(11612–15532)	13731<br>(11556–15930)	482·6<br>(317·1–663·9)	485·1<br>(323·4–666·4)
Comoros	9923<br>(8436–11535)	9852<br>(8369–11467)	354·7<br>(233·9–495·1)	352·4<br>(231·8–482·8)
Congo	13090<br>(11045–15379)	13006<br>(10962–15293)	449·9<br>(294·4–625·9)	445·2<br>(292·2–616·4)
Costa Rica	13361<br>(11312–15465)	13305<br>(11259–15411)	470·1<br>(319·1–654)	465·2<br>(310·6–646·9)
Côte d'Ivoire	15139<br>(12691–17657)	15084<br>(12638–17602)	506·9<br>(337·9–691·3)	509·9<br>(335·8–701·5)
Croatia	13449<br>(11354–15680)	13314<br>(11287–15421)	466·5<br>(314·4–642·1)	462·1<br>(305·6–637·1)
Cuba	13507<br>(11184–15624)	13470<br>(11150–15586)	469·3<br>(310·9–637·5)	467·5<br>(313·5–649·6)
Cyprus	17199<br>(14540–19942)	17301<br>(14627–20071)	600·6<br>(395·4–835·9)	603·1<br>(401·2–837·8)

Czechia	13456 (11361–15689)	13347 (11275–15572)	465·5 (308·1–640)	459·9 (306·6–633·9)
Democratic People's Republic of Korea	11665 (9839–13536)	11594 (9776–13461)	409·4 (274·7–569·2)	403·4 (273·3–558·7)
Democratic Republic of the Congo	13087 (11045–15374)	12987 (10952–15261)	444·6 (294·9–607·2)	444·2 (294·4–618·4)
Denmark	14747 (12658–17258)	15221 (12833–17562)	521·7 (351·5–705·9)	534·5 (351·8–736·8)
Djibouti	9755 (8277–11370)	9694 (8221–11303)	347·8 (228·8–485·1)	343·5 (225·7–473·7)
Dominica	13556 (11222–15671)	13410 (11099–15527)	471·9 (316·3–647·4)	461·3 (307·4–640·8)
Dominican Republic	13630 (11297–15763)	13553 (11225–15680)	474·1 (317·2–657·9)	468·8 (312·7–646·9)
Ecuador	11765 (10009–13564)	12103 (10231–14026)	411·6 (268·5–563·7)	422·5 (278·3–583·4)
Egypt	15261 (13010–17508)	15613 (13825–17392)	559·6 (373·1–771)	570 (374–772·1)
El Salvador	13548 (11467–15650)	13749 (11624–15841)	478·1 (318·5–657·2)	488·7 (331·3–670·6)
Equatorial Guinea	13208 (11144–15522)	12931 (10913–15169)	455·2 (296·8–622·4)	443·3 (290·3–614·8)
Eritrea	9990 (8501–11599)	9815 (8334–11426)	353·1 (231·8–485·9)	347·3 (225·4–479·8)
Estonia	12893 (10844–15024)	12704 (10669–14833)	485·4 (323·5–661)	476·2 (314·9–649·1)
Eswatini	13200 (11132–15516)	13053 (11015–15331)	463·9 (309·9–643·5)	446·9 (297·6–617·8)
Ethiopia	9932 (8548–11388)	9666 (8348–11170)	346·3 (235–485)	338·3 (224·8–470·7)
Fiji	13638 (11462–15762)	13627 (11447–15747)	469·3 (318·5–649·1)	465·4 (314·7–643·9)
Finland	17253 (14579–20004)	17151 (14493–19881)	602·8 (392–837·2)	594·7 (392·1–823·4)
France	17080 (14517–19852)	16924 (14462–19477)	608·3 (404–850·6)	599·8 (392·2–825·6)
Gabon	13014 (10977–15290)	13091 (11038–15388)	446·4 (292·6–617·7)	449·8 (298·3–623·7)
Gambia	15196 (12737–17721)	15311 (12847–17833)	517·4 (345·9–720·1)	523·8 (346·3–720·5)
Georgia	13445 (11423–15614)	13285 (11285–15422)	461·8 (298·1–634·7)	452·2 (295·5–626·1)
Germany	18523 (15865–21312)	18456 (15785–21234)	642·1 (425·3–869·3)	636 (421·5–878·7)
Ghana	15324 (12860–17844)	15314 (12851–17825)	522·8 (346·2–722·7)	525·7 (346·3–724·2)
Greece	17913 (15175–20626)	17853 (15037–20853)	619·6 (408·1–857·1)	612·8 (403·1–848·4)
Grenada	13595 (11262–15718)	13386 (11076–15500)	472·5 (321·4–648·6)	460·9 (315–642·3)
Guatemala	13451 (11387–15569)	13563 (11478–15659)	467·9 (318–639·4)	474·5 (323·2–651·1)
Guinea	15394 (12927–17931)	15399 (12934–17935)	528·2 (349–730·9)	530 (354·3–735·1)
Guinea-Bissau	15414 (12950–17943)	15371 (12907–17892)	527·3 (348·5–724·8)	526·4 (349–730)

Guyana	13571 (11242–15697)	13570 (11240–15690)	466·4 (309·8–644·1)	467·5 (318·2–648·3)
Haiti	13649 (11311–15791)	13662 (11323–15782)	467·8 (313·6–635)	469·3 (317·4–647·8)
Honduras	13429 (11370–15536)	13557 (11472–15648)	472·8 (315·6–650·6)	478·5 (317·3–657·5)
Hungary	13496 (11396–15734)	13397 (11314–15624)	465·8 (308·2–637·5)	463·9 (308·2–640·4)
Iceland	17204 (14533–19944)	17031 (14389–19737)	600 (394·8–834·5)	585·8 (384·9–819·3)
India	14628 (12727–16670)	14619 (12625–16703)	486·3 (330·8–673·2)	490 (325·8–687·8)
Indonesia	15905 (13649–18010)	15817 (13582–17910)	559·7 (373·1–775·4)	557·4 (370·8–773·5)
Iran (Islamic Republic of)	15877 (13677–18067)	16265 (14104–18385)	577 (384·8–797·5)	594·1 (407·3–824·7)
Iraq	14923 (12675–17335)	14902 (12651–17312)	542·1 (358·8–752·5)	540 (361·3–739·4)
Ireland	17257 (14579–20008)	17319 (14632–20085)	603 (395–843·2)	601·2 (401·1–830·5)
Israel	17360 (14666–20136)	17234 (14564–19985)	609·9 (399·7–850·7)	600·5 (394·9–837·2)
Italy	18795 (16340–21371)	18871 (16364–21297)	662 (434·7–913·2)	663·1 (444·9–918·7)
Jamaica	13610 (11274–15737)	13561 (11233–15679)	474·7 (318·2–657·4)	470·4 (313·6–646·8)
Japan	10228 (8860–11759)	10498 (9156–12124)	358·5 (236·7–498·3)	364·8 (240·3–506·1)
Jordan	14840 (12603–17251)	14664 (12423–17044)	544 (364·2–757·3)	533·6 (355·7–738)
Kazakhstan	13405 (11390–15563)	13337 (11330–15491)	457·3 (302–637)	456·3 (297–626)
Kenya	10403 (9056–11849)	10404 (9056–11851)	374·2 (247·5–516·1)	374 (248·1–517)
Kiribati	13748 (11554–15870)	13758 (11559–15879)	474·3 (317·2–652·6)	474·9 (317·7–653·4)
Kuwait	13946 (11679–16089)	13673 (11629–15755)	498 (330·9–688·5)	481·1 (322·2–654·1)
Kyrgyzstan	13383 (11371–15547)	13353 (11344–15519)	456·5 (299·6–630·3)	457·7 (299·2–637·9)
Lao People's Democratic Republic	15619 (13202–17971)	15473 (13065–17820)	550·8 (372·9–764·9)	547·2 (356·2–764·7)
Latvia	12908 (10856–15041)	12761 (10725–14887)	485·2 (316·7–662·2)	479·4 (320–654·2)
Lebanon	15158 (12894–17631)	15055 (12806–17474)	553·7 (366·5–761·7)	552·1 (367·2–767·8)
Lesotho	13403 (11322–15737)	13085 (11042–15370)	475·9 (315·6–655·8)	448·2 (297·7–621·8)
Liberia	15205 (12750–17727)	15195 (12737–17709)	508·3 (338–697·3)	512·5 (340·3–704·1)
Libya	14657 (12416–17036)	14923 (12670–17343)	534·5 (355–735·4)	543·2 (360·4–735·8)
Lithuania	12233 (10419–14252)	12075 (10292–14076)	453·2 (299·9–622·3)	447 (297·7–618)
Luxembourg	16766 (14289–19469)	16703 (14239–19399)	590·5 (390·9–813)	584·5 (388·3–810·3)

Madagascar	9887 (8402–11499)	9912 (8425–11528)	352·3 (233·4–486·1)	355·5 (233·7–494·7)
Malawi	9936 (8448–11551)	9966 (8476–11584)	354·6 (235·8–489·1)	357 (232·5–488·2)
Malaysia	13602 (11510–15673)	13755 (11600–16013)	482·2 (319–656·7)	483·7 (319·8–668·8)
Maldives	15356 (12968–17713)	14707 (12342–16995)	535·2 (352·4–743·8)	503 (338·8–693·7)
Mali	15350 (12887–17881)	15256 (12801–17788)	521·9 (349·1–719·7)	518 (344·3–716)
Malta	17289 (14611–20051)	16824 (14215–19489)	606 (390·5–841·8)	578 (380·6–807·1)
Marshall Islands	13579 (11405–15699)	13608 (11442–15720)	467·1 (320·7–641·8)	465·9 (313–644·3)
Mauritania	15326 (12862–17844)	15343 (12882–17865)	526·7 (347·4–729·9)	528·6 (349·4–734·2)
Mauritius	15511 (13096–17854)	15476 (13066–17827)	545·4 (362–756)	540·7 (361·9–742·5)
Mexico	13488 (11586–15277)	13658 (11775–15427)	473·7 (319·5–650·3)	478·4 (324–662·9)
Micronesia (Federated States of)	13614 (11433–15739)	13618 (11441–15737)	470·4 (316·5–649·7)	466·3 (314·5–646·1)
Monaco	17348 (14659–20122)	17313 (14630–20082)	609·9 (399·6–852·9)	601·5 (394·3–829·1)
Mongolia	13297 (11298–15470)	13362 (11351–15520)	453·6 (296·5–631·5)	458·5 (299·8–631·6)
Montenegro	13413 (11327–15645)	13370 (11291–15601)	467·1 (308·1–648·3)	462·5 (306·3–636·4)
Morocco	15100 (12837–17550)	15019 (12764–17458)	555 (366·5–765·1)	552 (372–758·2)
Mozambique	10022 (8528–11644)	10007 (8515–11626)	356·2 (234·9–486·8)	354·6 (234·1–486·1)
Myanmar	15576 (13160–17926)	15642 (13219–17988)	545·7 (358·4–760·1)	553·6 (369·3–771·2)
Namibia	13090 (11041–15382)	13082 (11038–15368)	455·9 (299·7–632·1)	453·1 (303·2–624·8)
Nauru	13590 (11414–15704)	13677 (11487–15793)	467·4 (311·2–648·7)	470·3 (317·5–646·5)
Nepal	15505 (13060–17734)	15655 (13221–18264)	509·2 (336·8–696·5)	524·9 (350·6–728·6)
Netherlands	15864 (13549–18317)	16866 (14221–19635)	548·6 (360·7–765·1)	573·3 (380·8–784·2)
New Zealand	13867 (11940–16124)	13818 (11897–16061)	466·3 (314·7–655·5)	463·9 (313·3–648·1)
Nicaragua	13503 (11430–15610)	13442 (11373–15530)	476·4 (316·8–659·2)	472·8 (320·7–652·3)
Niger	15277 (12813–17798)	15327 (12866–17851)	521·3 (347–721·7)	526·4 (348–729·1)
Nigeria	15789 (13529–18142)	15959 (13769–18209)	533·1 (359·2–737·7)	551·4 (378·1–757·5)
North Macedonia	13405 (11320–15639)	13382 (11303–15614)	465·5 (310·5–637·9)	462·8 (308·9–636·6)
Norway	15977 (13774–18223)	16826 (14647–19202)	551·3 (367·9–763·4)	577·2 (389·8–797·8)
Oman	13891 (11749–16171)	13914 (11760–16203)	494·6 (331·1–681·3)	497·5 (329·7–688·5)

Pakistan	14448 (12494–16421)	14407 (12442–16369)	474·2 (315·5–659·8)	466 (311·6–640·1)
Palau	13571 (11404–15688)	13375 (11231–15480)	468·4 (313·4–650·7)	454·2 (302·6–629)
Palestine	15167 (12908–17610)	14990 (12738–17421)	557·4 (367·3–769·1)	547·7 (364·7–756·9)
Panama	13273 (11232–15382)	13372 (11318–15468)	469·6 (313·1–648·2)	471 (321·6–645·1)
Papua New Guinea	13553 (11393–15667)	13559 (11408–15668)	463·6 (311·7–640·6)	464·4 (314·4–642·2)
Paraguay	17037 (14298–19728)	17081 (14331–19770)	581·3 (387·5–797·4)	579·4 (399·1–793·5)
Peru	8535 (7346–9824)	9415 (7891–11056)	300·1 (202·2–409)	328·9 (221·8–451·6)
Philippines	15870 (13623–17972)	15823 (13586–17920)	559·1 (371·2–773·7)	558·9 (371–772·5)
Poland	13962 (12081–16030)	13897 (12023–15964)	485·5 (324·1–668·9)	484·4 (323·7–668·5)
Portugal	17393 (14693–20175)	17348 (14660–20123)	607·9 (400·4–847·3)	602·7 (393·7–834·9)
Qatar	13325 (11253–15617)	13458 (11360–15757)	467·5 (311·9–646·4)	474·3 (314·7–650·2)
Republic of Korea	12103 (10300–14249)	11539 (9692–13665)	407·1 (268·2–573·9)	391·7 (257·4–543·7)
Republic of Moldova	12939 (10879–15088)	12774 (10735–14904)	486·6 (320·5–658·7)	480·9 (317·5–657·5)
Romania	13444 (11351–15679)	13374 (11295–15599)	466·1 (308·4–641·9)	464·4 (309·7–642·2)
Russian Federation	14041 (12155–15948)	14003 (12116–15909)	534·1 (351·9–735)	532·3 (349·8–731·4)
Rwanda	9989 (8500–11602)	9984 (8495–11597)	358·8 (236·3–496·9)	358·9 (239·7–490·2)
Saint Kitts and Nevis	13606 (11270–15727)	13674 (11335–15801)	471·4 (318–647·1)	474·8 (317·2–661·6)
Saint Lucia	13659 (11318–15782)	13508 (11185–15627)	473·7 (312·9–655·9)	466·1 (310·8–645·8)
Saint Vincent and the Grenadines	13545 (11216–15666)	13441 (11128–15559)	469·5 (316·4–647·3)	462·2 (309·9–641·2)
Samoa	13545 (11394–15645)	13653 (11486–15761)	467·6 (312·3–646)	471·5 (312·9–651·1)
San Marino	17304 (14620–20064)	17242 (14581–19997)	606·1 (392·2–841)	597·9 (389·7–834·2)
Sao Tome and Principe	15344 (12879–17865)	15242 (12784–17753)	529·3 (348–722)	523·4 (351·2–729·8)
Saudi Arabia	13953 (11829–16234)	13678 (11595–15939)	497·8 (335·2–682·6)	482 (324–671·1)
Senegal	15348 (12885–17871)	15301 (12845–17831)	523·8 (345·4–726·4)	524 (346–718·7)
Serbia	13419 (11340–15648)	13282 (11216–15503)	467·7 (310·3–645·7)	460·7 (307·7–635·5)
Seychelles	15465 (13051–17812)	15061 (12674–17390)	547·6 (363·1–757·8)	522·4 (351–723)
Sierra Leone	15314 (12854–17845)	15263 (12801–17787)	520·2 (344·7–722·9)	517·2 (347–717·3)
Singapore	7969 (6873–9244)	9187 (7714–10831)	290·1 (193·1–400·5)	323·2 (211·9–455·7)

Slovakia	13479 (11380–15717)	13375 (11296–15600)	467·9 (311·7–642·7)	463·2 (305·5–634·9)
Slovenia	13452 (11355–15683)	13232 (11185–15440)	466 (303·6–649·7)	456·6 (304·2–633·3)
Solomon Islands	13530 (11378–15653)	13593 (11423–15715)	466·9 (309·2–639·4)	468·7 (315·7–647·7)
Somalia	9823 (8344–11425)	9863 (8380–11469)	345·2 (225·5–474·4)	350·2 (231–481·9)
South Africa	13606 (11846–15561)	13576 (11820–15529)	474·5 (318·3–659·7)	467·2 (314·4–648·6)
South Sudan	9686 (8211–11310)	9914 (8429–11523)	337·6 (221·3–468·2)	350·4 (233–479·9)
Spain	18399 (15686–21434)	18343 (15612–21402)	646·8 (422·4–895·7)	641·8 (422·1–886·8)
Sri Lanka	15488 (13081–17837)	15602 (13183–17948)	544·8 (361·9–760·6)	552·9 (366·4–770·5)
Sudan	15100 (12832–17569)	14968 (12708–17393)	551·7 (367·7–762·9)	547·7 (363·2–755·8)
Suriname	13513 (11193–15628)	13580 (11247–15697)	467 (316·3–648·9)	468·2 (313·8–642·5)
Sweden	15931 (13629–18372)	16067 (13695–18804)	547·1 (369·3–760·2)	544·9 (367·3–752·3)
Switzerland	15347 (13283–17641)	15519 (13238–18049)	535·3 (358·3–740·7)	537·5 (360·5–746)
Syrian Arab Republic	14975 (12721–17411)	15294 (13014–17803)	544·3 (362·7–755)	554·4 (371·1–761·5)
Taiwan	12121 (10554–13719)	12649 (10783–14586)	423·1 (289–590·3)	437·6 (291–591·7)
Tajikistan	13315 (11314–15487)	13247 (11252–15410)	455·7 (295·9–624·5)	451·3 (297·9–633·8)
Thailand	19154 (16628–21584)	17485 (14707–20226)	685·8 (453·4–949)	619·4 (402–857·6)
Timor-Leste	15394 (12992–17730)	15427 (13024–17773)	536·3 (359·9–744·7)	542·4 (357·6–749·4)
Togo	15425 (12956–17951)	15391 (12922–17911)	529·7 (349·6–728·9)	528·1 (349·2–731·2)
Tonga	13727 (11550–15842)	13863 (11654–15983)	476·4 (319·4–664·1)	481·1 (326–667·9)
Trinidad and Tobago	13520 (11196–15642)	13488 (11165–15608)	466·1 (315·6–640·5)	463·8 (308·4–635·6)
Tunisia	15045 (12788–17490)	15107 (12848–17560)	554·3 (370–770·8)	555·6 (370·4–768·7)
Türkiye	14828 (12659–17185)	14928 (12691–17067)	554 (364·8–756)	557·9 (370·2–766·4)
Turkmenistan	13378 (11367–15546)	13160 (11181–15296)	457·8 (298·7–628·1)	448·2 (294–613·9)
Tuvalu	13909 (11693–16034)	13566 (11395–15674)	484·1 (324·4–668·7)	464·8 (317·4–647·1)
Uganda	9916 (8428–11532)	9973 (8484–11586)	349·6 (229·4–487)	358·2 (237·3–493·5)
Ukraine	13433 (11701–15424)	13334 (11620–15308)	504·6 (332·8–691·8)	500·5 (336·8–691·7)
United Arab Emirates	13453 (11345–15761)	13503 (11369–15827)	472·1 (316·8–655·1)	473 (314·8–645·5)
United Kingdom	16821 (14553–19165)	16945 (14632–19195)	588·1 (391·6–808·7)	584·8 (386·8–808)

United Republic of Tanzania	9117 (7801–10503)	9534 (8127–11198)	328·5 (217·9–449·8)	344 (233·3–474·6)
United States of America	16796 (14687–19134)	16360 (14103–19044)	582·7 (386·7–805·7)	563·8 (379·4–782·4)
Uruguay	11675 (9766–13700)	11684 (9775–13708)	406·9 (268·3–562·5)	406·3 (266·5–559·6)
Uzbekistan	13351 (11345–15519)	13310 (11305–15467)	456·4 (298·9–632·4)	456·3 (294·1–632·9)
Vanuatu	13563 (11403–15685)	13675 (11497–15795)	466·6 (314·7–649·5)	473·9 (319·1–646·6)
Venezuela (Bolivarian Republic of)	13114 (11278–14949)	13569 (11359–15768)	462·1 (318·2–632·5)	477·7 (311·6–664·3)
Viet Nam	15692 (13264–18035)	15483 (13067–17818)	557·4 (367·3–775)	548·6 (363·3–765·2)
Yemen	15041 (12782–17493)	15063 (12804–17504)	542·7 (358·2–746·7)	543·6 (360·4–754·5)
Zambia	10533 (8928–12325)	10614 (9016–12428)	377 (247·9–522·4)	383 (255·4–537·1)
Zimbabwe	13069 (11017–15362)	13147 (11094–15448)	453·2 (296·8–631·5)	456·2 (303·4–635)

Supplementary table 9. Country-specific age-standardized prevalence and years lived with disability (YLD) rates (per 100,000 persons) attributed to tension-type headache

Country	Prevalence per 100,000 persons (95% UI)		YLDs per 100,000 persons (95% UI)	
	1990	2023	1990	2023
Afghanistan	23315 (19980–26684)	23418 (20112–26836)	63·7 (42–93·4)	64 (42·3–93·2)
Albania	30204 (26306–34701)	30244 (26359–34790)	70·6 (48–101·6)	71·9 (48–101·7)
Algeria	23373 (20058–26828)	23357 (20061–26764)	65·7 (42·9–95·3)	66·2 (42·8–97·2)
Andorra	32416 (28354–37361)	32429 (28396–37373)	68·3 (46·4–97·9)	68·9 (46·9–98·9)
Angola	23341 (19707–27046)	23324 (19711–26972)	51·4 (33·7–75·7)	52·6 (35·4–74·9)
Antigua and Barbuda	23775 (20641–27078)	23759 (20597–27160)	50 (33·5–70·8)	50·1 (33·3–70·1)
Argentina	25922 (22175–30026)	25925 (22154–29978)	58 (39–84·2)	58·4 (39–83·3)
Armenia	30867 (26699–35415)	30864 (26618–35449)	66·2 (44·7–94)	66·7 (44·6–94·2)
Australia	26452 (22927–30468)	26450 (22929–30471)	58 (39–83·6)	58 (38·7–82·9)
Austria	31716 (27633–36623)	31703 (27512–36625)	71·4 (48·1–101·3)	71 (48–101·3)
Azerbaijan	30871 (26683–35504)	30857 (26688–35477)	66·5 (45·5–93·2)	66·2 (44·3–93·5)
Bahamas	23778 (20649–27130)	23775 (20603–27212)	50·3 (34·1–71)	50·3 (34·1–70·8)
Bahrain	23476 (20151–26813)	23453 (20098–26890)	63·6 (41·8–92·2)	63 (40·9–91·7)
Bangladesh	25628 (22069–29212)	25576 (21964–29164)	47·5 (31·8–66·4)	48·9 (32·9–68·9)

Barbados	23776 (20613–27142)	23764 (20596–27164)	50·4 (34–70·7)	50·2 (33·7–70·9)
Belarus	31326 (27178–36075)	31326 (27132–35989)	86·5 (56·7–125·1)	86·6 (56·4–125)
Belgium	32474 (28441–37432)	32466 (28421–37396)	69·6 (47·2–98·5)	69·7 (47·4–100·2)
Belize	23760 (20550–27115)	23756 (20550–27117)	49 (33–69·7)	49·7 (33·6–69·8)
Benin	24616 (20888–28264)	24622 (20899–28249)	53·7 (35·8–78·6)	53·9 (35·9–77·6)
Bhutan	25629 (22065–29205)	25616 (22000–29212)	47·7 (31·7–67·9)	48·5 (32·7–68·7)
Bolivia (Plurinational State of)	20478 (17506–24068)	20459 (17480–23995)	45·9 (30·7–66·8)	46·1 (30·6–66)
Bosnia and Herzegovina	30254 (26381–34758)	30245 (26355–34718)	71·3 (48·2–100·5)	71·6 (48·7–102·1)
Botswana	23324 (19655–27010)	23329 (19649–26975)	53 (35·6–76·1)	52·8 (35·4–76·9)
Brazil	29034 (25756–32413)	29171 (25909–32860)	54·5 (37·6–77·8)	55 (37·8–77·4)
Brunei Darussalam	28902 (24851–33111)	28927 (24831–33082)	57·5 (38·4–82·6)	58·2 (39·4–82·6)
Bulgaria	30251 (26296–34698)	30229 (26339–34735)	71·5 (47·7–101)	71·7 (48·1–101·9)
Burkina Faso	24619 (20829–28288)	24622 (20857–28240)	53·5 (35·6–76·5)	54 (35·6–78·1)
Burundi	20049 (17136–23270)	20080 (17143–23307)	48·7 (32·3–71·8)	48·2 (31·5–69·7)
Cabo Verde	24627 (20839–28315)	24614 (20844–28315)	54·9 (36·7–79·5)	54·2 (35·9–77·4)
Cambodia	24927 (21524–29056)	24880 (21498–28901)	47·1 (31·4–67·2)	47·3 (31·5–67·8)
Cameroon	24617 (20870–28317)	24615 (20779–28242)	53·3 (35·4–76·1)	53·9 (36·1–76·2)
Canada	32937 (28606–37696)	32922 (28632–37680)	65·8 (45·6–94)	65·8 (44·9–94·5)
Central African Republic	23330 (19671–27005)	23329 (19692–26980)	51·4 (34·5–75·1)	51·6 (33·8–73·8)
Chad	24625 (20821–28302)	24616 (20824–28327)	53·4 (36·1–78·6)	53·4 (36·1–77·5)
Chile	25036 (21699–28724)	25892 (22115–30018)	57 (38·8–81·4)	58 (39·1–83·2)
China	17185 (15086–19366)	18564 (16311–20861)	39·6 (26·3–57·3)	41·4 (27·6–59·9)
Colombia	23770 (20635–27085)	23761 (20611–27109)	49·4 (33·1–69·7)	50·1 (33·8–70·8)
Comoros	20056 (17187–23306)	20068 (17158–23275)	48·8 (31·8–71·8)	48·9 (31·8–71·1)
Congo	23331 (19696–26984)	23338 (19729–27032)	52·2 (35·4–75·2)	52·3 (35–76·5)
Costa Rica	23760 (20575–27085)	23759 (20592–27156)	49·3 (33·2–68·8)	49·5 (33·6–69·9)
Côte d'Ivoire	24613 (20861–28250)	24603 (20838–28266)	52·2 (35·5–74·7)	52·8 (35·3–75·4)
Croatia	30272 (26376–34849)	30307 (26205–34565)	71·9 (47·5–102·9)	72 (48–103·2)

Cuba	23750 (20621–27069)	23755 (20601–27108)	49·6 (33–70·6)	49·8 (33–71·4)
Cyprus	32462 (28423–37498)	32472 (28459–37400)	69·5 (47–99·7)	70·2 (47·1–100·4)
Czechia	30268 (26393–34765)	30228 (26364–34686)	71·6 (48·5–102·2)	71·6 (47·9–101·5)
Democratic People's Republic of Korea	17754 (14949–21059)	17708 (14893–20983)	38·7 (25·6–55·1)	38·6 (24·8–55·7)
Democratic Republic of the Congo	23330 (19730–27085)	23336 (19707–27069)	51·7 (35–75·6)	51·9 (34·4–75·1)
Denmark	35031 (30698–39985)	34180 (29454–39398)	72 (48–101·4)	71·1 (47·9–100·9)
Djibouti	20087 (17166–23272)	20092 (17147–23278)	48·3 (31·8–70·5)	48·4 (31·7–71)
Dominica	23768 (20589–27133)	23745 (20584–27112)	49·8 (33–73)	49·5 (33·5–69·3)
Dominican Republic	23773 (20598–27123)	23773 (20594–27135)	49·5 (33·4–70·2)	49·7 (33·7–69·9)
Ecuador	17678 (15304–20592)	18916 (16081–22175)	43 (28·1–62·7)	44·8 (29·7–65·4)
Egypt	25277 (21683–29219)	26032 (22668–29727)	67·2 (44·7–97·4)	68·6 (45·8–99·5)
El Salvador	23775 (20620–27150)	23776 (20614–27113)	49·4 (33–69·2)	50·5 (34·2–71·5)
Equatorial Guinea	23315 (19659–26979)	23327 (19771–27079)	52·3 (34·9–77)	52·7 (34·7–76·9)
Eritrea	20038 (17178–23245)	20080 (17166–23285)	48·3 (31·9–71·7)	48·3 (30·9–69·9)
Estonia	31323 (27207–36059)	31338 (27159–36114)	86·3 (55·7–126)	85·8 (56·1–123·9)
Eswatini	23315 (19647–27037)	23332 (19687–27036)	53·3 (35·7–76·4)	52·4 (35·1–75·4)
Ethiopia	18394 (15986–21042)	17043 (14833–19366)	43·6 (28·7–64)	41·3 (27·7–60·4)
Fiji	22356 (19022–25805)	22359 (19054–25849)	44·7 (29·8–62·5)	44·6 (30·4–62·4)
Finland	32487 (28453–37380)	32456 (28422–37358)	69·8 (47–98·9)	69·5 (46·1–99·6)
France	31362 (27141–35936)	31360 (27194–35902)	74·3 (49·3–107·2)	74·5 (49·3–107·7)
Gabon	23343 (19720–27043)	23334 (19732–26951)	52·3 (35·3–75·9)	52·9 (35·1–76·9)
Gambia	24614 (20825–28261)	24620 (20818–28282)	53 (36–77·4)	53·7 (36·2–77·9)
Georgia	30872 (26679–35523)	30863 (26687–35438)	66·8 (45·2–94·3)	66·2 (44·9–93)
Germany	32629 (28460–37776)	32591 (28404–37706)	70 (47·3–100·1)	69·9 (46·7–101)
Ghana	24614 (20813–28310)	24619 (20834–28389)	53·6 (36–78·1)	54·3 (36·6–79·1)
Greece	32474 (28419–37460)	32468 (28473–37357)	69·7 (47·2–99·1)	69·7 (46·7–98·2)
Grenada	23780 (20608–27105)	23741 (20580–27057)	49·5 (33·4–69·3)	49·2 (33–69·4)
Guatemala	23772 (20604–27137)	23769 (20645–27117)	48·7 (32–68·9)	49·6 (33·5–68·8)

Guinea	24616 (20826–28245)	24618 (20816–28309)	53·4 (36·1–76·6)	54 (36·5–77·1)
Guinea-Bissau	24623 (20832–28321)	24619 (20837–28339)	53·4 (35·8–75·9)	53·9 (35·5–78)
Guyana	23771 (20630–27089)	23764 (20624–27108)	49·1 (33·1–69·8)	49·6 (33·2–69·6)
Haiti	23779 (20626–27085)	23768 (20615–27068)	48·9 (33·3–69·4)	49·3 (32·9–69·6)
Honduras	23766 (20560–27114)	23764 (20640–27052)	49·1 (32·1–69·2)	49·8 (34–71·5)
Hungary	30275 (26449–34674)	30250 (26352–34710)	71·5 (48·6–101·2)	71·9 (48·9–102·7)
Iceland	32463 (28446–37387)	32436 (28431–37354)	69·4 (47–99·7)	69 (47·1–98·5)
India	25818 (22921–28940)	25812 (22899–28918)	50·6 (34·3–71·8)	51·6 (34·9–73·1)
Indonesia	25990 (22959–29108)	25971 (22943–29094)	50·1 (34·4–71·2)	50·6 (34·8–71·1)
Iran (Islamic Republic of)	26920 (23892–30121)	27216 (23993–30596)	72·5 (48·4–105·4)	73·8 (49·4–107·8)
Iraq	23382 (19966–26804)	23386 (20064–26857)	64·7 (42·9–94·4)	65·3 (42·3–94·6)
Ireland	32471 (28431–37407)	32472 (28446–37401)	69·7 (47·6–99·9)	70·1 (47·1–100·2)
Israel	32482 (28431–37440)	32466 (28460–37398)	70·2 (47·5–101)	70 (47·2–99·9)
Italy	33376 (29852–37413)	33419 (29862–37077)	71·5 (49·2–101·2)	71·8 (49·1–101·7)
Jamaica	23774 (20611–27120)	23759 (20583–27169)	49·9 (33·6–71·1)	49·8 (33·2–71·4)
Japan	29843 (26324–33417)	29802 (26500–33333)	62·6 (42·5–89·3)	62·7 (42·9–89·5)
Jordan	23359 (20020–26728)	23389 (20028–26818)	65·3 (42–96·2)	65·3 (42·6–96·1)
Kazakhstan	30875 (26622–35441)	30868 (26670–35511)	66·2 (44·6–93·5)	66·4 (44·3–93·7)
Kenya	21206 (18426–24156)	21202 (18421–24158)	52·2 (34·6–75·6)	52·6 (34·9–76·1)
Kiribati	22398 (19058–25814)	22408 (19041–25866)	44·6 (29·6–62·7)	44·9 (29·7–63·1)
Kuwait	23415 (20065–27042)	23186 (19920–26613)	61·3 (40·7–90·9)	59·9 (39·5–86·5)
Kyrgyzstan	30873 (26628–35536)	30864 (26710–35459)	65·9 (43·9–93·3)	66·3 (44·3–93·7)
Lao People's Democratic Republic	24873 (21448–28950)	24839 (21402–28855)	46·9 (31·1–66·2)	47·3 (32–67)
Latvia	31322 (27171–36052)	31332 (27207–36092)	86·4 (57·2–123·8)	86·1 (56·4–123·2)
Lebanon	23349 (19994–26797)	23382 (20069–26802)	65·6 (43·2–97·3)	66 (43·1–97·7)
Lesotho	23301 (19619–26916)	23328 (19670–27048)	53·9 (36·1–79·2)	52·3 (35·4–75·2)
Liberia	24614 (20831–28206)	24618 (20840–28313)	52·3 (35·1–76·5)	52·9 (35·3–76)
Libya	23411 (20051–26797)	23380 (20050–26836)	64·7 (43·1–94·3)	65·5 (42·8–96·1)

Lithuania	32527 (28286–37356)	32548 (28389–37367)	79·4 (52·4–112·1)	79·3 (53·3–112·2)
Luxembourg	32146 (28002–36912)	32130 (27926–36965)	74·6 (50·3–108·7)	74·4 (50·2–106·9)
Madagascar	20066 (17139–23296)	20059 (17172–23244)	48·4 (31·7–70·3)	49 (32–71·7)
Malawi	20057 (17174–23237)	20051 (17150–23281)	48·5 (31·2–70·6)	49·1 (32·2–72·4)
Malaysia	24787 (21342–28719)	24805 (21382–28865)	47·4 (31·6–67·3)	47·4 (31·3–67)
Maldives	24789 (21360–28870)	24671 (21220–28726)	46·3 (31·1–65·3)	45·9 (31–64·6)
Mali	24615 (20844–28215)	24610 (20864–28321)	52·9 (35·6–76·8)	53 (34·8–76)
Malta	32477 (28443–37519)	32416 (28429–37391)	69·9 (47–101·2)	68·6 (46·6–98·3)
Marshall Islands	22340 (19030–25826)	22347 (19053–25834)	44·2 (29·8–62·3)	44·5 (29·1–62·8)
Mauritania	24620 (20910–28321)	24619 (20877–28343)	53·8 (35·7–79·3)	54·4 (36·6–78)
Mauritius	24852 (21473–28888)	24844 (21397–29008)	47·3 (31·2–66·6)	47·4 (32–67)
Mexico	24979 (21995–28221)	24974 (21987–28212)	52·4 (35·7–74·3)	52·9 (35·9–74·5)
Micronesia (Federated States of)	22349 (19036–25817)	22356 (19064–25831)	44·6 (29·5–63·6)	44·5 (29·3–63·5)
Monaco	32488 (28456–37400)	32475 (28446–37440)	70·5 (48·1–100·3)	70·4 (47·5–100·8)
Mongolia	30865 (26716–35552)	30867 (26681–35536)	65·6 (43·9–92·5)	66·6 (44·5–93·2)
Montenegro	30246 (26305–34670)	30225 (26333–34669)	71·8 (48·3–102·3)	71·8 (48·7–101·7)
Morocco	23358 (19987–26773)	23362 (20023–26760)	65·6 (43·3–97·2)	65·9 (43·8–97·1)
Mozambique	20039 (17153–23276)	20044 (17149–23248)	48·3 (31·8–71·5)	48·7 (31·8–71·3)
Myanmar	24865 (21445–28960)	24890 (21494–29006)	46·7 (31·1–66·1)	47·5 (31·7–67·5)
Namibia	23329 (19635–26973)	23330 (19654–26993)	52·9 (35·3–77)	53·1 (36·1–76·6)
Nauru	22348 (19023–25787)	22391 (19077–25812)	44·6 (29·5–62·8)	44·8 (30·1–62·8)
Nepal	25795 (22038–29731)	25522 (22225–29430)	50·3 (33·8–71·4)	51·5 (34·9–73·7)
Netherlands	35181 (30499–40144)	35170 (30504–40297)	69·1 (46·8–98)	68·7 (46·3–97·4)
New Zealand	27885 (24592–31107)	27853 (24546–30987)	61·2 (41·3–87·5)	61·3 (41·1–87·7)
Nicaragua	23775 (20613–27052)	23764 (20602–27132)	49·3 (32·8–69·5)	49·6 (33·1–70·1)
Niger	24623 (20837–28292)	24620 (20875–28328)	52·8 (35–75·5)	53·5 (35·9–76·8)
Nigeria	25751 (22871–28793)	25768 (22911–28819)	55·8 (37·3–80·3)	57·9 (39·1–83·3)
North Macedonia	30237 (26320–34738)	30229 (26358–34659)	71·5 (48·8–101·4)	71·8 (48·1–100·2)

Norway	35072 (31339–39281)	35151 (31487–38874)	69·7 (47·8–98·3)	71·4 (48·9–100·7)
Oman	23479 (20047–26897)	23473 (20007–26928)	62·5 (40·3–91·5)	63·5 (42·1–92)
Pakistan	27642 (24468–31059)	27581 (24365–30928)	49·6 (33·4–69·9)	46·2 (31·5–65·9)
Palau	22342 (19034–25784)	22287 (18990–25751)	44·8 (30–63·5)	44·3 (29·6–62·6)
Palestine	23367 (20021–26775)	23365 (20023–26805)	65·9 (42·5–96·7)	65·7 (43·1–96·9)
Panama	23755 (20597–27122)	23757 (20623–27173)	49·3 (33·2–69·2)	49·9 (32·9–71·3)
Papua New Guinea	22331 (19024–25790)	22328 (18999–25763)	44 (29·6–63·3)	44·1 (28·8–62·7)
Paraguay	26464 (22885–30411)	26459 (22910–30447)	49·9 (34·4–72)	50·2 (33·6–72·1)
Peru	22020 (19148–25434)	22092 (18922–25894)	47·6 (31·6–68·7)	48 (32·2–68·6)
Philippines	25983 (22956–29099)	25974 (22945–29094)	50·3 (34·5–71·2)	50·8 (34·7–71·7)
Poland	31700 (28059–35275)	31677 (28043–35258)	75·9 (51·3–108·8)	76·5 (51·4–110·2)
Portugal	32493 (28451–37496)	32483 (28435–37426)	69·9 (46·8–100·8)	70·1 (47·6–101)
Qatar	23439 (19953–26911)	23459 (19976–26994)	60·9 (39·4–89·4)	61·8 (40·4–89·7)
Republic of Korea	29115 (25304–33102)	29950 (25928–34673)	58·6 (39·6–82·6)	60·1 (40·8–85·9)
Republic of Moldova	31320 (27205–36036)	31331 (27230–35959)	86·4 (56·5–126·1)	86·1 (57·8–124·8)
Romania	30257 (26367–34691)	30241 (26339–34795)	71·5 (48–102·9)	72·1 (48·7–103·2)
Russian Federation	31846 (28283–35453)	32056 (28532–35860)	97·4 (63·6–141·4)	97·6 (64–141·1)
Rwanda	20051 (17141–23305)	20050 (17186–23276)	49 (32·5–72·3)	49·3 (32·5–71·2)
Saint Kitts and Nevis	23774 (20609–27182)	23773 (20600–27097)	49·7 (32·9–70·3)	50·3 (33·4–71·1)
Saint Lucia	23779 (20622–27132)	23756 (20621–27104)	49·8 (33·5–71)	49·5 (33·9–70)
Saint Vincent and the Grenadines	23772 (20601–27092)	23744 (20563–27109)	49·4 (32·8–70·7)	49·1 (32·7–68·9)
Samoa	22335 (19030–25757)	22362 (19010–25875)	44·5 (30·3–63·1)	44·8 (29·7–63·9)
San Marino	32477 (28429–37439)	32465 (28419–37386)	70 (47·3–101)	70 (47·8–99·3)
Sao Tome and Principe	24618 (20811–28346)	24618 (20814–28286)	54·1 (35·8–77·8)	54 (35·5–78·6)
Saudi Arabia	21976 (18829–25483)	22006 (18874–25502)	61·8 (40·2–89·3)	61·4 (39–89·2)
Senegal	24624 (20881–28318)	24605 (20801–28253)	53·3 (36–76·5)	53·7 (35·6–77·5)
Serbia	30247 (26313–34707)	30208 (26315–34592)	71·9 (47·8–102·1)	71·8 (47·9–101·7)
Seychelles	24844 (21448–28910)	24746 (21263–28845)	47·5 (31·2–67·6)	46·8 (31·4–67·3)

Sierra Leone	24614 (20844–28313)	24621 (20851–28318)	53 (35·7–76·6)	53·3 (35·5–76·9)
Singapore	26257 (24266–28325)	28539 (24548–32883)	54·5 (36·6–78·8)	57·2 (38·6–81·9)
Slovakia	30269 (26422–34677)	30241 (26379–34740)	71·8 (48·6–101·6)	72·1 (48·7–102·1)
Slovenia	30275 (26412–34787)	30194 (26314–34623)	71·8 (48·6–101·2)	71·3 (47·8–100·8)
Solomon Islands	22306 (18985–25722)	22343 (19055–25836)	44·2 (29–62·6)	44·4 (29·6–63·4)
Somalia	20072 (17183–23253)	20069 (17205–23319)	47·5 (31·1–69·5)	48·2 (31·3–69·6)
South Africa	24470 (21512–27421)	24472 (21519–27421)	56·5 (37·8–81·4)	56·3 (38–80·8)
South Sudan	20097 (17187–23300)	20050 (17178–23227)	47·3 (30·9–69·1)	48·2 (31·2–69)
Spain	31530 (27720–36201)	31530 (27682–36167)	72·4 (48·9–104·4)	73 (49–104·7)
Sri Lanka	24840 (21444–28863)	24876 (21435–28949)	47·2 (31·9–66·8)	47·9 (31·1–67·9)
Sudan	23344 (20035–26765)	23386 (20062–26819)	65·2 (41·3–95·1)	65·8 (42·7–96·1)
Suriname	23750 (20592–27108)	23762 (20598–27169)	49·4 (33·1–69·6)	49·7 (33·3–71·1)
Sweden	32421 (28393–37455)	32405 (28366–37296)	65 (44·3–93·7)	64·6 (43·5–90·3)
Switzerland	28307 (24423–32603)	29726 (25496–34221)	64·7 (43·2–93·3)	66·2 (44–95·1)
Syrian Arab Republic	23356 (20003–26760)	23343 (20081–26801)	64·9 (42–97)	65·7 (43·5–95·2)
Taiwan	17610 (14824–20848)	17672 (14877–21006)	38·4 (25·3–54·6)	39·1 (25·4–56·3)
Tajikistan	30867 (26692–35584)	30855 (26703–35521)	65·9 (44·1–92·4)	65·7 (43·8–93·3)
Thailand	24861 (21448–28931)	24873 (21409–28923)	47·2 (31·5–66·8)	47·9 (31·7–68)
Timor-Leste	24819 (21404–28919)	24825 (21424–28871)	46·2 (30·9–65·4)	47 (31·2–66·8)
Togo	24620 (20843–28300)	24620 (20843–28286)	53·9 (35·5–77·7)	54·2 (35·9–78·6)
Tonga	22381 (19046–25838)	22421 (19100–25912)	44·8 (28·9–63·5)	45·3 (29·6–63·8)
Trinidad and Tobago	23763 (20578–27057)	23762 (20595–27089)	49·4 (33·2–69·6)	49·6 (33·2–69·2)
Tunisia	23352 (19986–26770)	23360 (20038–26780)	65·6 (42–95·2)	66·5 (43·5–98·2)
Türkiye	21146 (18078–24691)	21140 (18120–24717)	65 (40·6–96·8)	65·7 (41·6–98·6)
Turkmenistan	30871 (26628–35537)	30860 (26716–35428)	66·2 (44·7–93·4)	65·8 (44·3–93·5)
Tuvalu	22445 (19096–25937)	22347 (19052–25787)	45·1 (29·9–63·6)	44·5 (29·5–62·9)
Uganda	20060 (17194–23287)	20045 (17187–23241)	48 (31·9–69·9)	49·2 (32·3–72·5)
Ukraine	32679 (29137–36847)	32685 (29179–36797)	90 (59–129·5)	90·2 (59·4–130·3)

United Arab Emirates	23511 (20044–26958)	23469 (20067–26923)	61·7 (40·6–89·7)	61·8 (40·8–87·8)
United Kingdom	33412 (29758–37847)	33422 (29731–37826)	72·2 (49·3–102·7)	72·2 (49·5–102)
United Republic of Tanzania	20051 (17163–23226)	20055 (17137–23266)	48·3 (32·7–70·6)	49 (31·9–71·7)
United States of America	35198 (31747–39161)	34104 (30349–37830)	69·9 (48·6–99·7)	68·5 (47·4–97·4)
Uruguay	25922 (22124–30054)	25925 (22156–29994)	58·2 (39·1–84·4)	58·4 (39·2–83)
Uzbekistan	30869 (26731–35409)	30858 (26662–35452)	65·9 (44·2–92·6)	66·4 (44·2–94·2)
Vanuatu	22321 (19015–25765)	22369 (19035–25811)	44 (29·9–62·7)	44·7 (29·6–63·7)
Venezuela (Bolivarian Republic of)	23767 (20606–27144)	23783 (20629–27133)	49·5 (32·5–70)	50 (33·2–72·1)
Viet Nam	24901 (21454–28940)	24852 (21449–28925)	47·6 (31·8–66·7)	47·7 (31·6–67·6)
Yemen	23373 (20029–26794)	23372 (20024–26792)	64·5 (42·3–95·7)	65·2 (42·7–96·5)
Zambia	21674 (18369–25309)	21671 (18318–25212)	54·7 (35·2–79·3)	56·5 (36·4–82·1)
Zimbabwe	23332 (19695–26969)	23320 (19633–26972)	52·6 (35·2–75·8)	53 (35·5–77)

8.2. Prevalence and burden of underlying headache diagnosis

Supplementary table 10 shows how migraine and TTH prevalence and years lived with disability was broken down into underlying headache diagnosis.

For migraine, MOH constituted 6·0% and 5·0% of the prevalence estimates in males and females respectively, but 22·6% and 14·1% of the YLD estimates. This was even more skewed in TTH where MOH constituted only 0·9% and 1·3% of the prevalence estimates in males and females respectively, but as high as 58·8% and 56·1% of the YLD estimates. Definite migraine covered 53·6% and 61·2% of prevalence in males and females respectively, and similar proportions of YLDs (54·4% and 65·5% respectively). Probable migraine, on the other hand, constituted 40·4% and 33·7% of the migraine prevalence estimates in males and females respectively, but only 23·0% and 20·5% of YLDs. Definite and probable TTH covered approximately half of the prevalence each (48·4% and 50·7% respectively in males, and 56·9% and 41·8 respectively in females), but much less of the YLDs (26·7% and 14·5% respectively in males, and 31·2% and 12·7% respectively in females).

Supplementary table 10. Global age-standardized prevalence and YLD estimates for migraine and tension-type headache by sex and underlying headache diagnosis (definite diagnosis, probable diagnosis, or medication overuse headache)

	**Migraine**				**Tension-type headache**			
	Prevalence		YLDs		Prevalence		YLDs	
	Rate*	%	Rate*	%	Rate*	%	Rate*	%
Males								
Definite	5 639	53·6	164·4	54·4	11 812	48·4	11·8	26·7
Probable	4 247	40·4	69·5	23·0	12 3734	50·7	6·4	14·5
MOH	628	6·0	68·2	22·6	230	0·9	25·9	58·8
Overall	10 514	100·0	302·1	100·0	24 416	100·0	44·1	100·0
Females								

Definite	10 800	61·2	442·0	65·5	14 495	56·9	20·2	31·2
Probable	5 948	33·7	138·3	20·5	10 633	41·8	8·2	12·7
MOH	889	5·0	94·9	14·1	326	1·3	36·3	56·1
Overall	17 637	100·0	675·2	100·0	25 453	100·0	64·7	100·0

*Age-standardized estimates per 100,000 persons

Supplementary figures 8 and 9 visualize the proportions reported in Table 10 as well as how these relate to age. In both males and females, migraine and TTH prevalences had a clear peak in the thirties. YLD rates, on the other hand, were as high in the forties-fifties as in the thirties.

Supplementary figure 8. Global prevalence of migraine and tension-type headache in males and females stratified by age, broken down by underlying headache diagnosis (definite diagnosis, probable diagnosis, or medication overuse headache

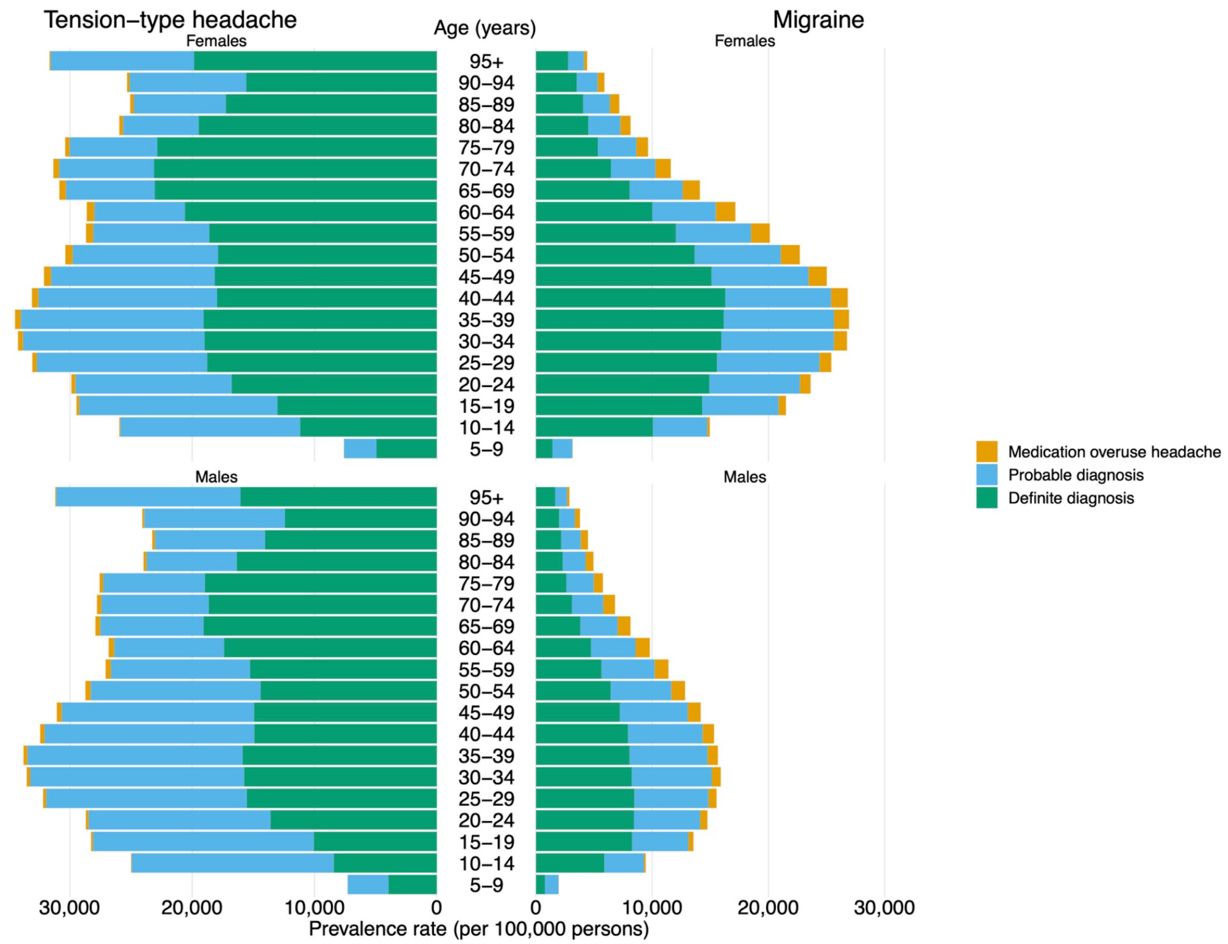

Supplementary figure 9. Global years lived with disability (YLD) rates (per 100,000 persons) of migraine and tension-type headache in males and females stratified by age, broken down by underlying headache diagnosis (definite diagnosis, probable diagnosis, or medication overuse headache)

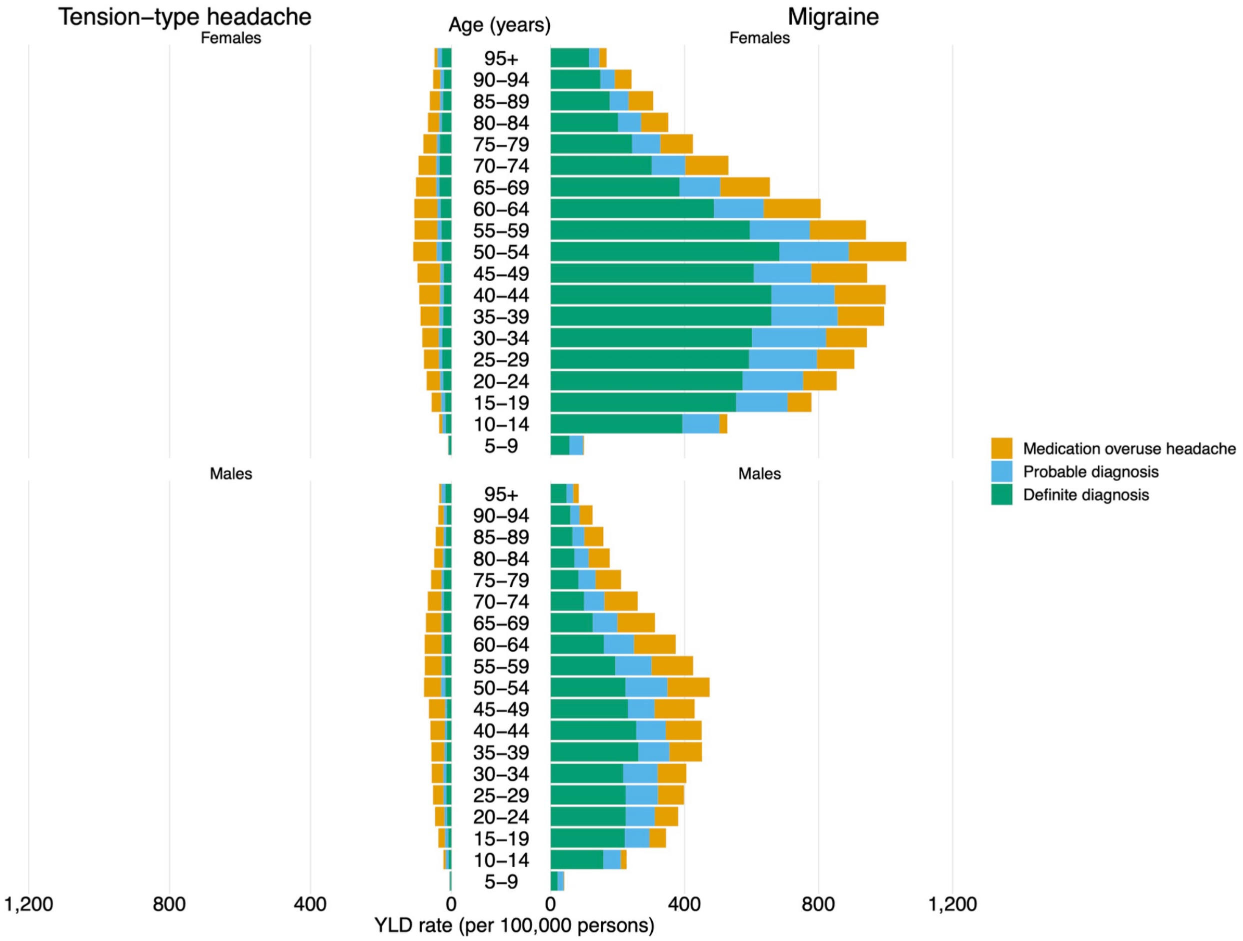

9. References

1. Headache Classification Committee of the International Headache Society (IHS) The International Classification of Headache Disorders, 3rd edition. *Cephalalgia* 2018; 38(1): 1-211.

2. Kim B-K, Chung YK, Kim J-M, Lee K-S, Chu MK. Prevalence, clinical characteristics and disability of migraine and probable migraine: A nationwide population-based survey in Korea. *Cephalalgia* 2013; **33**: 1106–16.

3. Lantéri-Minet M, Valade D, Géraud G, Chautard M, Lucas C. Migraine and Probable Migraine — Results of FRAMIG 3, a French Nationwide Survey Carried out According to the 2004 IHS Classification. *Cephalalgia* 2005; **25**: 1146–58.

4. Pfaffenrath V, Fendrich K, Vennemann M, *et al.* Regional Variations in the Prevalence of Migraine and Tension-Type Headache Applying the new IHS Criteria: The German DMKG Headache Study. *Cephalalgia* 2009; **29**: 48–57.

5. Rasmussen BK, Jensen R, Olesen J. A Population-Based Analysis of the Diagnostic Criteria of the International Headache Society. *Cephalalgia* 1991; **11**: 129–34.

6. Fendrich K, Vennemann M, Pfaffenrath V, *et al.* Headache Prevalence Among Adolescents — The German DMKG Headache Study. *Cephalalgia* 2007; **27**: 347–54.

7. Hay, S.I., Ong, L.K., […GBD DIRF Collaborators…]., Murray, C.L.J., Gakidou, E. Fatal and non-fatal burden of 375 diseases and injuries, disability adjusted life years, risk-attributable burden of 88 risk factors, and healthy life expectancy in 204 countries and territories, including 660 subnational locations, 1990-2023: a systematic analysis for the Global Burden of Disease Study 2023. *The Lancet*, submitted 18/03/25.

8. Steiner TJ, Stovner LJ, Al Jumah M, *et al.* Improving quality in population surveys of headache prevalence, burden and cost: key methodological considerations. *J Headache Pain* 2013; **14**: 87.
