# Supplementary material for: Global, regional, and national burden of headache disorders, 1990–2023: a systematic analysis for the Global Burden of Disease Study 2023
Source: Lancet Neurol. 2025 Dec;24(12):1005–15. doi: 10.1016/S1474-4422(25)00402-8 (PMC12612381; doi:10.1016/S1474-4422(25)00402-8)
Supplement: Supplementary appendix [file mmc2.pdf]

THE LANCET
Neurology

Supplementary appendix 2

This appendix formed part of the original submission and has been peer reviewed. We post it as supplied by the authors.

Supplement to: GBD 2023 Headache Collaborators. Global, regional, and national burden of headache disorders, 1990–2023: a systematic analysis for the Global Burden of Disease Study 2023. *Lancet Neurol* 2025; **24:** 1005–15.

Appendix 2: Authorship appendix to “Global, regional, and national burden of headache disorders, 1990-2023: a systematic analysis for the Global Burden of Disease Study 2023”

This appendix provides further authorship detail for “Global, regional, and national burden of headache disorders, 1990-2023: a systematic analysis for the Global Burden of Disease Study 2023”

Table of Contents

GBD 2023 Headache Collaborators **2**

Affiliations **3**

Authors’ Contributions **12**

- Managing the overall research enterprise 12
- Writing the first draft of the manuscript 12
- Primary responsibility for applying analytical methods to produce estimates 12
- Primary responsibility for seeking, cataloguing, extracting, or cleaning data; designing or coding figures and tables 13
- Providing data or critical feedback on data sources 13
- Developing methods or computational machinery 13
- Providing critical feedback on methods or results 14
- Drafting the work or revising it critically for important intellectual content 15
- Managing the estimation or publications process 16

GBD 2023 Headache Collaborators

Andreas Kattem Husøy, Yvonne Yiru Xu, Jaimie D Steinmetz, Mohammad Amin Aalipour, Hasan Aalruz, Deldar Morad Abdulah, Richard Gyan Aboagye, Dariush Abtahi, Samir Abu Rumeileh, Salahdein Aburuz, Qorinah Estiningtyas Sakilah Adnani, Obed Adonteng-Kissi, Giuseppina Affinito, Danish Ahmad, Negar Sadat Ahmadi, Ali Ahmed, Asma Ahmed, Shahzaib Ahmed, Mohammad Ahmmad Mahmoud Al Zoubi, Sawsan Alabbad, Yazan Al-Ajlouni, Mohammed Albashtawy, Fadwa Naji Alhalaiqa, Ashraf Alhumaidi, Mohammed Usman Ali, Syed Shujait Ali, Montaha Al-Iede, Joseph Uy Almazan, Najim Z Alshahrani, Awais Altaf, Mohammad Al-Wardat, Yaser Mohammed Al-Worafi, Karem H Alzoubi, Sohrab Amiri, Ganiyu Adeniyi Amusa, David B Anderson, Abhishek Anil, Jalal Arabloo, Aleksandr Y Aravkin, Demelash Areda, Mahsa Asadi Anar, Mohammad Asghari-Jafarabadi, Sait Ashina, Khursheed Aurangzeb, Arian Azadnia, Ahmed Y Azzam, Youngoh Bae, Razieh Bahreini, Soham Bandyopadhyay, Hiba Jawdat Barqawi, Azadeh Bashiri, Rehana Basri, Mohammad-Mahdi Bastan, Jina Behjati, Maryam Bemanalizadeh, Jeetendra Bhandari, Sonu Bhaskar, Gurjit Kaur Bhatti, Jasvinder Singh Bhatti, Rajbir Bhatti, Bijit Biswas, Bruno Bizzozero-Peroni, Archith Boloor, Meriem Boukhiam, Yasser Bustanji, Sanjay C J, Luis Alberto Cámera, Edoardo Caronna, Ana Paula Carvalho-e-Silva, Sandip Chakraborty, Vijay Kumar Chattu, Anis Ahmad Chaudhary, Patrick R Ching, Hongyuan Chu, Josielli Comachio, Daniela Contreras, Natalia Cruz-Martins, Omid Dadras, Xiaochen Dai, Emanuele D'Amico, Anh Kim Dang, Lucio D'Anna, Sindhura Deekonda, Pouria Delbari, Andreas K Demetriades, Emina Dervišević, Vinoth Gnana Chellaiyan Devanbu, Amol S Dhane, Bibha Dhungel, Xueting Ding, Huyen Phuc Do, Ojas Prakashbhai Doshi, Siddhartha Dutta, Lamiaa Labieb Mahmoud Ebraheim, Ebrahim Eini, Michael Ekholuenetale, Sharareh Eskandarieh, Andre Faro, Valery L Feigin, Gelana Fekadu, Ginenus Fekadu, Seyed-Mohammad Fereshtehnejad, Abdullah H Feroze, Pietro Ferrara, Nuno Ferreira, Claudio Fiorilla, Florian Fischer, Arianna Fornari, Celia Fortuna Rodrigues, Matteo Foschi, Abdelrahman Gamil Gad, Márió Gajdács, David Garcia-Azorin, Zisis Gatzioufas, Rupesh K Gautam, Miglas Welay Gebregergis, Elena V Gnedovskaya, Massimiliano Gobbo, Mahaveer Golechha, Pouya Goleij, Alessandra C Goulart, Ishita Gupta, Sapna Gupta, Roberth Steven Gutiérrez-Murillo, Najah R Hadi, Faraidoon Haghdoost, Hailey Hagins, Mohamed Hamed, Victoria Funmilayo Hanson, Amr Hassan, Simon I Hay, Khezar Hayat, Jeffrey J Hebert, Golnaz Heidari, Bartosz Helfer, Mehdi Hoseinzadeh, Md Jubayer Hossain, Yongsong Huang, Luigi Francesco Iannone, Segun Emmanuel Ibitoye, Olayinka Stephen Ilesanmi, Irena M Ilic, Muhana Fawwazy Ilyas, Salim Ilyasu, Md Rabiul Islam, Md Sahidul Islam, Nahlah Elkudssiah Ismail, Louis Jacob, Haitham Jahrami, Ammar Abdulrahman Jairoun, Navid Jamali, Manthan Dilipkumar Janodia, Ruwan Duminda Jayasinghe, Shuai Jin, Jost B Jonas, Nitin Joseph, Charity Ehimwenma Joshua, Saltanat Kamenova, Arun Kamireddy, Rami S Kantar, Sujita Kumar Kar, Mohmed Isaqali Karobari, Himanshu Khajuria, Sameer Uttamaro Khasbage, Jagdish Khubchandani, Yun Jin Kim, Omid Kohandel Gargari, Farzad Kompani, Aida Kondybayeva, Kewal Krishan, Barthelemy Kuate Defo, Mukhtar Kulimbet, Chandan Kumar, Rakesh Kumar, Vijay Kumar, Ville Kytö, Caterina Ledda, Seung Won Lee, Jacopo Lenzi, Jianan Li, Linyan Li, Yanxue Lian, Giancarlo Lucchetti, Jay B Lusk, Ricardo Lutzky Saute, Sasikumar Mahalingam, Rituparna Maiti, Ahmad Azam Malik, Birhanemaskal Malkamu, Vahid Mansouri, Konstantinos Margetis, Roy Rillera Marzo, Yasith Mathangasinghe, Georgios Mavrovounis, Hadush Negash Meles, Atte Meretoja, Tomislav Mestrovic, Sachith Mettananda, Bartosz Miazgowski, Giuseppe Minervini, Archana Mishra, Arup Kumar Misra, Khabab Abbasher Hussien Mohamed Ahmed, Omer Mohammed, Shafiu Mohammed, Ali H Mokdad, Shaher Momani, Maziar Moradi-Lakeh, Mahdis Morovvati, Reza Mosaddeghi Heris, Kavita Munjal, Yanjinlkham Munkhsaikhan, Efren Murillo-Zamora, Christopher J L Murray, Ghulam Mustafa, Fatemehzahra Naddafi, Zuhair S Natto, Gaurav Nepal,

Charles Richard James Newton, Cao Duy Nguyen, Cuong Tat Nguyen, Hien Thu Nguyen, Long Nguyen, Robina Khan Niazi, Luciano Nieddu, Fred Nugen, Chijindu N Nwakama, Ogochukwu Janet Nzoputam, Bogdan Oancea, Michael Safo Oduro, Hassan Okati-Aliabad, Andrew T Olagunju, Arão Belitardo Oliveira, Jia Ouyang, Mark Overton, Mayowa O Owolabi, Mahesh P A, Giuseppina Palena, Leonidas D Panos, Ioannis Pantazopoulos, Shahina Pardhan, Romil R Parikh, Arpit Parmar, Maja Pasovic, Shankargouda Patil, Apurba Patra, Paolo Pedersini, Mario F P Peres, Simone Perna, Wajida Perveen, Hai Quang Pham, Sanjay Prakash, Akila Prashant, Jagadeesh Puvvula, Maja R Radojčić, Alberto Raggi, Mohammad Meshbahur Rahman, Amir Masoud Rahmani, Mahmoud Mohammed Ramadan, Devarajan Rathish, Salman Rawaf, Mohsen Rezaeian, Taeho Gregory Rhee, Debby Syahru Romadlon, Michele Romoli, Marina Romozzi, Umar Saeed, Amene Saghazadeh, Amirhossein Sahebkar, Mohamed A Saleh, Sohrab Salimi, Abdallah M Samy, Lucas H C C Santos, Aswini Saravanan, Hemen Sarma, Yigit Can Senol, Yashendra Sethi, Yara Khaled Fouad Sayed Shaalan, Wajeehah Shahid, Anas Shamsi, Dan Shan, Amin Sharifan, Rekha Raghuveer Shenoy, Premalatha K Shetty, Zahra Shokati Eshkiki, Sunil Shrestha, Harmanjit Singh, Jasvinder A Singh, Satwinder Singh, Valentin Yurievich Skryabin, Farrukh Sobia, Reed J D Sorensen, Sebastian Straube, Chandan Kumar Swain, Sree Sudha T Y, Payam Tabaee Damavandi, Celine Tabche, Mohsan Tanveer, Minale Tareke, Claudia Baptista Tavares, Mohamad-Hani Temsah, Masayuki Teramoto, Arun James Thirunavukarasu, Ashutosh Tiwari, Thang Huu Tran, Nguyen Tran Minh Duc, Vasilis-Spyridon Tseriotis, Santhosh Kumar Tumkur Narayanappa, Aniefiok John Udoakang, Himayat Ullah, Jibrin Sammani Usman, Abdulkadir Usman Sambo, Jef Van den Eynde, Tommi Juhani Vasankari, Narayanaswamy Venketasubramanian, Jorge Hugo Villafañe, Arvinder Wander, Wei Wang, Xingxin Wang, Yuan-Pang Wang, Taweewat Wiangkham, Mieszko Wieckiewicz, Wanqing Xu, Saba Yahoo (Syed), Yazachew Engida Yismaw, Dong Keon Yon, Naohiro Yonemoto, Abdilahi Yousuf, Aurora Zanghì, Michael Zastrozhin, Anthony Lin Zhang, Zhongyi Zhao, Magdalena Zielińska, Kanyin Liane Ong, Timothy J Steiner, and Theo Vos.

Affiliations

Department of Neuromedicine and Movement Science (A K Husøy PhD, Prof T J Steiner PhD), NorHEAD Norwegian Center for Headache Research (D Contreras MD), Norwegian University of Science and Technology, Trondheim, Norway; Institute for Health Metrics and Evaluation (Y Xu MPH, J D Steinmetz PhD, A Y Aravkin PhD, X Dai PhD, Prof V L Feigin PhD, H Hagins MSPH, Prof S I Hay FMedSci, T Mestrovic PhD, Prof A H Mokdad PhD, Prof C J L Murray DPhil, M Pasovic MEd, R J D Sorensen PhD, K L Ong PhD, Prof T Vos PhD), Department of Applied Mathematics (A Y Aravkin PhD), Department of Health Metrics Sciences, School of Medicine (X Dai PhD, Prof S I Hay FMedSci, Prof A H Mokdad PhD, Prof C J L Murray DPhil, Prof T Vos PhD), Department of Global Health (R J D Sorensen PhD), University of Washington, Seattle, WA, USA; Department of Anesthesiology (Prof D Abtahi MD, S Salimi MD), School of Medicine (M Asadi Anar MD, J Behjati MD), Shahid Beheshti University of Medical Sciences, Tehran, Iran (M Aalipour MD); Department of Nursing (H Aalruz PhD), Al Zaytoonah University of Jordan, Amman, Jordan; Community and Maternity Nursing Unit (D M Abdulah MPH), University of Duhok, Duhok, Iraq; Department of Family and Community Health (R G Aboagye MPH), University of Health and Allied Sciences, Ho, Ghana; School of Population Health (R G Aboagye MPH), The George Institute for Global health (F Haghdoost PhD), University of New South Wales, Sydney, NSW, Australia; Department of Neurology (S Abu Rumeileh MD), Martin Luther University Halle-Wittenberg, Halle (Saale), Germany; Department of Pharmacology and Therapeutics (Prof S Aburuz PhD), United Arab Emirates University, Al Ain, United Arab Emirates; College of Pharmacy (Prof S Aburuz PhD), University of Jordan, Amman, Jordan; Department of Public Health (Q Adnani PhD), Universitas Padjadjaran (Padjadjaran University),

Bandung, Indonesia; School of Arts and Social Sciences (O Adonteng-Kissi PhD), Edith Cowan University, Bunbury, WA, Australia; Department of Public Health and Preventive Medicine (G Affinito PhD), Department of Public Health (C Fiorilla MD), University of Naples "Federico II", Naples, Italy; School of Medicine and Psychology (D Ahmad PhD), Australian National University, Canberra, ACT, Australia; Health Research Institute (D Ahmad PhD), University of Canberra, Canberra, NSW, Australia; School of Medicine (N S Ahmadi MD, M Morovvati MD), Non-communicable Diseases Research Center (M Bastan MD), Department of Pediatric Neurology (M Bemanalizadeh MD), Department of Neurosurgery (P Delbari MD), Multiple Sclerosis Research Center (S Eskandarieh PhD), Neurology Department (O Kohandel Gargari MD), Children's Medical Center (Prof F Kompani MD), Digestive Diseases Research Institute (V Mansouri MD), Research Center for Immunodeficiencies (A Saghazadeh MD), Tehran University of Medical Sciences, Tehran, Iran; Department of Pharmacy Practice (A Ahmed PhD), Riphah Institute of Pharmaceutical Sciences, Islamabad, Pakistan; Division of Infectious Diseases and Global Public Health (IDGPH) (A Ahmed PhD), University of California San Diego, San Diego, CA, USA; Institute of Molecular Biology and Biotechnology (A Ahmed PhD, A Altaf PhD), Department of Physics (W Shahid PhD), The University of Lahore, Lahore, Pakistan; Department of Medicine (S Ahmed MBBS), Fatima Memorial Hospital College of Medicine and Dentistry, Lahore, Pakistan; School of Public Health (M A M Al Zoubi PhD), University of Texas, Houston, TX, USA; School of Medicine (S Alabbad ABPN), Oral & Maxillofacial Rehabilitation Department (M Hamed DSc), Rabigh Faculty of Medicine (Prof A Malik PhD), Department of Dental Public Health (Prof Z S Natto DrPH), King Abdulaziz University, Jeddah, Saudi Arabia; Department of Neurology and Rehabilitation Medicine (S Alabbad ABPN), George Washington University, Arlington, VA, USA; Department of Rehabilitation (Y Al-Ajlouni MD), Montefiore Medical Center, Bronx, NY, USA; Department of Epidemiology (Y Al-Ajlouni MD), Columbia University, New York, NY, USA (D Shan PhD); Department of Community and Mental Health (Prof M Albashtawy PhD), Al al-Bayt University, Mafraq, Jordan; College of Nursing (Prof F N Alhalaiqa PhD), Department of Pharmaceutical Sciences (Prof K H Alzoubi PhD), Qatar University, Doha, Qatar; Department of Oral and Maxillofacial Surgery (A Alhumaidi DDS), Xi'an Jiaotong University, Xi'an, China; Department of Medical Rehabilitation (Physiotherapy) (M U Ali PhD), University of Maiduguri, Maiduguri, Nigeria; Nethersole School of Nursing (M U Ali PhD), The Chinese University of Hong Kong, Hong Kong, China; Center for Biotechnology and Microbiology (S S Ali PhD), University of Swat, Swat, Pakistan; The School of Medicine (M Al-Iede MD), School of Pharmacy (Prof Y Bustanji PhD), Department of Mathematics (Prof S Momani PhD), The University of Jordan, Amman, Jordan; Department of Medicine (J U Almazan PhD), Nazarbayev University, Astana, Kazakhstan; Department of Family and Community Medicine (N Z Alshahrani MD), University of Jeddah, Jeddah, Saudi Arabia; Faculty of Health Sciences (A Altaf PhD), Equator University of Science and Technology, Uganda, Masaka, Uganda; Department of Rehabilitation Sciences (M Al-Wardat PhD), Jordan University of Science and Technology, Irbid, Jordan; College Of Medical Sciences (Prof Y M Al-Worafi PhD), Fakeeh College for Medical Sciences, Jeddah, Saudi Arabia; College of Medical Sciences (Prof Y M Al-Worafi PhD), Azal University for Human Development, Sana'a, Yemen; Spiritual Health Research Center (S Amiri PhD), Baqiyatallah University of Medical Sciences, Tehran, Iran; Department of Medicine (G A Amusa MD), University of Jos, Jos, Nigeria; Department of Internal Medicine (G A Amusa MD), Jos University Teaching Hospital, Jos, Nigeria; Faculty of Medicine and Health (D B Anderson PhD, J Comachio PhD), Sydney Musculoskeletal Health (D B Anderson PhD), School of Health Science (A Carvalho-e-Silva PhD), University of Sydney, Sydney, NSW, Australia; Department of Pharmacology (A Anil MD, Prof R Maiti MD, A Mishra DM), Department of Psychiatry (A Parmar DM), All India Institute of Medical Sciences, Bhubaneswar, India; Health Management and Economics Research

Center (J Arabloo PhD), School of Medicine (M Bastan MD), Gastrointestinal and Liver Diseases Research Center (Prof M Moradi-Lakeh MD), Preventive Medicine and Public Health Research Center (Prof M Moradi-Lakeh MD), Iran University of Medical Sciences, Tehran, Iran; Department of Health Metrics Sciences, School of Medicine (A Y Aravkin PhD), University of Washington, Seattle, WA, United States of America; College of Art and Science (D Areda PhD), Ottawa University, Surprise, AZ, USA; School of Life Sciences (D Areda PhD), Arizona State University, Tempe, AZ, USA; College of Medicine (M Asadi Anar MD), University of Arizona, Tucson, AZ, USA; Cabrini Research (Prof M Asghari-Jafarabadi PhD), Cabrini Health, Malvern, VIC, Australia; School of Public Health and Preventive Medicine (Prof M Asghari-Jafarabadi PhD), Monash University, Melbourne, VIC, Australia; Department of Anesthesia, Critical Care and Pain Medicine (S Ashina MD), Department of Social and Behavioral Sciences (W Xu MPH), Harvard University, Boston, MA, USA; Department of Clinical Medicine (S Ashina MD), Department of Neurology (Prof T J Steiner PhD), University of Copenhagen, Copenhagen, Denmark; Department of Computer Engineering (K Aurangzeb PhD), Pediatric Intensive Care Unit (Prof M Temsah MD), King Saud University, Riyadh, Saudi Arabia; Research and Technology Deputy (A Azadnia PhD), Kurdistan University of Medical Sciences, Sanandaj, Iran; ASIDE Healthcare, Lewes, DE, USA (A Y Azzam MD); Faculty of Medicine (A Y Azzam MD), October 6 University, 6th of October City, Egypt; Department of Precision Medicine (Y Bae MD), Sungkyunkwan University, Seongnam, South Korea; College of Optometry (R Bahreini MS), Pacific University, Forest Grove, OR, USA; Nuffield Department of Surgical Sciences (S Bandyopadhyay MPH), Department of Psychiatry (Prof C R J Newton MD), Centre for Global Epilepsy (M Romoli MD), University of Oxford, Oxford, UK; Department of Neurosurgery (S Bandyopadhyay MPH), University of Southampton, Southampton, UK; Clinical Sciences Department (H J Barqawi MPhil), Department of Basic Biomedical Sciences (Prof Y Bustanji PhD), Department of Clinical Sciences (Prof M M Ramadan PhD), College of Medicine (Prof M A Saleh PhD), University of Sharjah, Sharjah, United Arab Emirates; Health Information Management (A Bashiri PhD), Shiraz University of Medical Sciences, Shiraz, Iran; College of Medicine (R Basri PhD), Jouf University, Sakaka, Saudi Arabia; Department of Pediatrics (M Bemanalizadeh MD), Isfahan University of Medical Sciences, Isfahan, Iran; Department of General Practice and Emergency Medicine (J Bhandari MD), Karnali Academy of Health Sciences, Jumla, Nepal; Global Health Neurology Lab (S Bhaskar MD), NSW Brain Clot Bank, Sydney, NSW, Australia; Division of Cerebrovascular Medicine and Neurology (S Bhaskar MD), National Cerebral and Cardiovascular Center, Suita, Japan; Department of Medical Lab Technology (Prof G K Bhatti PhD), Chandigarh University, Mohali, India; Laboratory of Translational Medicine and Nanotherapeutics (Prof J S Bhatti PhD), Department of Computer Science & Engineering (Prof S Singh PhD), Central University of Punjab, Bathinda, India; Department of Pharmaceutical Sciences (Prof R Bhatti PhD), Guru Nanak Dev University, Amritsar, India; Department of Community and Family Medicine (B Biswas MD), Department of Pharmacology (S T Y MD), All India Institute of Medical Sciences, Deoghar, India; Department of Neurobiology, Care Sciences and Society (B Bizzozero-Peroni PhD), Department of Neurobiology, Care Sciences, and Society (S Fereshtehnejad PhD), Karolinska Institute, Stockholm, Sweden; Department of Physical Education and Health (B Bizzozero-Peroni PhD), Universidad de la República, Rivera, Uruguay; Department of Internal Medicine (A Boloor MD), Department of Community Medicine (N Joseph MD), Manipal Academy of Higher Education, Mangalore, India; Faculty of Medicine (M Boukhiam MD), Mohammed VI University of Health Science, Casablanca, WA, Morocco ; JSS Dental College & Hospital (S C J MDS), Department of Respiratory Medicine (Prof M P A DNB), Jagadguru Sri Shivarathreeswara University, Mysore, India; Department of Internal and Geriatric Medicine (Prof L A Cámera MD), Hospital Italiano de Buenos Aires (Italian Hospital of Buenos Aires), Buenos Aires, Argentina; Board of Directors

(Prof L A Cámera MD), Argentine Society of Medicine, Buenos Aires, Argentina; Headache and Neurological Pain Research Group (E Caronna PhD), Vall d´Hebron Research Institute (VHIR), Barcelona, Spain; Education Center of Australia (A Carvalho-e-Silva PhD), Health Science College, Sydney, NSW, Australia; State Disease Investigation Laboratory (S Chakraborty MVSc), Animal Resources Development Department, Agartala, India; Department of Epidemiology and Biostatistics (V Chattu PhD), Semey Medical University (SMU), Semey, Kazakhstan; Department of Community Medicine (V Chattu PhD), Datta Meghe Institute of Medical Sciences, Sawangi, India; Department of Biology (A A Chaudhary PhD), Imam Mohammad Ibn Saud Islamic University, Riyadh, Saudi Arabia; Division of Infectious Diseases (P R Ching MD), Virginia Commonwealth University, Richmond, VA, USA; Department of Pediatrics (H Chu PhD), Peking University People's Hospital (J Ouyang PhD), Peking University, Beijing, China; Department of Clinical Medicine (D Contreras MD), University of Bergen, Bergen, Norway; Life and Health Sciences Research Institute (ICVS) (Prof N Cruz-Martins PhD), University of Minho, Braga, Portugal; Institute for Research and Innovation in Health (i3S) (Prof N Cruz-Martins PhD), Applied Molecular Biosciences Unit (Prof C Fortuna Rodrigues PhD), Faculty of Engineering (Prof C Fortuna Rodrigues PhD), University of Porto, Porto, Portugal; Department of Health (O Dadras PhD), Northern Territory Government, Darwin, WA, Australia; Department of Medical and Surgical Sciences and Advanced Technologies "GF Ingrassia" (Prof E D'Amico MD), Department of Clinical and Experimental Medicine (Prof C Ledda PhD), University of Catania, Catania, Italy; Institute for Global Health Innovations (A K Dang MD, C T Nguyen MPH), Duy Tan University, Hanoi, Vietnam; Department of Brain Sciences (L D'Anna PhD), Department of Primary Care and Public Health (Prof S Rawaf MD, C Tabche MSc), Imperial College London, London, UK; Department of Pediatrics (S Deekonda MD), Brookdale University Hospital Medical Center, Brooklyn, NY, USA; Department of Neurosurgery (A K Demetriades MD), University of Edinburgh, Edinburgh, UK; Department of Neurosurgery (A K Demetriades MD), National Health Service (NHS) Scotland, Edinburgh, UK; Department of Forensic Medicine (E Dervišević PhD), University of Sarajevo, Sarajevo, Bosnia and Herzegovina; Chettinad Hospital & Research Institute (Prof V Devanbu MD), Chettinad Academy of Research and Education, Chennai, India; Research and Development Cell (A S Dhane MBA), Dr. D. Y. Patil Vidyapeeth, Pune (Deemed to be University), Pune, India; Population Interventions Unit (B Dhungel DrPH), School of Health Sciences (A Meretoja MD), University of Melbourne, Melbourne, VIC, Australia; Joe C. Wen School of Population & Public Health (X Ding MA), University of California Irvine, Irvine, CA, USA; College of Health Sciences (H P Do PhD), VinUniversity, Hanoi, Vietnam; Institute of Health Economics and Technology (iHEAT), Hanoi, Vietnam (H P Do PhD); Independent Consultant, Bridgewater, NJ, USA (O P Doshi MS); Department of Pharmacology (S Dutta MD), All India Institute of Medical Sciences, Rajkot, India; Histology Department (L L M Ebraheim PhD), Zagazig University, Zagazig, Egypt; Independent Consultant, Ahvaz, Iran (E Eini MSD); Faculty of Science and Health (M Ekholuenetale PhD), University of Portsmouth, Hampshire, UK; Department of Psychology (A Faro PhD), Federal University of Sergipe, São Cristóvão, Brazil; National Institute for Stroke and Applied Neurosciences (Prof V L Feigin PhD), Auckland University of Technology, Auckland, New Zealand; Third Department of Neurology (E V Gnedovskaya PhD), Research Center of Neurology, Moscow, Russia (Prof V L Feigin PhD); School of Nursing (G Fekadu MSc), Haramaya University, Harar, Ethiopia; Department of Infectious Diseases and Public Health (G Fekadu PhD, L Li DSc), City University of Hong Kong, Hong Kong, China; Department of Pharmacy (G Fekadu PhD), Wollega University, Nekemte, Ethiopia; Division of Neurology (S Fereshtehnejad PhD), University of Toronto, Toronto, ON, Canada; Department of Neurosurgery (A H Feroze MD), Children's National Medical Center, Washington, DC, USA; Department of Neurosurgery (A H Feroze MD), George Washington University, Washington, DC, USA; Center for Public Health Research

(P Ferrara PhD), University of Milan Bicocca, Monza, Italy; Laboratory of Public Health (P Ferrara PhD), IRCCS Istituto Auxologico Italiano, Milan, Italy; Department of Social Sciences (Prof N Ferreira PhD), University of Nicosia, Nicosia, Cyprus; Institute of Public Health (F Fischer PhD), Charité Universitätsmedizin Berlin (Charité Medical University Berlin), Berlin, Germany; UO Neurologia, Salute Pubblica e Disabilità (The Neurology, Public Health and Disability Unit) (A Fornari PhD, A Raggi PhD), Fondazione IRCCS Istituto Neurologico Carlo Besta (IRCCS Foundation Carlo Besta Neurological Institute), Milan, Italy; Department of Neuroscience (M Foschi MD), Multiple Sclerosis Research Center, Ravenna, Italy; Department of Biotechnological and Applied Clinical Sciences (M Foschi MD), University of L'Aquila, L'Aquila, Italy; Russell H. Morgan Department of Radiology and Radiological Science (A G Gad MD, A Kamireddy MD), School of Medicine (C N Nwakama MD), Johns Hopkins University, Baltimore, MD, USA; Department of Public Health (M Gajdács PhD), University of Szeged, Szeged, Hungary; Department of Medicine (Prof D Garcia-Azorin MD), University of Valladolid, Valladolid, Spain; Department of Neurology (Prof D Garcia-Azorin MD), Hospital Universitario Rio Hortega, Valladolid, Spain; Department of Ophthalmology (Prof Z Gatzioufas PhD), University of Basel, Basel, Switzerland; Centre for Pharmacology (Prof R K Gautam PhD), Amity Institute of Pharmacy, Noida, India; Department of Midwifery (M W Gebregergis MSc), Department of Medical Laboratory Sciences (H N Meles MSc), Adigrat University, Adigrat, Ethiopia; Department of Clinical and Experimental Sciences (Prof M Gobbo MD, P Pedersini MSc), University of Brescia, Brescia, Italy; IRCSS Fondazione Don Gnocchi (Prof M Gobbo MD), IRCCS Fondazione Don Gnocchi, Rovato (Brescia), Italy; Department of Health Systems and Policy Research (Prof M Golechha PhD), Indian Institute of Public Health, Gandhinagar, India; Department of Genetics (P Goleij MSc), Sana Institute of Higher Education, Sari, Iran; Universal Scientific Education and Research Network (USERN) (P Goleij MSc), Kermanshah University of Medical Sciences, Kermanshah, Iran; Department of Epidemiology (Prof A C Goulart PhD), Universidade de São Paulo (University of São Paulo), São Paulo, Brazil; Department of Internal Medicine (I Gupta MD), Independent Consultant, Bharatpur, India; Independent Consultant, Delhi, India (I Gupta MD); Department of Toxicology (S Gupta PhD), Shriram Institute for Industrial Research, Delhi, India; Doctoral Program in Biomedical Gerontology (R S Gutiérrez-Murillo PhD), Pontifical Catholic University of Rio Grande do Sul, Porto Alegre, Brazil; Department of Clinical Pharmacology and Medicine (Prof N R Hadi PhD), University of Kufa, Najaf, Iraq; Department of Fixed Prosthodontics (M Hamed DSc), Department of Neurology (Prof A Hassan MD), Cairo University, Cairo, Egypt; Rak Medical and Health Sciences University (V F Hanson PhD), Rak Medical and Health Sciences University, Ras Al-Khaimah, United Arab Emirates; Institute of Pharmaceutical Sciences (K Hayat MS), University of Veterinary and Animal Sciences, Lahore, Pakistan; Department of Pharmacy Administration and Clinical Pharmacy (K Hayat MS), Xian Jiaotong University, Xian, China; Faculty of Kinesiology (Prof J J Hebert PhD), University of New Brunswick, Fredericton, NB, Canada; School of Allied Health (Prof J J Hebert PhD), Murdoch University, Murdoch, WA, Australia; Independent Consultant, Santa Clara, CA, USA (G Heidari MD); Institute of Psychology (B Helfer PhD), University of Wroclaw, Wroclaw, Poland; Meta Research Centre (B Helfer PhD), University of Wrocław, Wroclaw, Poland; School of Computer Science (Prof M Hoseinzadeh PhD), Institute for Global Health Innovations (C Nguyen MD, H T Nguyen MD), Duy Tan University, Da Nang, Vietnam; Department of AI (Prof M Hoseinzadeh PhD), Galgotias University, Greater Noida, India; Center for Health Innovation, Research, Action and Learning - Bangladesh (CHIRAL Bangladesh), Dhaka, Bangladesh (M Hossain MSc); Department of Public Health (M Hossain MSc), Daffodil International University, Dhaka, Bangladesh; Advanced Institute of Convergence Knowledge Informatics (Y Huang PhD), Graduate School of Engineering (Y Huang PhD), Tohoku University, Sendai, Japan; Department of Biomedical, Metabolic, and

Neural Science (L F Iannone MD), University of Modena and Reggio Emilia, Modena, Italy; Department of Health Promotion and Education (S Ibitoye PhD), Department of Medicine (Prof M O Owolabi DrM), University of Ibadan, Ibadan, Nigeria; West Africa RCC (O S Ilesanmi PhD), Africa Centre for Disease Control and Prevention, Abuja, Nigeria; Department of Community Medicine (O S Ilesanmi PhD), Department of Medicine (Prof M O Owolabi DrM), University College Hospital, Ibadan, Ibadan, Nigeria; Faculty of Medicine (I M Ilic PhD), University of Belgrade, Belgrade, Serbia; Department of Neurosurgery (M F Ilyas MD), Universitas Sebelas Maret (March Eleventh University), Jakarta, Indonesia; Department of Pharmaceutics and Pharmaceutical Technology (S Ilyasu PhD), Department of Physiotherapy (J S Usman PhD), Bayero University, Kano, Nigeria; School of Pharmacy (M Islam PhD), BRAC University, Dhaka, Bangladesh; Research and Publication Department (M Islam MSc), World Health Organization (WHO), Dhaka, Bangladesh; Department of Clinical Pharmacy & Pharmacy Practice (Prof N E Ismail PhD), Asian Institute of Medicine, Science and Technology, Bedong, Malaysia; Malaysian Academy of Pharmacy, Puchong, Malaysia (Prof N E Ismail PhD); Department of Physical Medicine and Rehabilitation (L Jacob MD), Université Paris Cité, Paris, France; Research and Development Unit (L Jacob MD), Biomedical Research Networking Center for Mental Health Network (CiberSAM), Barcelona, Spain; College of Medicine and Health Sciences (H Jahrami PhD), Arabian Gulf University, Manama, Bahrain; Government Hospitals, Manama, Bahrain (H Jahrami PhD); Department of Health and Safety (A A Jairoun PhD), Dubai Municipality, Dubai, United Arab Emirates; Department of Laboratory Sciences (N Jamali PhD), Sirjan School of Medical Sciences, Sirjan, Iran; Malla Reddy Vishwavidyapeeth, Hyderabad, India (Prof M D Janodia PhD); Sri Devraj Urs Academy of Higher Education and Research, Kolar, India (Prof M D Janodia PhD); Department of Oral Medicine and Periodontology (Prof R D Jayasinghe MS), University of Peradeniya, Peradeniya, Sri Lanka; Department of Oral Medicine and Periodontology (Prof R D Jayasinghe MS), Saveetha Medical College and Hospital (Prof M Karobari PhD), Saveetha Dental College and Hospitals (G Minervini PhD), Center for Global Health Research (Prof A Sahebkar PhD), Saveetha University, Chennai, India; School of Biology and Engineering (S Jin MPH), Guizhou Medical University, Guiyang, China; Rothschild Foundation Hospital (Prof J B Jonas MD), Institut Français de Myopie, Paris, France; Singapore Eye Research Institute (Prof J B Jonas MD), Singapore Eye Research Institute, Singapore, Singapore; Department of Economics (C E Joshua BSc), National Open University, Benin City, Nigeria; Department of General Medical Practice No. 2 (Prof S Kamenova DMedSc), Scientific and Educational Center for Neurology and Applied Neuroscience (A Kondybayeva PhD), Research and Publication Activity Division (M Kulimbet MSc), Kazakh National Medical University, Almaty, Kazakhstan; The Hansjörg Wyss Department of Plastic and Reconstructive Surgery (R S Kantar MD), NYU Langone Health, New York, NY, USA; Cleft Lip and Palate Surgery Division (R S Kantar MD), Global Smile Foundation, Norwood, MA, USA; Department of Psychiatry (S K Kar MD), King George's Medical University, Lucknow, India; Amity Institute of Forensic Sciences (H Khajuria PhD), Amity Institute of Pharmacy (K Munjal PhD), Amity University, Noida, India; Department of Pharmacology (S U Khasbage MD), All India Institute of Medical Sciences, Raipur, India; Department of Public Health (J Khubchandani PhD), New Mexico State University, Las Cruces, NM, USA; School of Traditional Chinese Medicine (Y Kim PhD), Xiamen University Malaysia, Sepang, Malaysia; Department of Medicine (O Kohandel Gargari MD), Alborz University of Medical Sciences, Karaj, Iran; Department of Anthropology (Prof K Krishan PhD), Panjab University, Chandigarh, India; Department of Demography (Prof B Kuate Defo PhD), Department of Social and Preventive Medicine (Prof B Kuate Defo PhD), University of Montreal, Montreal, QC, Canada; Center of Medicine and Public Health (M Kulimbet MSc), Asfendiyarov Kazakh National Medical University, Almaty, Kazakhstan; Amity Institute of Health Allied Sciences (Prof C Kumar PhD), Amity

University Uttar Pradesh, Noida, India; Department of Health Management (R Kumar PhD), University of Hail, Hail, Saudi Arabia; Department of Economics (V Kumar PhD), Manipal University, Jaipur, India; Clinical Research Center (V Kytö MD), Turku University Hospital, Turku, Finland; Heart Center (V Kytö MD), University of Turku and Turku University Hospital, Turku, Finland; Department of Precision Medicine (Prof S Lee MD), Sungkyunkwan University, Suwon-si, South Korea; Department of Biomedical and Neuromotor Sciences (J Lenzi PhD), University of Bologna, Bologna, Italy; School of Public Health (Prof J Li PhD), Xuzhou medical university, Xuzhou, China; Discipline of Physiology (Y Lian MA), National University of Ireland - Galway, Galway, Ireland; School of Medicine (Prof G Lucchetti PhD), Federal University of Juiz de Fora, Juiz de Fora, Brazil; Department of Population Health Sciences (J B Lusk MD), Duke University, Durham, NC, USA; Department of Family Medicine (J B Lusk MD), University of North Carolina Chapel Hill, Chapel Hill, NC, USA; Department of Neurosciences and Behavioral Sciences (R Lutzky Saute MD), University of São Paulo, Ribeirão Preto, Brazil; Department of Emergency Medicine (S Mahalingam MD), Sri Lakshmi Narayana Institute of Medical Science, Puducherry, Pondicherry, India; College of Health Sciences (B Malkamu MSc), Debre Tabor University, Debre Tabor, Ethiopia; Department of Neurosurgery (K Margetis MD), Icahn School of Medicine at Mount Sinai, New York, NY, USA; Faculty of Humanities and Health Sciences (Prof R R Marzo MD), Curtin University, Sarawak, Malaysia; Jeffrey Cheah School of Medicine and Health Sciences (Prof R R Marzo MD), Monash University, Subang Jaya, Malaysia; Department of Anatomy and Developmental Biology (Y Mathangasinghe PhD), Monash University, Clayton, VIC, Australia; Department of Anatomy, Genetics and Biomedical Informatics (Y Mathangasinghe PhD), University of Colombo, Colombo, Sri Lanka; Department of Emergency Medicine (G Mavrovounis MSc, Prof I Pantazopoulos PhD), University of Thessaly, Larissa, Greece; General Administration Department (A Meretoja MD), Helsinki University Hospital, Helsinki, Finland; University Centre Varazdin (T Mestrovic PhD), University North, Varazdin, Croatia; Department of Paediatrics (Prof S Mettananda DPhil), University of Kelaniya, Ragama, Sri Lanka; University Paediatrics Unit (Prof S Mettananda DPhil), Colombo North Teaching Hospital, Ragama, Sri Lanka; Clinical Emergency Department (B Miazgowski MD), Pomeranian Medical University, Szczecin, Poland (B Miazgowski MD); Multidisciplinary Department of Medical-Surgical and Dental Specialties (G Minervini PhD), University of Campania Luigi Vanvitelli, Naples, Italy; Department of Pharmacology (A K Misra MD), All India Institute of Medical Sciences, Mangalagiri, India; Faculty of Medicine (K Mohamed Ahmed MD), University of Khartoum, Khartoum, Sudan; Department of Medicine (O Mohammed MBBS), Government Medical College Kozhikode, Kozhikode, India; Health Systems and Policy Research Unit (Prof S Mohammed PhD), Ahmadu Bello University, Zaria, Nigeria; Heidelberg Institute of Global Health (HIGH) (Prof S Mohammed PhD), Heidelberg University, Heidelberg, Germany; Nonlinear Dynamics Research Center (NDRC) (Prof S Momani PhD), Center for Medical and Bio-Allied Health Sciences Research (A Shamsi PhD), Ajman University, Ajman, United Arab Emirates; Baan Clinic, Tehran, Iran (M Morovvati MD); Neurosciences Research Center (NSRC) (R Mosaddeghi Heris MD), Student Research Committee (R Mosaddeghi Heris MD), Department of Geriatric Health (F Naddafi PhD), Tabriz University of Medical Sciences, Tabriz, Iran; Department of Community and Global Health (Y Munkhsaikhan MD), The University of Tokyo, Tokyo, Japan; Clinical Epidemiology Research Unit (E Murillo-Zamora PhD), Mexican Institute of Social Security, Villa de Alvarez, Mexico; Postgraduate in Medical Sciences (E Murillo-Zamora PhD), Universidad de Colima, Colima, Mexico; College of Medicine (Prof G Mustafa MD, H Ullah FCPS), Shaqra University, Shaqra, Saudi Arabia; Department of Pediatrics & Pediatric Pulmonology (Prof G Mustafa MD), Institute of Mother & Child Care, Multan, Pakistan; Department of Health Education & Promotion (F Naddafi PhD), Gonabad University of Medical Sciences, Gonabad, Iran;

Department of Neurology (G Nepal MD), Case Western Reserve University, Cleveland, OH, USA; Department of Neurosciences (Prof C R J Newton MD), Kenya Medical Research Institute/Wellcome Trust Research Programme, Kilifi, Kenya; Faculty of Public Health (L Nguyen PhD), International Institute for Training and Research (INSTAR) (L Nguyen PhD), VNU University of Medicine and Pharmacy, Hanoi, Vietnam; International Islamic University Islamabad, Islamabad, Pakistan (R K Niazi PhD); Department of Humanities and Social Science (L Nieddu PhD), University for International Studies in Rome, Rome, Italy; Department of Radiology (F Nugen PhD), Mayo Clinic, Rochester, MN, USA; School of Information (F Nugen PhD), University of California Berkeley, Berkeley, CA, USA; Department of Physiology (O J Nzoputam PhD), University of Benin, Edo, Nigeria; Department of Physiology (O J Nzoputam PhD), Benson Idahosa University, Benin City, Nigeria; Department of Applied Economics and Quantitative Analysis (Prof B Oancea PhD), University of Bucharest, Bucharest, Romania; Bioinformatics Department (Prof B Oancea PhD), National Institute of Research and Development for Biological Sciences, Bucharest, Romania; Pfizer Research & Development (M Oduro PhD), Pfizer Inc., Groton, CT, USA; Health Promotion Research Center (H Okati-Aliabad PhD), Zahedan University of Medical Sciences, Zahedan, Iran; Department of Psychiatry and Behavioural Neurosciences (Prof A T Olagunju PhD), McMaster University, Hamilton, ON, Canada; Department of Psychiatry (Prof A T Olagunju PhD), University of Lagos, Lagos, Nigeria; Center for Clinical and Epidemiological Research (A B Oliveira PhD), Department of Psychiatry (Prof M F P Peres MD, Y Wang PhD), University of São Paulo, São Paulo, Brazil; Associação Brasileira de Cefaleia em Salvas e Enxaqueca (ABRACES), São Paulo, Brazil (A B Oliveira PhD); Graduate School of Health (M Overton PhD), University of Technology Sydney, Sydney, NSW, Australia; Department of Public Health (G Palena MD), University “Federico II” of Naples, Naples, Italy; Department of Neurology (L D Panos MD), University of Bern, Biel/Biene, Switzerland; Department of Neurology (L D Panos MD), University of Cyprus, Nicosia, Cyprus; Department of Emergency Medicine (Prof I Pantazopoulos PhD), University of Bern, Bern, Switzerland; Vision and Eye Research Institute (Prof S Pardhan PhD), Anglia Ruskin University, Cambridge, UK; Division of Health Policy and Management (R R Parikh MD), University of Minnesota, Minneapolis, MN, USA; College of Dental Medicine (Prof S Patil PhD), Roseman University of Health Sciences, South Jordan, UT, USA; Department of Human Anatomy (A Patra MD), Department of Paediatrics (A Wander DM), All India Institute of Medical Sciences, Bathinda, India; IRCCS Fondazione Don Carlo Gnocchi, Milan, Italy (P Pedersini MSc); International Institute for Educational Planning (IIEP) (Prof M F P Peres MD), Albert Einstein Hospital, São Paulo, Brazil; Department of Food, Environmental and Nutritional Sciences (Prof S Perna PhD), University of Milan, Milan, Italy; CMH Lahore Medical College (W Perveen MS), CMH Lahore Medical College & Institute of Dentistry, Lahore, Pakistan; Center of Excellence in Behavioral Medicine (H Q Pham MD), Nguyen Tat Thanh University, Ho Chi Minh City, Vietnam; Department of Neurology (Prof S Prakash DM), Smt. B.K.S. Medical Institute and Research Center, Vadodara, India; Department of Biochemistry (Prof A Prashant PhD), JSS Academy of Higher Education and Research, Mysuru, India; Department of Biostatistics, Epidemiology, and Informatics (J Puvvula PhD), University of Pennsylvania, Philadelphia, PA, USA; Division of Psychology and Mental Health (M R Radojčić PhD), University of Manchester, Manchester, UK; Department of Biostatistics (Prof M Rahman MS), National Institute of Preventive and Social Medicine, Dhaka, Bangladesh; Future Technology Research Center (A Rahmani PhD), National Yunlin University of Science and Technology, Yunlin, Taiwan; Department of Cardiology (Prof M M Ramadan PhD), Faculty of Pharmacy (Prof M A Saleh PhD), Mansoura University, Mansoura, Egypt; Department of Family Medicine (Prof D Rathish PhD), Rajarata University of Sri Lanka, Anuradhapura, Sri Lanka; Academic Public Health England (Prof S Rawaf MD), Public Health England, London, UK; Department of Epidemiology and Biostatistics (Prof M

Rezaeian PhD), Rafsanjan University of Medical Sciences, Rafsanjan, Iran; Department of Public Health Sciences (T Rhee PhD), University of Connecticut, Farmington, CT, USA; Department of Psychiatry (T Rhee PhD), Yale University, New Haven, CT, USA; Faculty of Nursing (D S Romadlon PhD), Chulalongkorn University, Bangkok, Thailand; Department of Neurosciences (M Romoli MD), Maurizio Bufalini Hospital, Cesena, Italy; Fondazione Policlinico Universitario A. Gemelli (M Romozzi MD), Cuore Università Cattolica del Sacro Cuore (Catholic University of Sacred Heart), Rome, Italy; Széchenyi István University, Győr, Hungary (Prof U Saeed PhD); Operational Research Center in Healthcare (Prof U Saeed PhD), Near East University, Cyprus, Turkiye; Biotechnology Research Center (Prof A Sahebkar PhD), Mashhad University of Medical Sciences, Mashhad, Iran; Department of Entomology (A M Samy PhD), Medical Ain Shams Research Institute (MASRI) (A M Samy PhD), Ain Shams University, Cairo, Egypt; University of São Paulo City, São Paulo, Brazil (L H C C Santos MSc); Department of Pharmacology (A Saravanan MD), All India Institute of Medical Sciences, Jodhpur, India; Indira Gandhi Medical College and Research Institute, Puducherry, India (A Saravanan MD); Botany Department (H Sarma PhD), Bodoland University, Kokrajhar, India; Department of Neurosurgery (Y Senol MD), Department of Bioengineering and Therapeutical Sciences (Prof M Zastrozhin PhD), University of California San Francisco, San Francisco, CA, USA; Department of Medicine (Y Sethi MD), Swami Vivekanand Subharti University, Meerut, India; Faculty of Medicine (Y K F S Shaalan MD), Misr University for Science and Technology, 6th of October city, Egypt; Centre For Interdisciplinary Research In Basic Sciences (CIRBSc) (A Shamsi PhD), Jamia Millia Islamia, New Delhi, India; Lancaster University, Lancaster, UK (D Shan PhD); Department for Evidence-based Medicine and Evaluation (A Sharifan PharmD), University for Continuing Education Krems, Krems, Austria; Department of Pharmacology (R R Shenoy PhD), Manipal College of Dental Sciences, Mangalore (Prof P K Shetty MDS), Manipal Academy of Higher Education, Manipal, India; Alimentary Tract Research Center (Z Shokati Eshkiki PhD), Ahvaz Jundishapur University of Medical Sciences, Ahvaz, Iran; Department of Research and Academics (S Shrestha PhD), Kathmandu Cancer Center, Bhaktapur, Nepal; Person-Centered Research (S Shrestha PhD), Monash University, Box Hill, VIC, Australia; Department of Pharmacology (H Singh DM), Government Medical College and Hospital, Chandigarh, India; School of Medicine (Prof J A Singh MD), Baylor College of Medicine, Houston, TX, USA; Department of Medicine Service (Prof J A Singh MD), US Department of Veterans Affairs (VA), Houston, TX, USA; Books Committee (V Y Skryabin MD), Royal College of Psychiatrists, London, UK; Department of Public Health (F Sobia PhD), Jazan University, Jazan, Saudi Arabia; Division of Preventive Medicine (Prof S Straube DPhil), School of Public Health (Prof S Straube DPhil), University of Alberta, Edmonton, AB, Canada; Department of Analytical and Applied Economics (C Swain MPhil), Utkal University, Bhubaneswar, India; Department of Neurology (P Tabaee Damavandi MD), Neurocenter of Southern Switzerland (NSI), Lugano, Switzerland; Department of Computer and Software Engineering (M Tanveer PhD), National University of Science and Technology (NUST), Islamabad, Pakistan; Department of Psychiatry (M Tareke MSc), Department of Pharmacology (Y E Yismaw MSc), Bahir Dar University, Bahir Dar, Ethiopia; School of Medicine at the Federal University of Minas Gerais (UFMG) (C B Tavares MSc), Federal University of Minas Gerais, Belo Horizonte, Brazil; Department of Clinical Medicine of the Fluminense Federal University (C B Tavares MSc), Fluminense Federal University, Rio de Janeiro, Brazil; College of Medicine (Prof M Temsah MD), Alfaisal University, Riyadh, Saudi Arabia; Department of Preventive Medicine (M Teramoto MD), Northwestern University, Chicago, IL, USA; International Centre for Eye Health (A J Thirunavukarasu MA), London School of Hygiene & Tropical Medicine, London, UK; Department of Neurology (A Tiwari DM), All India Institute of Medical Sciences, Gorakhpur, India; Department of Internal Medicine (T H Tran MD), University of Medicine and Pharmacy at Ho Chi Minh City, Ho Chi Minh

City, Vietnam; Department of Business Analytics (T H Tran MD), University of Massachusetts Dartmouth, Dartmouth, MA, USA; Research and Advocacy Initiative (N Tran Minh Duc MD), ALS Vietnam, Quang Ngai, Vietnam; Laboratory of Clinical Pharmacology (V Tseriotis MSc), Aristotle University of Thessaloniki, Thessaloniki, Greece; Department of Epidemiology and Public Health (V Tseriotis MSc), University College London, London, UK; ESIC Medical College PGIMSR and Model Hospital, Bengaluru, India (S Tumkur Narayanappa BSc); ESIC Alumni Association, Bengaluru, India (S Tumkur Narayanappa BSc); Department of Biosciences and Biotechnology (A J Udoakang PhD), University of Medical Sciences, Ondo, Ondo, Nigeria; Hayatabad Medical Complex (H Ullah FCPS), Postgraduate Medical Institute, Peshawar, Pakistan; Department of Rehabilitation Sciences (J S Usman PhD), Hong Kong Polytechnic University, Hong Kong, China; Department of Psychiatry (A Usman Sambo FWACP), Federal Neuropsychiatric Hospital, Kaduna, Nigeria; Department of Cardiovascular Sciences (J Van den Eynde BSc), Katholieke Universiteit Leuven, Leuven, Belgium; UKK Institute, Tampere, Finland (Prof T J Vasankari PhD); Faculty of Medicine and Health Technology (Prof T J Vasankari PhD), Tampere University, Tampere, Finland; Raffles Neuroscience Centre (Prof N Venketasubramanian MSc), Raffles Hospital, Singapore, Singapore; Yong Loo Lin School of Medicine (Prof N Venketasubramanian MSc), National University of Singapore, Singapore, Singapore; Department of Physiotherapy (J H Villafañe PhD), Universidad Europea de Madrid (European University of Madrid), Villaviciosa de Odón, Spain; School of Public Health (Prof W Wang PhD), Xuzhou Medical University, Xuzhou, China; Shandong University of Traditional Chinese Medicine (X Wang MD), Shandong University of Traditional Chinese Medicine, Jinan, China; Department of Physical Therapy (T Wiangkham PhD), Naresuan University, Phitsanulok, Thailand; Department of Experimental Dentistry (Prof M Wieckiewicz PhD), Wroclaw Medical University, Wroclaw, Poland; Department of Nutrition (W Xu MPH), Tufts University, Boston, MA, USA; Department of Community Medicine (S Yahoo (Syed) MD), Apollo Institute of Medical Sciences and Research, Hyderabad, India; Pharmacy Department (Y E Yismaw MSc), Alkan Health Science, Business and Technology College, Bahir Dar, Ethiopia; Department of Pediatrics (Prof D Yon MD), Kyung Hee University, Seoul, South Korea; Department of Biostatistics (Prof N Yonemoto PhD), University of Toyama, Toyama, Japan; Department of Public Health (Prof N Yonemoto PhD), Juntendo University, Tokyo, Japan; Department of Public Health (A Yousuf PhD), Jigjiga University, Jigjiga, Ethiopia; Sant'Elia Hospital (A Zanghì MD), University of Catania, Caltanissetta, Italy; Department of Administration (Prof M Zastrozhin PhD), PGxAI, San Francisco, CA, USA; School of Health and Biomedical Sciences (Prof A L Zhang PhD), Royal Melbourne Institute of Technology (RMIT) University, Melbourne, VIC, Australia; Department of Health Management (Z Zhao PhD), Shengjing Hospital of China Medical University, Shenyang, China; Department of Biochemistry and Pharmacogenomics (M Zielińska MPharm), Medical University of Warsaw, Warsaw, Poland.

Authors' Contributions

Managing the overall research enterprise

Christopher J L Murray, Kanyin Liane Ong, Jaimie D Steinmetz, and Theo Vos.

Writing the first draft of the manuscript

Andreas Kattem Husøy.

Primary responsibility for applying analytical methods to produce estimates

Andreas Kattem Husøy and Yvonne Yiru Xu.

Primary responsibility for seeking, cataloguing, extracting, or cleaning data; designing or coding figures and tables

Andreas Kattem Husøy and Yvonne Yiru Xu.

Providing data or critical feedback on data sources

Mohammad Amin Aalipour, Richard Gyan Aboagye, Samir Abu Rumeileh, Salahdein Aburuz, Qorinah Estiningtyas Sakilah Adnani, Ali Ahmed, Asma Ahmed, Shahzaib Ahmed, Sawsan Alabbad, Mohammed Albashtawy, Ashraf Alhumaidi, Syed Shujait Ali, Montaha Al-Iede, Joseph Uy Almazan, Awais Altaf, Mohammad Al-Wardat, Jalal Arabloo, Mahsa Asadi Anar, Khursheed Aurangzeb, Arian Azadnia, Ahmed Y Azzam, Mohammad-Mahdi Bastan, Jina Behjati, Sonu Bhaskar, Gurjit Kaur Bhatti, Jasvinder Singh Bhatti, Bijit Biswas, Archith Boloor, Meriem Boukhiam, Edoardo Caronna, Vijay Kumar Chattu, Hongyuan Chu, Josielli Comachio, Natalia Cruz-Martins, Xiaochen Dai, Anh Kim Dang, Lucio D'Anna, Sindhura Deekonda, Pouria Delbari, Andreas K Demetriades, Vinoth Gnana Chellaiyan Devanbu, Xueting Ding, Huyen Phuc Do, Ojas Prakashbhai Doshi, Lamiaa Labieb Mahmoud Ebraheim, Ebrahim Eini, Michael Ekholuenetale, Andre Faro, Ginenus Fekadu, Seyed-Mohammad Fereshtehnejad, Arianna Fornari, Matteo Foschi, Abdelrahman Gamil Gad, David Garcia-Azorin, Zisis Gatzioufas, Massimiliano Gobbo, Mahaveer Golechha, Pouya Goleij, Ishita Gupta, Sapna Gupta, Najah R Hadi, Victoria Funmilayo Hanson, Simon I Hay, Jeffrey J Hebert, Mehdi Hoseinzadeh, Md. Jubayer Hossain, Yongsong Huang, Luigi Francesco Iannone, Segun Emmanuel Ibitoye, Olayinka Stephen Ilesanmi, Muhana Fawwazy Ilyas, Salim Ilyasu, Md Sahidul Islam, Nahlah Elkudssiah Ismail, Haitham Jahrami, Jost B Jonas, Charity Ehimwenma Joshua, Saltanat Kamenova, Rami S Kantar, Mohmed Isaqali Karobari, Himanshu Khajuria, Sameer Uttamaro Khasbage, Yun Jin Kim, Aida Kondybayeva, Kewal Krishan, Barthelemy Kuate Defo, Chandan Kumar, Vijay Kumar, Ville Kytö, Caterina Ledda, Seung Won Lee, Yanxue Lian, Jay B Lusk, Sasikumar Mahalingam, Rituparna Maiti, Ahmad Azam Malik, Birhanemaskal Malkamu, Roy Rillera Marzo, Atte Meretoja, Archana Mishra, Arup Kumar Misra, Khabab Abbasher Hussien Mohamed Ahmed, Shafiu Mohammed, Ali H Mokdad, Mahdis Morovvati, Yanjinlkham Munkhsaikhan, Efren Murillo-Zamora, Christopher J L Murray, Ghulam Mustafa, Zuhair S Natto, Cao Duy Nguyen, Cuong Tat Nguyen, Hien Thu Nguyen, Long Nguyen, Robina Khan Niazi, Fred Nugen, Chijindu N Nwakama, Bogdan Oancea, Michael Safo Oduro, Andrew T Olagunju, Kanyin Liane Ong, Mayowa O Owolabi, Mahesh P A, Leonidas D Panos, Shahina Pardhan, Romil R Parikh, Arpit Parmar, Shankargouda Patil, Apurba Patra, Mario F P Peres, Simone Perna, Hai Quang Pham, Jagadeesh Puvvula, Mohammad Meshbahur Rahman, Amir Masoud Rahmani, Mahmoud Mohammed Ramadan, Salman Rawaf, Taeho Gregory Rhee, Debby Syahru Romadlon, Michele Romoli, Marina Romozzi, Umar Saeed, Abdallah M Samy, Yigit Can Senol, Yashendra Sethi, Anas Shamsi, Dan Shan, Amin Sharifan, Premalatha K Shetty, Zahra Shokati Eshkiki, Sunil Shrestha, Harmanjit Singh, Jasvinder A Singh, Valentin Yurievich Skryabin, Reed J D Sorensen, Timothy J Steiner, Chandan Kumar Swain, Sree Sudha T Y, Celine Tabche, Nguyen Tran Minh Duc, Santhosh Kumar Tumkur Narayanappa, Himayat Ullah, Jef Van den Eynde, Tommi Juhani Vasankari, Narayanaswamy Venketasubramanian, Arvinder Wander, Xingxin Wang, Taweewat Wiangkham, Wanqing Xu, Yvonne Yiru Xu, Naohiro Yonemoto, Abdilahi Yousuf, Michael Zastrozhin, and Zhongyi Zhao.

Developing methods or computational machinery

Aleksandr Y Aravkin, Xiaochen Dai, Simon I Hay, Ali H Mokdad, Christopher J L Murray, Kanyin Liane Ong, Reed J D Sorensen, and Yvonne Yiru Xu.

Providing critical feedback on methods or results

Mohammad Amin Aalipour, Deldar Morad Abdulah, Richard Gyan Aboagye, Dariush Abtahi, Samir Abu Rumeileh, Salahdein Aburuz, Qorinah Estiningtyas Sakilah Adnani, Obed Adonteng-Kissi, Giuseppina Affinito, Danish Ahmad, Ali Ahmed, Asma Ahmed, Shahzaib Ahmed, Mohammad Ahmmad Mahmoud Al Zoubi, Sawsan Alabbad, Yazan Al-Ajlouni, Mohammed Albashtawy, Ashraf Alhumaidi, Mohammed Usman Ali, Syed Shujait Ali, Montaha Al-Iede, Joseph Uy Almazan, Najim Z. Alshahrani, Awais Altaf, Mohammad Al-Wardat, Yaser Mohammed Al-Worafi, Karem H Alzoubi, Sohrab Amiri, Ganiyu Adeniyi Amusa, David B Anderson, Jalal Arabloo, Demelash Areda, Mahsa Asadi Anar, Mohammad Asghari-Jafarabadi, Khursheed Aurangzeb, Arian Azadnia, Ahmed Y Azzam, Youngoh Bae, Razieh Bahreini, Hiba Jawdat Barqawi, Rehana Basri, Mohammad-Mahdi Bastan, Jina Behjati, Maryam Bemanalizadeh, Jeetendra Bhandari, Sonu Bhaskar, Gurjit Kaur Bhatti, Jasvinder Singh Bhatti, Rajbir Bhatti, Bijit Biswas, Bruno Bizzozero-Peroni, Archith Boloor, Meriem Boukhiam, Yasser Bustanji, Luis Alberto Cámera, Ana Paula Carvalho-e-Silva, Vijay Kumar Chattu, Hongyuan Chu, Josielli Comachio, Daniela Contreras, Natalia Cruz-Martins, Omid Dadras, Xiaochen Dai, Anh Kim Dang, Lucio D'Anna, Sindhura Deekonda, Pouria Delbari, Andreas K Demetriades, Emina Dervišević, Vinoth Gnana Chellaiyan Devanbu, Amol S Dhane, Bibha Dhungel, Xueting Ding, Huyen Phuc Do, Ojas Prakashbhai Doshi, Siddhartha Dutta, Lamiaa Labieb Mahmoud Ebraheim, Ebrahim Eini, Michael Ekholuenetale, Sharareh Eskandarieh, Andre Faro, Valery L Feigin, Gelana Fekadu, Ginenus Fekadu, Seyed-Mohammad Fereshtehnejad, Pietro Ferrara, Claudio Fiorilla, Florian Fischer, Arianna Fornari, Celia Fortuna Rodrigues, Matteo Foschi, Abdelrahman Gamil Gad, Márió Gajdács, David Garcia-Azorin, Zisis Gatzioufas, Rupesh K Gautam, Miglas Welay Gebregergis, Massimiliano Gobbo, Mahaveer Golechha, Alessandra C Goulart, Ishita Gupta, Sapna Gupta, Roberth Steven Gutiérrez-Murillo, Najah R Hadi, Faraidoon Haghdoost, Mohamed Hamed, Victoria Funmilayo Hanson, Simon I Hay, Khezar Hayat, Bartosz Helfer, Mehdi Hoseinzadeh, Md. Jubayer Hossain, Yongsong Huang, Luigi Francesco Iannone, Segun Emmanuel Ibitoye, Olayinka Stephen Ilesanmi, Irena M Ilic, Muhana Fawwazy Ilyas, Salim Ilyasu, Md Rabiul Islam, Md Sahidul Islam, Nahlah Elkudssiah Ismail, Louis Jacob, Haitham Jahrami, Ammar Abdulrahman Jairoun, Navid Jamali, Ruwan Duminda Jayasinghe, Shuai Jin, Jost B Jonas, Nitin Joseph, Charity Ehimwenma Joshua, Saltanat Kamenova, Rami S Kantar, Sujita Kumar Kar, Mohmed Isaqali Karobari, Himanshu Khajuria, Sameer Uttamaro Khasbage, Yun Jin Kim, Omid Kohandel Gargari, Farzad Kompani, Aida Kondybayeva, Kewal Krishan, Barthelemy Kuate Defo, Chandan Kumar, Vijay Kumar, Ville Kytö, Caterina Ledda, Seung Won Lee, Jacopo Lenzi, Jianan Li, Linyan Li, Yanxue Lian, Giancarlo Lucchetti, Jay B Lusk, Sasikumar Mahalingam, Rituparna Maiti, Ahmad Azam Malik, Birhanemaskal Malkamu, Vahid Mansouri, Konstantinos Margetis, Roy Rillera Marzo, Yasith Mathangasinghe, Hadush Negash Meles, Atte Meretoja, Tomislav Mestrovic, Sachith Mettananda, Bartosz Miazgowski, Giuseppe Minervini, Archana Mishra, Khabab Abbasher Hussien Mohamed Ahmed, Omer Mohammed, Shafiu Mohammed, Ali H Mokdad, Shaher Momani, Maziar Moradi-Lakeh, Mahdis Morovvati, Reza Mosaddeghi Heris, Yanjinlkham Munkhsaikhan, Efren Murillo-Zamora, Christopher J L Murray, Ghulam Mustafa, Fatemehzahra Naddafi, Zuhair S Natto, Gaurav Nepal, Charles Richard James Newton, Cao Duy Nguyen, Cuong Tat Nguyen, Hien Thu Nguyen, Long Nguyen, Robina Khan Niazi, Luciano Nieddu, Fred Nugen, Chijindu N Nwakama, Bogdan Oancea, Michael Safo Oduro, Hassan Okati-Aliabad, Andrew T Olagunju, Arão Belitardo Oliveira, Kanyin Liane Ong, Jia Ouyang, Mark Overton, Mayowa O Owolabi, Mahesh P A, Giuseppina Palena, Leonidas D Panos, Ioannis Pantazopoulos, Shahina Pardhan, Romil R Parikh, Arpit Parmar, Maja Pasovic, Shankargouda Patil, Apurba Patra, Paolo Pedersini, Mario F P Peres, Simone Perna, Hai Quang Pham, Akila Prashant, Jagadeesh Puvvula, Maja R Radojčić, Alberto Raggi, Mohammad Meshbahur Rahman, Amir Masoud Rahmani, Mahmoud Mohammed

Ramadan, Devarajan Rathish, Salman Rawaf, Mohsen Rezaeian, Taeho Gregory Rhee, Debby Syahru Romadlon, Michele Romoli, Marina Romozzi, Umar Saeed, Amene Saghazadeh, Mohamed A Saleh, Sohrab Salimi, Abdallah M Samy, Lucas H C C Santos, Hemen Sarma, Yigit Can Senol, Yashendra Sethi, Yara Khaled Fouad Sayed Shaalan, Wajeehah Shahid, Anas Shamsi, Dan Shan, Amin Sharifan, Rekha Raghuveer Shenoy, Zahra Shokati Eshkiki, Sunil Shrestha, Harmanjit Singh, Jasvinder A Singh, Satwinder Singh, Valentin Yurievich Skryabin, Farrukh Sobia, Reed J D Sorensen, Timothy J Steiner, Jaimie D Steinmetz, Sebastian Straube, Chandan Kumar Swain, Sree Sudha T Y, Payam Tabaee Damavandi, Celine Tabche, Mohsan Tanveer, Minale Tareke, Claudia Baptista Tavares, Mohamad-Hani Temsah, Masayuki Teramoto, Arun James Thirunavukarasu, Ashutosh Tiwari, Nguyen Tran Minh Duc, Vasilis-Spyridon Tseriotis, Santhosh Kumar Tumkur Narayanappa, Aniefiok John Udoakang, Himayat Ullah, Jibrin Sammani Usman, Abdulkadir Usman Sambo, Jef Van den Eynde, Narayanaswamy Venketasubramanian, Jorge Hugo Villafañe, Arvinder Wander, Wei Wang, Xingxin Wang, Yuan-Pang Wang, Taweewat Wiangkham, Mieszko Wieckiewicz, Wanqing Xu, Yvonne Yiru Xu, Saba Yahoo (Syed), Dong Keon Yon, Naohiro Yonemoto, Abdilahi Yousuf, Michael Zastrozhin, Anthony Lin Zhang, and Zhongyi Zhao.

Drafting the work or revising it critically for important intellectual content

Mohammad Amin Aalipour, Hasan Aalruz, Samir Abu Rumeileh, Salahdein Aburuz, Qorinah Estiningtyas Sakilah Adnani, Obed Adonteng-Kissi, Danish Ahmad, Negar Sadat Ahmadi, Ali Ahmed, Asma Ahmed, Shahzaib Ahmed, Mohammad Ahmmad Mahmoud Al Zoubi, Sawsan Alabbad, Yazan Al-Ajlouni, Mohammed Albashtawy, Fadwa Naji Alhalaiqa, Ashraf Alhumaidi, Mohammed Usman Ali, Syed Shujait Ali, Montaha Al-Iede, Najim Z. Alshahrani, Awais Altaf, Mohammad Al-Wardat, Yaser Mohammed Al-Worafi, Karem H Alzoubi, Sohrab Amiri, Ganiyu Adeniyi Amusa, David B Anderson, Abhishek Anil, Jalal Arabloo, Sait Ashina, Arian Azadnia, Ahmed Y Azzam, Youngoh Bae, Soham Bandyopadhyay, Hiba Jawdat Barqawi, Azadeh Bashiri, Mohammad-Mahdi Bastan, Jina Behjati, Maryam Bemanalizadeh, Sonu Bhaskar, Gurjit Kaur Bhatti, Jasvinder Singh Bhatti, Bijit Biswas, Bruno Bizzozero-Peroni, Meriem Boukhiam, Yasser Bustanji, Sanjay C J, Edoardo Caronna, Ana Paula Carvalho-e-Silva, Sandip Chakraborty, Vijay Kumar Chattu, Anis Ahmad Chaudhary, Patrick R Ching, Hongyuan Chu, Josielli Comachio, Daniela Contreras, Natalia Cruz-Martins, Emanuele D'Amico, Anh Kim Dang, Lucio D'Anna, Sindhura Deekonda, Pouria Delbari, Andreas K Demetriades, Emina Dervišević, Vinoth Gnana Chellaiyan Devanbu, Amol S Dhane, Xueting Ding, Huyen Phuc Do, Ojas Prakashbhai Doshi, Siddhartha Dutta, Lamiaa Labieb Mahmoud Ebraheim, Ebrahim Eini, Michael Ekholuenetale, Sharareh Eskandarieh, Andre Faro, Valery L Feigin, Gelana Fekadu, Seyed-Mohammad Fereshtehnejad, Abdullah H Feroze, Pietro Ferrara, Nuno Ferreira, Claudio Fiorilla, Florian Fischer, Celia Fortuna Rodrigues, Matteo Foschi, Márió Gajdács, David Garcia-Azorin, Zisis Gatzioufas, Rupesh K Gautam, Miglas Welay Gebregergis, Elena V Gnedovskaya, Massimiliano Gobbo, Alessandra C Goulart, Ishita Gupta, Sapna Gupta, Roberth Steven Gutiérrez-Murillo, Najah R Hadi, Faraidoon Haghdoost, Mohamed Hamed, Victoria Funmilayo Hanson, Amr Hassan, Simon I Hay, Khezar Hayat, Jeffrey J Hebert, Golnaz Heidari, Bartosz Helfer, Md. Jubayer Hossain, Yongsong Huang, Luigi Francesco Iannone, Segun Emmanuel Ibitoye, Olayinka Stephen Ilesanmi, Irena M Ilic, Muhana Fawwazy Ilyas, Salim Ilyasu, Md Rabiul Islam, Md Sahidul Islam, Nahlah Elkudssiah Ismail, Louis Jacob, Haitham Jahrami, Manthan Dilipkumar Janodia, Ruwan Duminda Jayasinghe, Shuai Jin, Jost B Jonas, Nitin Joseph, Charity Ehimwenma Joshua, Saltanat Kamenova, Arun Kamireddy, Rami S Kantar, Mohmed Isaqali Karobari, Himanshu Khajuria, Sameer Uttamaro Khasbage, Jagdish Khubchandani, Omid Kohandel Gargari, Aida Kondybayeva, Kewal Krishan, Barthelemy Kuate Defo, Mukhtar Kulimbet, Chandan Kumar, Rakesh Kumar, Caterina Ledda, Jacopo Lenzi, Linyan Li, Yanxue Lian, Giancarlo Lucchetti, Jay B Lusk, Ricardo Lutzky Saute, Sasikumar Mahalingam, Rituparna

Maiti, Ahmad Azam Malik, Vahid Mansouri, Konstantinos Margetis, Roy Rillera Marzo, Yasith Mathangasinghe, Georgios Mavrovounis, Hadush Negash Meles, Atte Meretoja, Tomislav Mestrovic, Sachith Mettananda, Archana Mishra, Khabab Abbasher Hussien Mohamed Ahmed, Omer Mohammed, Shafiu Mohammed, Ali H Mokdad, Maziar Moradi-Lakeh, Mahdis Morovvati, Reza Mosaddeghi Heris, Kavita Munjal, Yanjinlkham Munkhsaikhan, Efren Murillo-Zamora, Christopher J L Murray, Ghulam Mustafa, Fatemehzahra Naddafi, Zuhair S Natto, Gaurav Nepal, Charles Richard James Newton, Cao Duy Nguyen, Cuong Tat Nguyen, Hien Thu Nguyen, Long Nguyen, Robina Khan Niazi, Luciano Nieddu, Fred Nugen, Chijindu N Nwakama, Ogochukwu Janet Nzoputam, Bogdan Oancea, Andrew T Olagunju, Arão Belitardo Oliveira, Kanyin Liane Ong, Jia Ouyang, Mark Overton, Mayowa O Owolabi, Mahesh P A, Leonidas D Panos, Ioannis Pantazopoulos, Shahina Pardhan, Romil R Parikh, Shankargouda Patil, Paolo Pedersini, Mario F P Peres, Simone Perna, Wajida Perveen, Hai Quang Pham, Sanjay Prakash, Akila Prashant, Jagadeesh Puvvula, Maja R Radojčić, Alberto Raggi, Mohammad Meshbahur Rahman, Mahmoud Mohammed Ramadan, Devarajan Rathish, Salman Rawaf, Debby Syahru Romadlon, Marina Romozzi, Umar Saeed, Amirhossein Sahebkar, Abdallah M Samy, Lucas H C C Santos, Aswini Saravanan, Yigit Can Senol, Yashendra Sethi, Anas Shamsi, Dan Shan, Amin Sharifan, Rekha Raghuveer Shenoy, Premalatha K Shetty, Sunil Shrestha, Harmanjit Singh, Jasvinder A Singh, Satwinder Singh, Valentin Yurievich Skryabin, Farrukh Sobia, Timothy J Steiner, Sebastian Straube, Chandan Kumar Swain, Sree Sudha T Y, Payam Tabaee Damavandi, Celine Tabche, Minale Tareke, Claudia Baptista Tavares, Mohamad-Hani Temsah, Masayuki Teramoto, Arun James Thirunavukarasu, Ashutosh Tiwari, Thang Huu Tran, Nguyen Tran Minh Duc, Vasilis-Spyridon Tseriotis, Santhosh Kumar Tumkur Narayanappa, Aniefiok John Udoakang, Himayat Ullah, Jibrin Sammani Usman, Abdulkadir Usman Sambo, Jef Van den Eynde, Tommi Juhani Vasankari, Narayanaswamy Venketasubramanian, Jorge Hugo Villafañe, Wei Wang, Xingxin Wang, Yuan-Pang Wang, Taweewat Wiangkham, Mieszko Wieckiewicz, Wanqing Xu, Yvonne Yiru Xu, Saba Yahoo (Syed), Yazachew Engida Yismaw, Dong Keon Yon, Naohiro Yonemoto, Aurora Zanghì, Michael Zastrozhin, Zhongyi Zhao, and Magdalena Zielińska.

Managing the estimation or publications process

Hailey Hagins, Simon I Hay, Ali H Mokdad, Christopher J L Murray, Maja Pasovic, Jaimie D Steinmetz, and Yvonne Yiru Xu.
